# Open-Label Comparative Clinical Study of Chlorproguanil−Dapsone Fixed Dose Combination (Lapdap™) Alone or with Three Different Doses of Artesunate for Uncomplicated *Plasmodium falciparum* Malaria

**DOI:** 10.1371/journal.pone.0001779

**Published:** 2008-03-05

**Authors:** Daniel G. Wootton, Hyginus Opara, Giancarlo A. Biagini, Maxwell K. Kanjala, Stephan Duparc, Paula L. Kirby, Mary Woessner, Colin Neate, Maggie Nyirenda, Hannah Blencowe, Queen Dube-Mbeye, Thomas Kanyok, Stephen Ward, Malcolm Molyneux, Sam Dunyo, Peter A. Winstanley

**Affiliations:** 1 Department of Pharmacology & Therapeutics, University of Liverpool, Liverpool, United Kingdom; 2 Malawi-Liverpool-Wellcome Trust Major Overseas Programme, College of Medicine, Blantyre, Malawi; 3 MRC Laboratories, Banjul, Fajara, The Gambia; 4 Liverpool School of Tropical Medicine, Liverpool, United Kingdom; 5 GlaxoSmithKline, Greenford, Middlesex, United Kingdom; 6 Department of Paediatrics, College of Medicine, Blantyre, Malawi; 7 Global Health-Infectious Diseases, Bill and Melinda Gates Foundation, Seattle, Washington, United States of America (formerly at the WHO-UNDP-World Bank Special Programme for Research and Training in Tropical Medicine (WHO-TDR), Geneva, Switzerland); London School of Hygiene & Tropical Medicine, United Kingdom

## Abstract

The objective of this study was to determine the appropriate dose of artesunate for use in a fixed dose combination therapy with chlorproguanil−dapsone (CPG−DDS) for the treatment of uncomplicated falciparum malaria.

**Methods:**

Open-label clinical trial comparing CPG−DDS alone or with artesunate 4, 2, or 1 mg/kg at medical centers in Blantyre, Malawi and Farafenni, The Gambia. The trial was conducted between June 2002 and February 2005, including 116 adults (median age 27 years) and 107 children (median age 38 months) with acute uncomplicated *Plasmodium falciparum* malaria. Subjects were randomized into 4 groups to receive CPG–DDS alone or plus 4, 2 or 1 mg/kg of artesunate once daily for 3 days. Assessments took place on Days 0−3 in hospital and follow-up on Days 7 and 14 as out-patients. Efficacy was evaluated in the Day 3 per-protocol (PP) population using mean time to reduce baseline parasitemia by 90% (PC90). A number of secondary outcomes were also included. Appropriate artesunate dose was determined using a pre-defined decision matrix based on primary and secondary outcomes. Treatment emergent adverse events were recorded from clinical assessments and blood parameters. Safety was evaluated in the intent to treat (ITT) population.

**Results:**

In the Day 3 PP population for the adult group (N = 85), mean time to PC90 was 19.1 h in the CPG−DDS group, significantly longer than for the +artesunate 1 mg/kg (12.5 h; treatment difference −6.6 h [95%CI −11.8, −1.5]), 2 mg/kg (10.7 h; −8.4 h [95%CI −13.6, −3.2]) and 4 mg/kg (10.3 h; −8.7 h [95%CI −14.1, −3.2]) groups. For children in the Day 3 PP population (N = 92), mean time to PC90 was 21.1 h in the CPG−DDS group, similar to the +artesunate 1 mg/kg group (17.7 h; −3.3 h [95%CI −8.6, 2.0]), though the +artesunate 2 mg/kg and 4 mg/kg groups had significantly shorter mean times to PC90 versus CPG−DDS; 14.4 h (treatment difference −6.4 h [95%CI −11.7, −1.0]) and 12.8 h (−7.4 h [95%CI −12.9, −1.8]), respectively. An analysis of mean time to PC90 for the Day 14 PP and ITT populations was consistent with the primary analysis. Treatment emergent, drug-related adverse events were experienced in 35.3% (41/116) of adults and 70.1% (75/107) of children; mostly hematological and gastroenterological. The nature and incidence of adverse events was similar between the groups. No dose-related changes in laboratory parameters were observed. Nine serious adverse events due to any cause occurred in five subjects including two cases of hemolysis believed to be associated with drug treatment (one adult, one child). One adult died of anaphylactic shock, not associated with investigational therapy.

**Conclusions:**

CPG–DDS plus artesunate demonstrated advantages over CPG–DDS alone for the primary efficacy endpoint (mean time to PC90) except in children for the 1 mg/kg artesunate dose. Based on a pre-defined decision matrix, the primary endpoint in the child group supported an artesunate dose of 4 mg/kg. Secondary endpoints also supported a 4 mg/kg artesunate dose to take forward into the remainder of the development program.

**Trial Registration:**

ClinicalTrials.gov NCT00519467

## Introduction

Over one million people die of malaria every year, mostly children [1]. Widespread drug resistance in *Plasmodium* spp. has undermined the effectiveness of many of the antimalarials used commonly, particularly chloroquine [2]. Combination therapy is required to combat these resistant strains and preserve antimalarial effectiveness. It is important that new combinations include agents with different mechanisms of action used at appropriate doses to ensure maximal and rapid parasite killing.

In consideration of these points, in 2004 the World Health Organization (WHO) Roll Back Malaria group recommended that first-line treatment of uncomplicated malaria should be with artemisinin-based combination therapy [3]. Artemisinin-derived drugs such as artesunate and artemether are effective against multi-drug resistant strains of *P. falciparum* [4], [5], and are highly potent against asexual forms, rapidly reducing parasitemia [6], [7]. In addition, these agents are active against gametocytes, potentially reducing transmission rates [8]–[12]. Current evidence suggests that these agents are well tolerated in humans at doses under 15 mg/kg, though they are contra-indicated in early pregnancy [13]. However, effective monotherapy requires seven days of treatment, increasing the risk of resistance development through poor compliance and so these agents must be combined with at least one other antimalarial.

Chlorproguanil−dapsone (CPG−DDS, Lapdap™) was developed through a public−private partnership as a low-cost, fixed dose combination for the treatment of uncomplicated malaria due to *Plasmodium falciparum* [14]. In a study of 1850 children from five African countries, CPG−DDS had greater efficacy than sulfadoxine-pyrimethamine and was generally well tolerated, though hematological adverse effects were more common in the CPG–DDS group [15]. CPG–DDS is rapidly eliminated and has been shown to exert a reduced selective pressure for resistance on *P. falciparum* compared with the more slowly eliminated combination sulfadoxine-pyrimethamine with no increase in the incidence of retreatment [16], [17].

A clinical program to develop CPG−DDS−artesunate (CDA) is currently in progress. CDA is an interesting combination for investigation as the three compounds have well-matched pharmacokinetics, i.e. all are relatively rapidly eliminated. Thus, there is a reduced risk of parasite exposure to any single compound after elimination of the partner drug, potentially decreasing the risk of resistance emergence.

This study was a phase II trial to determine the most appropriate dose of artesunate for use in combination with CPG–DDS. Four dosing groups were included: CPG–DDS alone or with 4, 2, or 1 mg/kg artesunate. The WHO-defined ‘adequate clinical and parasitological response’ with CPG–DDS alone has been reported as 96% in African children at 14-days' follow up [15]. Owing to this high efficacy rate, it would be impractical to determine any additional efficacy with artesunate over CPG−DDS alone using this outcome measure in a four arm study.

As artesunate rapidly clears parasites from the blood, the protocol development committee concluded that parasitemia-derived indices of therapeutic response, in particular time to reduce parasitemia by 90%, would be the most relevant alternative outcomes to detect differences between the artesunate dose groups and CPG−DDS alone. A suite of other outcomes was also included. Children are the primary population of interest because of the higher mortality risk compared with adults [1]. However, to determine drug effects on parasitemia, frequent blood sampling is required over the first 48 h after dosing. Owing to the difficulties of conducting the repeat sampling required in children, adults were also included in the study to provide a more complete set of time points for modeling the change in parasite counts over time.

This novel study design was a pragmatic approach to evaluating the possible differential effect of adding artesunate at three doses to CPG−DDS in children and adults. The aim of the trial was to determine the dose of artesunate that would be included in the CDA fixed dose combination for further testing in Phase III studies.

## Methods

### Participants

This study was conducted in accordance with the International Conference on Harmonization Good Clinical Practice Guidelines and all applicable regulatory requirements and the guiding principles of the Declaration of Helsinki. Participants were recruited under a human use protocol approved by and executed in accordance with the ethics committees of the Malawi College of Medicine, the Joint Gambian Government/Medical Research Council and the Liverpool School of Tropical Medicine. The CONSORT checklist and trial protocol for this paper are available as supporting information; see Checklist S1 and Protocol S1.

Eligible participants included adults aged 18−60 years or children aged 12−120 months presenting to a healthcare facility with probable uncomplicated clinical malaria. Inclusion criteria were: weight between 5 and 85 kg; and presence of *P. falciparum* asexual parasitemia, based on microscopic screening of stained blood films at a density of 10,000−100,000 µL^−1^ in adults and 25,000−100,000 µL^−1^ in children. All patients or their parent/guardian provided written or oral witnessed consent and agreed to comply with the requirements of the protocol. A negative pregnancy test was required for all women ≥12 years of age on enrolment.

Subjects were excluded from the study if they had features of severe or complicated falciparum malaria, a known allergy to sulphonamides, evidence of any concomitant infection at the time of presentation (including *P. ovale* and *P. malariae* parasitemia) or any other underlying disease that would compromise the diagnosis and the evaluation of the response to the study medication (including clinical symptoms of immunosuppression, tuberculosis or bacterial infection). Women who had a positive pregnancy test, did not take a pregnancy test or were breastfeeding during the study were also excluded.

Subjects were also excluded if they had received treatment with: a sulphonamide antimalarial or chloroquine within 28 days prior to screening; mefloquine within 21 days prior to screening; amodiaquine, halofantrine, quinine (full course), atovaquone−proguanil, artemisinins, co-artemether, tetracycline or clindamycin within 7 days prior to screening; or treatment for 5 half-lives prior to screening with drugs that have potential antimalarial activity. In addition, subjects could not have used an investigational drug within 30 days or 5 half-lives (whichever was longer) prior to screening, or participated previously in the current study.

### Interventions

A full history and examination was performed at the initial patient assessment. Study participants who fulfilled the inclusion/exclusion criteria were randomized to one of four treatment groups: CPG–DDS alone; CPG−DDS plus 4 mg/kg artesunate; CPG−DDS plus 2 mg/kg artesunate or; CPG−DDS plus 1 mg/kg artesunate. CPG−DDS was provided as Lapdap™ 15/18.75 mg or 80/100 mg tablets (Wulfing Pharma, Gronau, Germany) and patients were given half, one, one and a half or two tablets to achieve a target dose as close as possible to 2 mg/kg CPG and 2.5 mg/kg DDS. Artesunate was provided as 1 mg, 10 mg, 25 mg or 50 mg tablets (Abbott Laboratories, Liestal, Switzerland). All drug treatments were administered according to predefined dosing charts.

All patients received study drug once daily for 3 days (Days 0, 1, 2), during which time they were hospitalized for study-related procedures. Patients were discharged from hospital on Day 3 and asked to return for outpatient follow-up visits on Days 7 and 14.

Blood samples for parasitemia blood slide (10 µL) were obtained from adults via cannula for the first 24 h and by venepuncture thereafter and from children using finger-prick. In adults, sampling was performed on Day 0 at the time of first drug dose then at 1, 2, 3, 4, 6, 8, 12, 18, 24, 30, 36, 42, 48 and 72 h during therapy. In children, blood samples were collected at the time of first dose and at 6, 12, 18, 24, 36, 42, 48 and 72 h during therapy. Samples were also collected from all patients at the follow up visits on Days 7 and 14.

For other outcomes, a 100 µL blood sample was taken at Day 0 at the time of first study dose in adults and children then at 1, 2, 4, 8, 12 and 24 h during therapy in adults and at 12 h only in children. The 12-h sample was the key timepoint used for the parasite viability assessment.

Samples for biochemical and hematological analysis (2 mL of blood), including reticulocyte analysis, were taken on Day 0, Day 3 and Day 7 from all patients and on Day 14 if any value was abnormal on Day 7. Samples were analyzed locally at each investigational center.

### Objectives

The objective of this trial was to determine the most appropriate dose of artesunate to use in combination with CPG–DDS for the treatment of uncomplicated *P. falciparum* malaria.

### Outcomes

The primary endpoint was the mean time to achieve a 90% reduction in parasitemia versus baseline (PC90), determined from Giemsa-stained blood smears. The quality-control procedure for conducting the slide counts involved checking every seventh or tenth slide and eliciting an additional expert reading in the event of significant discrepancies. Screening parasitemia density was calculated using the nominal white blood cell count (WBC) value of 8,000 µL^−1^, according to WHO protocol WHO/HTM/RBM/2003.50 [18]. The actual density of parasitemia was determined by correcting for the WBC for each subject once the hematology results were available. The actual parasitemia value was used as the baseline level for all outcome assessments.

Parasite viability was considered a key secondary endpoint; defined as the percentage of ring-form parasites obtained from the patient 12 h after the first dose of study drug that developed into trophozoites when cultured *in vivo*. It was determined using the method of Murphy, et al. [19]. Briefly, 100 µL venous blood samples were collected 12 h after the first dose of study drug. Parasite development was continued ex-vivo by inoculating blood samples into culture medium and incubating at 37°C for 48 h by which time all viable ring-form parasites should have matured into late trophozoites. Growth was arrested at this phase by inclusion of aphidicolin in the culture medium. The proportion of ring-form parasites and trophozoites on blood slides from the cultured samples was then compared with slides made from the same blood sample immediately after collection.

The mean time to achieve a 99% reduction (PC99) and 50% reduction (PC50) in parasitemia versus baseline were also included as secondary outcomes and calculated using similar methods as mean time to PC90. Slides prepared for parasitology were also read for the presence or absence of gametocytes per 200 white blood cells at baseline and Days 3, 7 and 14.

The number of early or late treatment failures based on the WHO 2002 definitions was also determined [20]. In brief, early treatment failure is a clinical or parasitological failure occurring within 96 h of the start of therapy and late clinical or parasitological failure is defined as occurring after this time and before Day 14. Adequate clinical and parasitological response (ACPR) is the absence of parasitemia on Day 14, irrespective of axillary temperature, without previous treatment failure. Recrudescence was distinguished from new infection using *msp1* and *msp2* polymerase chain reaction genotyping [21]. ACPR was expressed as corrected for these results (ACPRp), to include patients who were late clinical and/or parasitological failures but who had a new infection and no recrudescence of the original infection.

#### Outcome decision matrix

The most appropriate dose of artesunate was determined according to a pre-defined decision matrix, summarized as:

- Choose 2 mg/kg artesunate if all artesunate groups have efficacy greater than CPG–DDS alone and there is no difference in the primary endpoint or any other difference in efficacy or safety between the artesunate treatment groups versus CPG−DDS alone.
- Choose 4 mg/kg artesunate if 1 mg/kg or 2 mg/kg do not have greater efficacy than CPG−DDS alone for the primary endpoint or if there is a difference in the primary endpoint or any other efficacy or safety endpoint between the artesunate 4 mg/kg and the 1 or 2 mg/kg groups versus CPG−DDS alone (in favor of the 4 mg/kg artesunate dose).

In addition, if data from the clinically more vulnerable child group suggested a higher artesunate dose than data from adults, then the higher dose would be chosen. The rationale for the design of the outcome decision matrix can be found in the Discussion section of this paper.

#### Safety

Adverse events and severity (graded as mild, moderate or severe) were determined by the investigators and defined as any untoward medical occurrence temporally associated with the use of study drug, whether or not considered to be related to the study drug. Adverse events were recorded and coded using the latest version of MedDRA. Lack of efficacy was not included as an adverse event *per se* except for the development of clinically severe malaria, including a parasite count of >250,000 µL^−1^ and severe anemia (hemoglobin <5.0 g/dL).

A serious adverse event was defined as one which resulted in death, was life-threatening, resulted in hospitalization or prolongation of hospitalization, was disabling, a congenital anomaly or any other event considered significant by the investigator. The investigator was to follow all serious adverse events until resolution, the condition stabilized, the event was otherwise explained or the subject was lost to follow-up.

### Sample size

A sample size of eighty-eight evaluable subjects (22 per treatment group) would provide 90% power at a 5% two-sided significance level to detect a difference of 9 h in the mean time to PC90 between the groups treated with CPG−DDS plus 4, 2, or 1 mg/kg of artesunate versus CPG–DDS alone. Allowing for a 25% drop-out rate, a target sample size of 120 adults and 120 children was calculated.

The adult group was stratified by gender to prevent a bias. The child group was stratified by age to ensure that both younger and older children were represented.

### Randomization−Sequence generation

The randomization code was generated by computer at GlaxoSmithKline, Greenford, UK. Different codes were allocated for adult sex strata and child age strata and distributed using scratch cards bearing unique study numbers.

### Randomization−Allocation concealment

This was an open-label study.

### Randomization−Implementation

Scratch cards were provided to clinicians and were selected consecutively on patient enrolment to reveal treatment allocation.

### Blinding

In order to achieve accurate artesunate dosing, this study was conducted open label to the treating physician and nursing staff. However, the technicians performing the slide reading and parasite viability analysis were blinded to the treatment group. Subjects assigned to receive CPG−DDS alone did not receive placebo to replace artesunate.

### Statistical methods

The intent to treat (ITT) population included all randomized subjects who received at least one dose of study medication. The per-protocol (PP) population was a sub-set of the ITT population including those patients who were compliant with all doses of study medication, had no major protocol violations, took no prohibited concomitant medications during the treatment period and had a baseline actual parasitemia level of ≥5,000 µL^−1^ for adults and ≥12,500 µL^−1^ for children.

The primary analysis was conducted on the Day 3 PP population who had completed all visits through to the assessment at Day 3. Additional sensitivity analyses were prepared using the ITT population and the Day 14 PP population; a sub-set of the Day 3 PP population including patients that completed all visits during the study.

The null hypothesis was that there would be no difference in the primary endpoint (mean time to PC90) between CPG−DDS alone and CPG−DDS plus artesunate at each of the three doses.

The treatment difference was calculated from the mean values of time to PC90. The principal analysis of mean time to PC90 was performed for the subjects with confirmed 90% reduction of parasitemia by 72 h based on a logistic model. This approach was taken as it was not feasible to draw blood samples more frequently and yet it was desirable to try to determine the time to PC90 that likely occurs between sampling time points. For each subject, observed parasite count data from 0−72 h were log-transformed. A logistic model was used to estimate the change in parasite count between observations. The logistic curve had the following form: y = α+λ/(1+e^−β(x−μ)^) where α = the lower asymptote, α+λ = the upper asymptote, β = the parasitemia reduction rate, µ = the time of maximum rate of reduction (i.e. point of inflexion), x = time from first dose (in hours); y = log[1+P_(time = x)_] and (P = parasite count), hence P = e^y^–1. The model of best fit was selected for each subject by two independent statisticians blinded to treatment group based on a set of pre-defined evaluation criteria: possible data errors; outliers affecting model fit and whether to exclude them; estimated modeled baseline and fit of model in baseline region; estimate of PC90 and fit in these regions; and closeness of model fit with observed data. Any subjects where the logistic curve fitted to derive time to PC90 was not reliable were omitted in order to preserve the integrity of the analysis, termed ‘poor model fit’. Poor model fit was categorized as: the model being inadequate to fit to the data; there being insufficient data to fit to the model; or the subject not achieving PC90 by 72 h based on the model. The mean time to PC90 was determined from the derived times to PC90s of the available subjects.

In addition to the logistic model-based approach, the time to PC90 based strictly on the observed parasite counts was conducted as a sensitivity analysis. The aim of the sensitivity analysis was to validate and reinforce the principal analysis as described above.

Comparisons were made across treatment using analyses of covariance weighted according to stratification by gender and center in adult subjects and separately in children stratified by age group at baseline: 1−<2, 2−<3, 3−<4, 4−<5 or 5−<10 years (120 months) (children were enrolled at one center only).

No adjustment for the multiple comparisons to the CPG–DDS alone arm was made. However, in order to preserve the overall significance level at 5%, a step-down closed testing procedure was used whereby the 4 mg/kg artesunate group was compared to CPG−DDS alone and only if there was a statistically significant difference at the 5% level was the 2 mg/kg compared with CPG−DDS alone; only if this showed a significant difference was the 1 mg/kg dose compared with CPG−DDS alone.

The study was powered for the primary analysis. Additional analyses were presented for descriptive purposes, and no further adjustments for multiplicity have been implemented. Summary statistics (mean, standard deviation, median, minimum, maximum) for parasite counts were tabulated by treatment group for adults and children. The proportion of subjects with gametocytes at each timepoint was also summarized along with 95% confidence intervals. Analyses of the secondary endpoints of mean time to PC50 and mean time to PC99 were conducted in a similar manner to those performed for PC90.

The ITT population was used for safety analyses. Safety analyses included summaries of the proportion of patients with adverse events and serious adverse events by treatment group. For laboratory parameters, summary statistics (mean, standard deviation, median, minimum and maximum) for each timepoint were presented by treatment group. In addition, the proportion of subjects with decreases in hemoglobin (≥2 g/dL and ≥4 g/dL) was tabulated by treatment group.

## Results

### Participant flow

**Figure 1:**
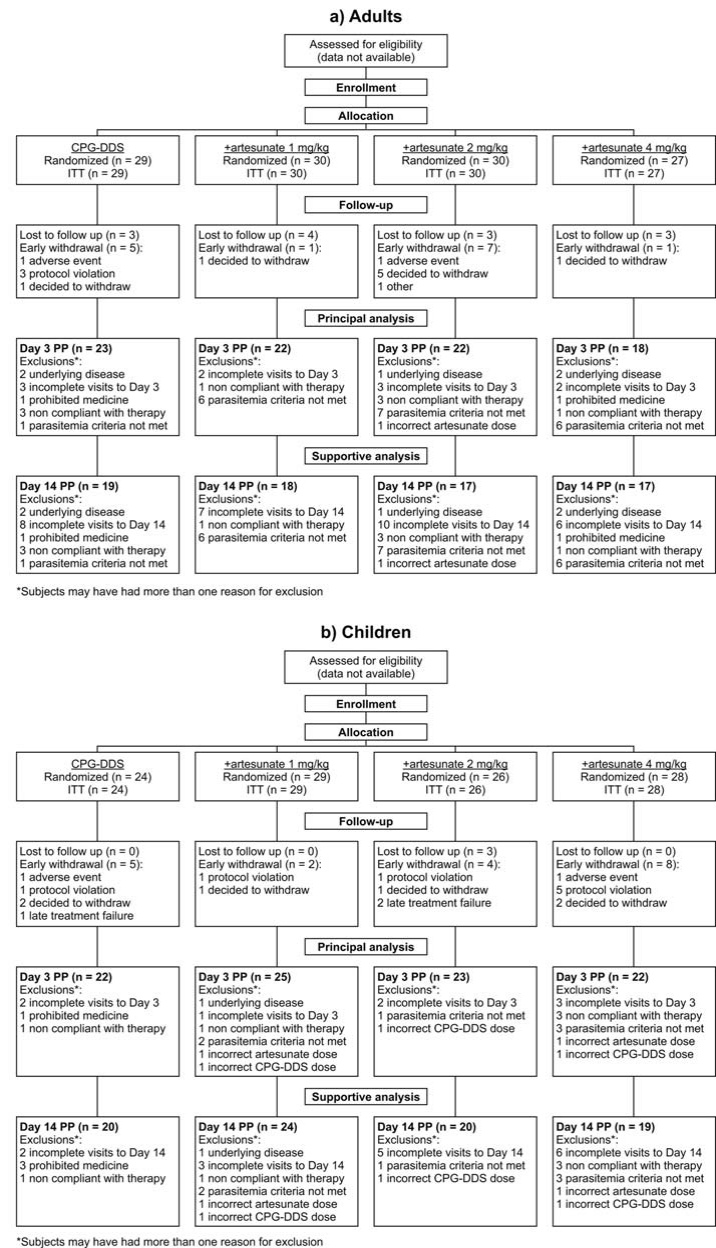
Trial profile of the study and patient flow for a) adults and b) children. ITT = intent to treat population; PP = per-protocol population.

Participant flow for the adult and child groups is shown in Figure 1. No data are available regarding the precise number of patients screened for inclusion in this study, or the reasons for their not being enrolled.

There were 116 adults enrolled in the study. The Malawi centers contributed 62 patients; 14 were randomized to CPG−DDS alone, and 17, 17 and 14 to +artesunate 1, 2 or 4 mg/kg, respectively. The Gambia center contributed 54 adult patients, 15 randomized to CPG−DDS alone and 13 patients each randomized to +artesunate 1, 2 or 4 mg/kg. All of the 107 children enrolled into the study were recruited from the Malawi center.

All randomized subjects were included in the ITT population. Reasons for withdrawal from the study are shown in Figure 1; there were no apparent differences between the study arms for adults or children.

### Recruitment

The trial was conducted between June 2002 and October 2004 at the Queen Elizabeth Central Hospital, Blantyre and six local health centers in Malawi and between September 2004 and February 2005 at the AFPRC General Hospital, Farafenni, The Gambia.

### Baseline data

The baseline demographic and clinical characteristics of the Day 3 PP population are shown in Table 1 and were very similar to those of the ITT population.

**Table 1:** Baseline demographic and clinical characteristics of the Day 3 per-protocol population.

Characteristic	CPG–DDS	+artesunate 1 mg/kg	+artesunate 2 mg/kg	+artesunate 4 mg/kg
***Adults***	N = 23	N = 22	N = 22	N = 18
Median age, years (range)	27.0 (18−55)	25.0 (18−50)	27.0 (21−59)	28.0 (18−58)
Male sex, n (%)	12 (52.2)	12 (54.5)	13 (59.1)	12 (66.7)
Median weight, kg (range)	53.8 (44.5−66.0)	56.3 (42.0−81.0)	53.5 (44.0−76.0)	54.9 (42.5−76.0)
Median pre-dose parasitemia, µL^−1^ (inter-quartile range)	26912 (14854:44120)	16279 (10451:35707)	22828 (15366:30121)	22527 (13537:49021)
***Children***	N = 22	N = 25	N = 23	N = 22
Median age, months (range)	29.0 (14−112)	46.0 (13−116)	36.0 (13−118)	40.5 (15−121)
Male sex, n (%)	13 (59.1)	13 (52.0)	17 (73.9)	12 (54.5)
Median weight, kg (range)	10.9 (7.9−29.8)	13.4 (7.4−25.6)	12.8 (8.2−35.8)	12.4 (7.3−26.2)
Median pre-dose parasitemia, µL^−1^ (inter-quartile range)	44973 (34596:56027)	37730 (30757:50656)	45880 (30800:59307)	39972 (24480:56022)

NB: All patients were black African.

For adults, the ratio of females to males was similar in all treatment groups except the CPG–DDS +artesunate 4 mg/kg group, where the ratio was 6:12. These results were consistent with the 2:3 adult female:male randomization (Table 1).

For children, the median age in the CPG–DDS +artesunate 1 mg/kg group was 46 months, compared with 29−41 months in the other groups. The ratio of females to males was similar in all treatment groups except the CPG–DDS +artesunate 2 mg/kg group, where the ratio was 6∶17 (Table 1).

### Numbers analyzed

The number of patients analyzed in the Day 3 PP population and the Day 14 PP population are shown in Figure 1. Across the treatment groups 67−79% of adult subjects completed all study assessments to Day 3 and were included in the Day 3 PP population and 57−66% qualified for the Day 14 PP population. Across the treatment groups, a higher proportion of children were included in the Day 3 PP population (79−92%) and Day 14 PP population (68−83%) compared to adults. Similar numbers of patients were excluded from these analyses for each dose group; reasons for exclusion are shown in Figure 1.

In addition, six patients were excluded from the principal analysis due to ‘poor model fit’. These exclusions were decided by statisticians blind to treatment group. Two adult patients (one in the CPG−DDS and one in the +artesunate 2 mg/kg group) and four children (two in the CPG−DDS, one in the +artesunate 1 mg/kg and one in the +artesunate 2 mg/kg group) were excluded as the model was inadequate to fit their data. No patients were excluded due to not achieving PC90 by 72 h. Patients excluded from the principal analysis were included in the sensitivity analysis.

### Outcomes and estimation

#### Primary outcome: Mean time to PC90

**Figure 2:**
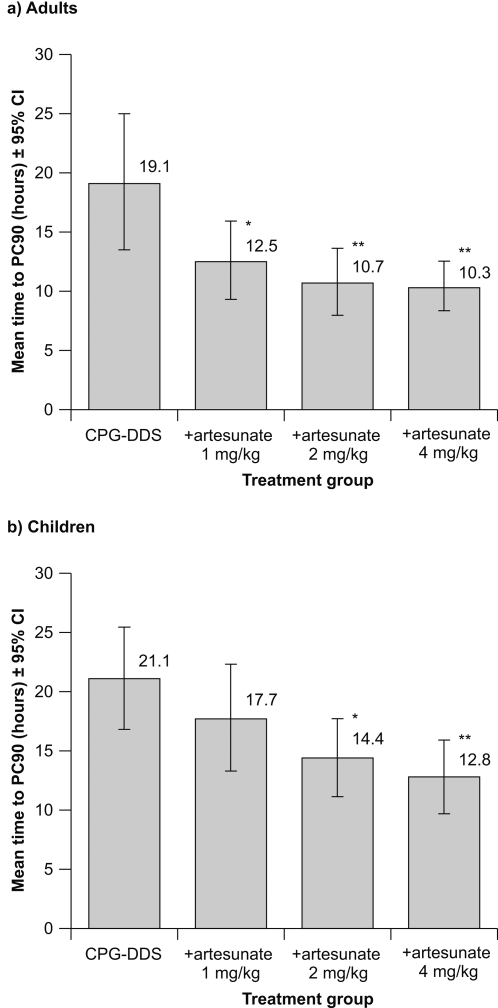
Primary efficacy outcome: mean time to PC90 (±95% confidence intervals) in the Day 3 per-protocol population for (a) adults and (b) children. *P ≤0.05 versus CPG−DDS alone; **P ≤0.01 versus CPG–DDS alone.

For adults, in the Day 3 PP population, the mean time to PC90 was 19.1 h with CPG−DDS alone. Statistical analysis indicated a significant decrease in mean time to PC90 between CPG–DDS alone and the +artesunate 4 mg/kg (−8.7 h; *P* = 0.002), 2 mg/kg (−8.4 h; *P* = 0.002) and 1 mg/kg (−6.6 h; *P* = 0.013) groups (Figure 2, Table 2).

**Table 2:** Mean time to PC90 (primary endpoint) for the Day 3 per-protocol (PP) population (principal analysis population) and supportive analyses: sensitivity analysis on the Day 3 PP population and outcomes for the Day 14 PP population and the intent to treat (ITT) population.

Analysis	Adults				Children			
	CPG–DDS	+artesunate 1 mg/kg	+artesunate 2 mg/kg	+artesunate 4 mg/kg	CPG–DDS	+artesunate 1 mg/kg	+artesunate 2 mg/kg	+artesunate 4 mg/kg
***Day 3 PP population*** a	n = 22	n = 22	n = 21	n = 18	n = 20	n = 24	n = 22	n = 22
Mean time to PC90, h±SD	19.1±12.8	12.5±7.6	10.7±6.2	10.3±4.2	21.1±9.1	17.7±10.6	14.4±7.2	12.8±7.1
Treatment difference (95% CI)	−	−6.6 (−11.8, −1.5)	−8.4 (−13.6, −3.2)	−8.7 (−14.1, −3.2)	−	−3.3 (−8.6, 2.0)	−6.4 (−11.7, −1.0)	−7.4 (−12.9, −1.8)
*P* value	−	*0.013*	*0.002*	*0.002*	−	0.222	*0.020*	*0.010*
***Sensitivity analysis***	n = 23	n = 22	n = 22	n = 18	n = 22	n = 25	n = 23	n = 22
Mean time to PC90, h±SD	20.2±17.1	13.9±8.8	11.8±6.0	11.9±5.0	26.3±14.9	20.8±12.0	15.2±8.3	15.1±7.4
Treatment difference (95% CI)	−	−6.2 (−12.6, 0.2)	−8.5 (−14.9, −2.1)	−8.1 (−14.9, −1.3)	−	−5.0 (−11.7, 1.6)	−11.0 (−17.4, −4.0)	−10.0 (−17.0, −3.1)
*P* value	−	0.057	*0.010*	*0.020*	−	0.134	*0.002*	*0.005*
***Day 14 PP population***	n = 18	n = 18	n = 17	n = 17	n = 18	n = 23	n = 19	n = 19
Mean time to PC90, h±SD	20.0±12.9	12.9±6.8	10.9±6.4	10.1±4.2	21.4±9.5	18.5±10.2	14.2±7.8	13.1±6.3
Treatment difference (95% CI)	−	−7.3 (−12.9, −1.7)	−8.9 (−14.6, −3.2)	−9.8 (−15.5, −4.1)	−	−2.8 (−8.3, 2.8)	−6.6 (−12.3, −0.9)	−7.4 (−13.3, −1.5)
*P* value	−	*0.012*	*0.003*	*0.001*	−	0.322	*0.023*	*0.015*
***ITT population***	n = 26	n = 29	n = 27	n = 26	n = 21	n = 28	n = 24	n = 25
Mean time to PC90, h±SD	19.7±12.6	12.0±7.3	10.1±5.9	9.5±4.1	21.4±9.0	16.7±10.4	14.6±7.0	12.3±7.2
Treatment difference (95% CI)	−	−7.7 (−12.1, −3.3)	−9.5 (−14.0, −5.1)	−10.2 (−14.7, −5.8)	−	−4.4 (−9.3, 0.5)	−6.0 (−11.1, −0.9)	−7.9 (−13.1, −2.7)
*P* value	−	*0.0007*	*<0.0001*	*<0.0001*	−	0.078	*0.022*	*0.003*

a n = Day 3 PP population minus six patients (two adults, four children) with ‘poor model fit’ due to the model being inadequate to fit their data.

*P* values that are statistically significant are in italics to aid comparison.

For children, in the Day 3 PP population, the mean time to PC90 was 21.1 h with CPG−DDS alone. There was a significant decrease in the mean time to PC90 versus CPG−DDS alone for the +artesunate 4 mg/kg (−7.4 h; *P* = 0.010) and the 2 mg/kg (−6.4 h; *P* = 0.020) groups (Figure 2, Table 2). The reduction in mean time to PC90 with 1 mg/kg artesunate of −3.3 h versus CPG−DDS alone was not statistically significant (*P* = 0.222) (Table 2).

A sensitivity analysis was performed for the primary endpoint based on observed parasite count data, including subjects who had a poor model fit or insufficient data. As expected, mean values of time to PC90 based on observed timepoints were higher than in the principal analysis using the model-based approach (Table 2). Outcomes were similar to the principal analysis except that in the sensitivity analysis mean time to PC90 for adults receiving CPG−DDS +artesunate 1 mg/kg was not significantly different from CPG−DDS alone (treatment difference −6.2 h; *P* = 0.057) (Table 2). An analysis of outcomes for mean time to PC90 for the Day 14 PP and ITT populations was consistent with those of the Day 3 PP analysis (Table 2).

#### Secondary outcomes: Parasite viability

In adults at 12 h after the first dose of study drug, mean parasite viability was 12.4% in the CPG−DDS group. There was a significant decrease in viability observed for the +artesunate 4 mg/kg (−12.4%; *P* = 0.024) and 2 mg/kg (−12.2%; *P* = 0.011) groups versus CPG–DSS alone (Table 3). However, there was no significant difference for the +artesunate 1 mg/kg group versus CPG−DDS alone (−10.2%; *P* = 0.104).

**Table 3:** Ex-vivo parasite viability at 12 h after the first dose of study drug in the Day 3 per-protocol population.

Parasite viability	CPG–DDS	+artesunate 1 mg/kg	+artesunate 2 mg/kg	+artesunate 4 mg/kg
***Adults*** a	n = 14	n = 6	n = 16	n = 9
Mean viability±SD, % (range)	12.4±21.9 (0−59)	1.2±2.9 (0−7)	0 (0−0)	0 (0−0)
Treatment difference (95% CI)	−	−10.2 (−22.6, 2.2)	−12.2 (−21.3, −3.0)	−12.4 (−23.1, −1.7)
*P* value	−	0.1036	0.0106	0.0239
***Children*** a	n = 19	n = 19	n = 21	n = 20
Mean viability±SD, % (range)	71.2±104.0 (0−416)	13.5±31.7 (0−126)	1.5±5.3 (0−23)	0.2±0.7 (0−3)
Treatment difference (95% CI)	−	−57.3 (−93.1, −21.6)	−68.1 (−103.2, −32.9)	−68.6 (−105.1, −32.1)
*P* value	−	0.0021	0.0002	0.0004

a n = Day 3 per-protocol population minus patients excluded due to lack of a valid parasite viability assessment at 12 h±1 h.

In children, the mean parasite viability with CPG−DDS alone was 71.2% at 12 h. All artesunate combinations significantly reduced the number of viable parasites at 12 h versus CPG–DDS alone: by −68.6% (*P* = 0.0004), −68.1% (*P* = 0.0002) and −57.3% (*P* = 0.0021) for the +artesunate 4, 2, and 1 mg/kg groups, respectively (Table 3).

#### Secondary outcomes: Mean time to PC50 and PC99

**Figure 3:**
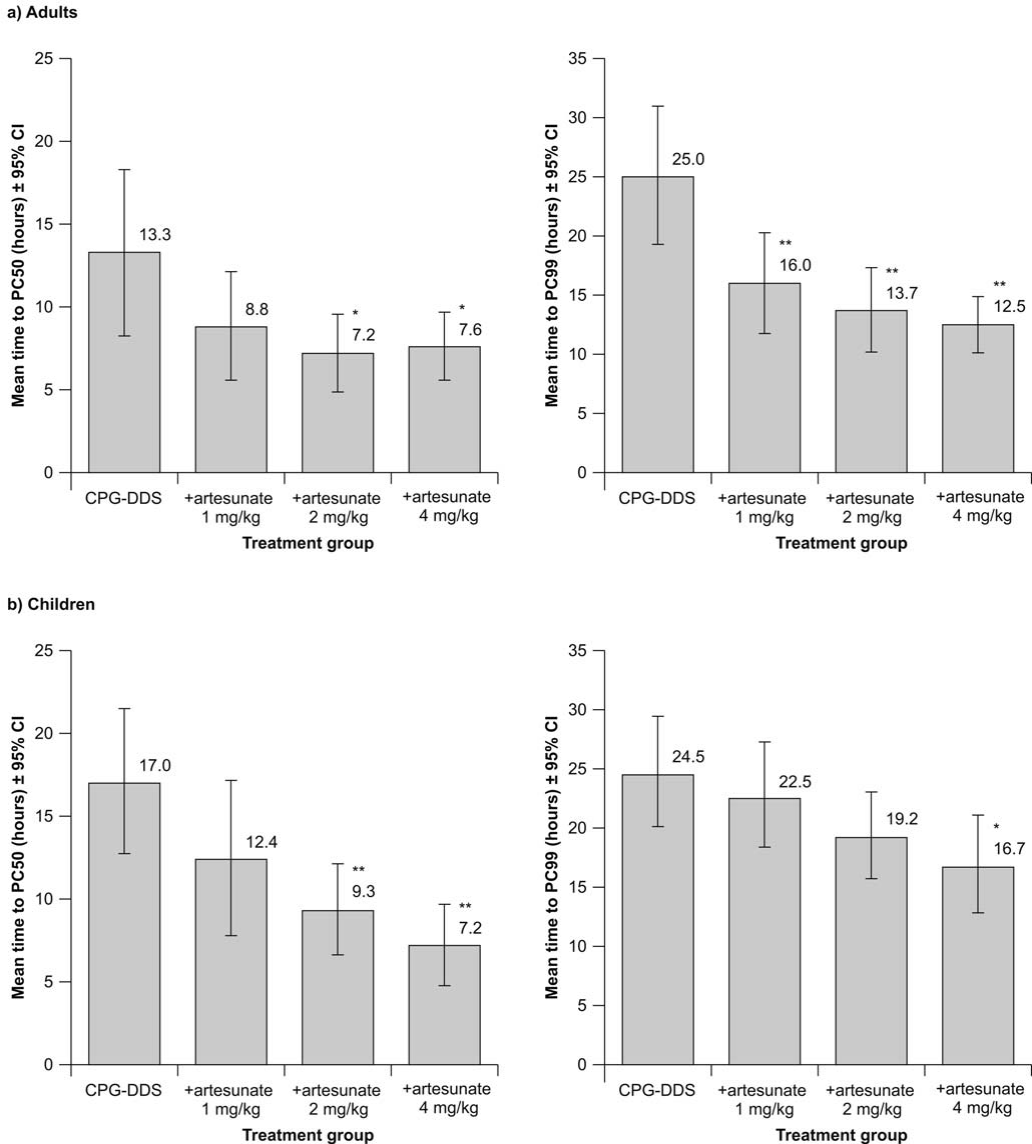
Results for Day 3 per-protocol population for mean time to PC50 and mean time to PC99 for (a) adults and (b) children. *P ≤0.05 versus CPG−DDS alone; **P ≤0.01 versus CPG–DDS alone. Five adults (two in the CPG−DDS group, two in the +artesunate 1 mg/kg group and one in the +artesunate 2 mg/kg group) and 14 children (three from the CPG−DDS group, and five, three and three from the +artesunate 1, 2 and 4 mg/kg groups, respectively) were excluded from the mean time to PC50 analysis due to poor model fit. Seven adults (five in the CPG−DDS group, and one each in the +artesunate 1 mg/kg and 2 mg/kg groups) and five children (two from the CPG−DDS group, two from the +artesunate 1 mg/kg and one from the +artesunate 2 mg/kg group) were excluded from the mean time to PC99 analysis due to poor model fit.

In adults, for the Day 3 per-protocol population, the mean time to PC50 for CPG−DDS alone was 13.3 h (SD±10.8 h). Statistical analysis showed that in adults, addition of artesunate 4 mg/kg or 2 mg/kg resulted in a significant reduction in mean time to PC50 versus CPG−DDS alone; treatment difference −5.4 h (*P* = 0.025) and −5.9 h (*P* = 0.012), respectively (Figure 3a). There was no significant effect of +artesunate 1 mg/kg versus CPG−DDS alone (treatment difference −4.5 h; *P* = 0.054).

In children, the mean time to PC50 was 17.0 h (SD±9.0) for CPG−DDS alone. As for adults, in children the +artesunate 4 mg/kg and +artesunate 2 mg/kg groups had a significantly shorter mean time to PC50 than CPG−DDS alone; treatment difference −9.0 h (*P* = 0.001) and −7.4 h (*P* = 0.005), respectively (Figure 3b). There was no significant effect on mean time to PC50 of adding 1 mg/kg artesunate versus CPG−DDS alone in either adults or children (treatment difference −4.5 h; *P* = 0.089).

The mean time to PC99 in adults with CPG−DDS alone was 25.0 h (SD±11.5) (Figure 3a). There was a significant reduction in mean time to PC99 with +artesunate 4 mg/kg (−12.8 h; *P* = <0.001), 2 mg/kg (−11.4 h; *P* = <0.001) and 1 mg/kg (−9.1 h; *P* = 0.002) versus CPG−DDS alone.

The mean time to PC99 in children with CPG−DDS alone was 24.5 h (SD±9.8) (Figure 3b). In contrast to adults, in children a significant decrease in mean time to PC99 versus CPG−DDS alone was observed only for the +artesunate 4 mg/kg group (−9.0 h; *P* = 0.018).

#### Secondary outcomes: Gametocyte prevalence

**Figure 4:**
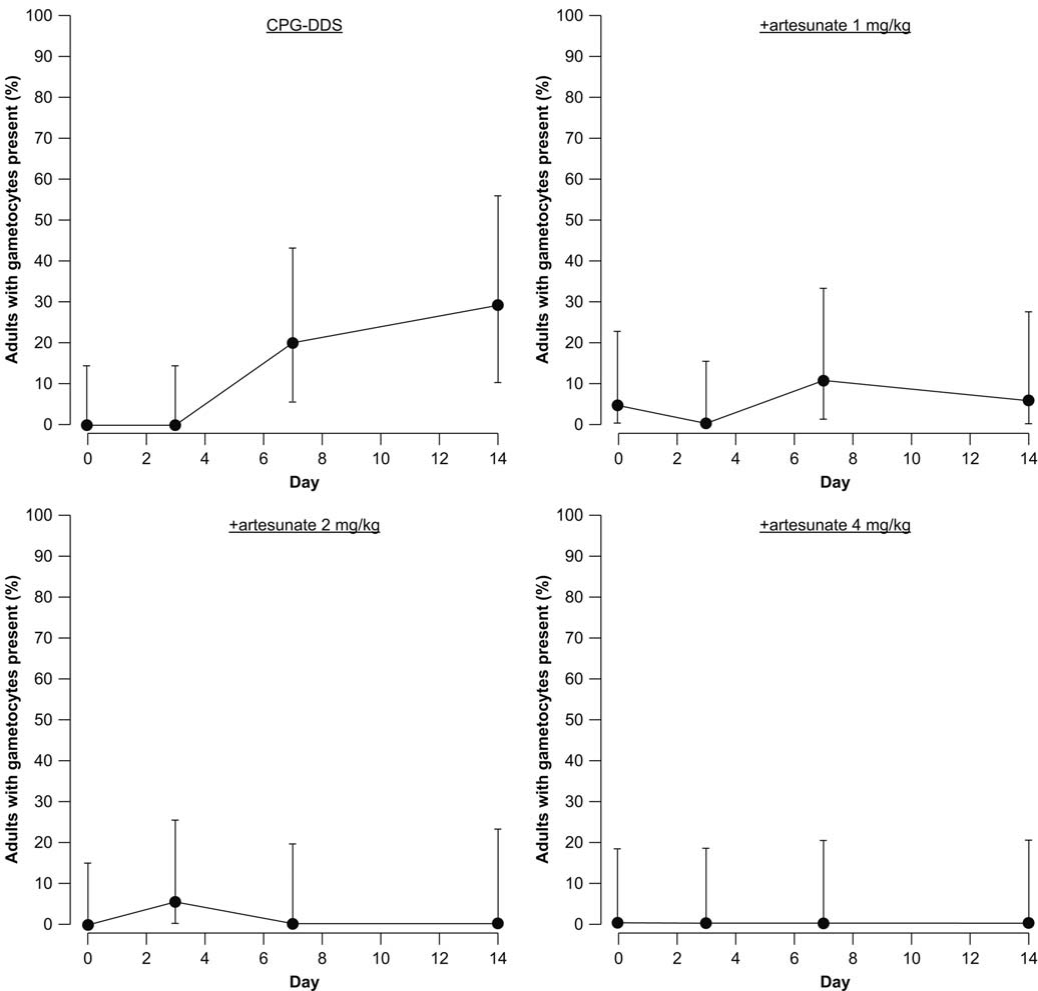
Adults: Percentage of patients who were gametocytemic in the Day 3 per-protocol population at each scheduled assessment time.

In adults, the percentage of gametocytemic patients in the CPG–DSS alone group increased during the study to a maximum of 29.4% of patients assessed at Day 14 (Figure 4). In the +artesunate 1 mg/kg group, the proportion of patients with gametocytes was increased versus Day 0 (4.5%) at both Day 7 (10.5%) and Day 14 (5.6%). For the +artesunate 2 mg/kg group, there was small increase in patients with gametocytes at Day 3 (5.0%) versus Day 0 (0%), though gametocytes were absent at Days 7 and 14. The +artesunate 4 mg/kg dose completely suppressed gametocytes throughout the study.

**Figure 5:**
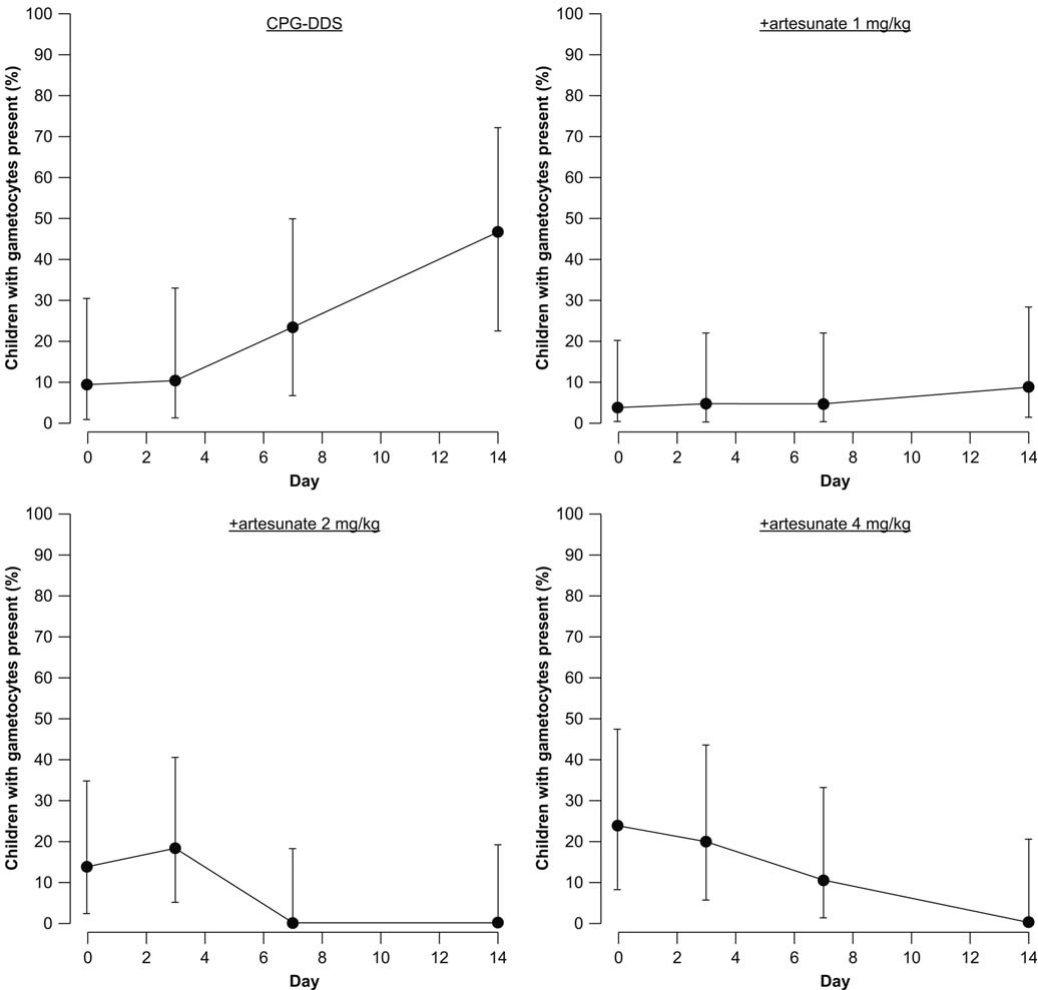
Children: Percentage of patients who were gametocytemic in the Day 3 per-protocol population at each scheduled assessment time.

In children, the percentage of gametocytemic patients in the CPG–DDS group increased during the study to a maximum of 47.1% at Day 14 (Figure 5). In the +artesunate 1 mg/kg group, the proportion of patients with gametocytes increased throughout the study to a maximum of 8.7% at Day 14. In the +artesunate 2 mg/kg group, gametocytes were present in 18.2% of patients at Day 3 versus 13.6% at Day 0, though no gametocytes were detected at Day 7 or 14. In the +artesunate 4 mg/kg group, despite a high Day 0 proportion of gametocytemic patients (23.8%), gametocyte carriage was reduced at each sampling point, achieving 0% at Day 14.

#### Secondary outcomes: Treatment failures

The trial was underpowered for statistical analysis of clinical endpoints. Using the modified WHO 2002 definitions [20], based on the Day 14 PP population there were only three failures: one adult and two children−all received CPG–DDS alone (Table 4). At 14 days, the ACPR and ACPRp were identical as all failures were recrudescences, though one child had a recrudescence plus a new infection. A further child in the CPG−DDS +artesunate 4 mg/kg group had an extra visit recorded with recrudescence plus new infection at Day 21.

**Table 4:** Treatment responses at Day 14 based on WHO 2002 definitions [20] (Day 14 per-protocol population).

Treatment response, n (%)	CPG–DDS	+artesunate 1 mg/kg	+artesunate 2 mg/kg	+artesunate 4 mg/kg
***Adults***	N = 19	N = 18	N = 17	N = 17
Early treatment failure	0	0	0	0
Late clinical failure	0	0	0	0
Late parasitological failure	1 (5.3)	0	0	0
ACPRp [95% CI]	18 (94.7) [74.0, 99.9]	18 (100) [81.5, 100]	17 (100) [80.5, 100]	17 (100) [80.5, 100]
***Children***	N = 20	N = 24	N = 20	N = 19
Early treatment failure	0	0	0	0
Late clinical failure	2 (10.0)	0	0	0
Late parasitological failure	0	0	0	0
ACPRp [95% CI]	18 (90.0) [68.3, 98.8]	24 (100) [85.8, 100]	20 (100) [83.2, 100]	19 (100) [82.4, 100]

ACPRp = adequate clinical and parasitological response corrected for polymerase chain reaction genotyping, i.e. including patients ACPR at Day 14 plus those who were late clinical and/or parasitological failures but with a new infection and no recrudescence.

Owing to the wide confidence intervals, there were no clear differences in treatment failures observed between the artesunate dose groups and CPG–DDS alone in either adults or children or between adults and children (Table 4).

### Ancillary analyses

#### Compliance with medication

The number of patients in the ITT population in whom the initial dose or re-dose of study drug(s) was successful on all 3 days (administration compliance) was 94.2% (210/223). For adults, administration compliance was 89.7%, 96.7%, 90.0%, 96.3% for the CPG−DDS only, and +artesunate 1, 2 and 4 mg/kg groups, respectively; corresponding numbers for children were 95.8%, 96.6%, 100% and 89.3%, respectively.

Overall, in the ITT population, 91.9% (205/223) of subjects were totally compliant, i.e. the initial dose or re-dose of study drug(s) was successful on all 3 days and they received the correct mg/kg/day target doses of study drug as per the randomization schedule. For adults, 89.7%, 96.7%, 86.7% and 96.3% of patients were totally compliant in the CPG−DDS only, and +artesunate 1, 2 and 4 mg/kg groups, respectively; corresponding numbers for children were 95.8%, 93.1%, 96.2% and 82.1%, respectively.

### Adverse events

This study was not powered to conduct formal comparisons of safety between treatment arms. The observed nature and incidence of adverse events were similar between the groups (Table 5) and were consistent with the known profile of CPG−DDS. The most common adverse events in adults were nausea, reticulocytosis and acquired methemoglobinemia and in children were reticulocytosis, anemia and acquired methemoglobinemia (Table 5).

**Table 5:** Treatment emergent investigational drug-related adverse events reported during the study and the most common adverse events reported in ≥10% of patients in any treatment group for the intent to treat (safety) population.

Preferred term, n (%)	Adults				Children			
	CPG–DDS	+artesunate 1 mg/kg	+artesunate 2 mg/kg	+artesunate 4 mg/kg	CPG–DDS	+artesunate 1 mg/kg	+artesunate 2 mg/kg	+artesunate 4 mg/kg
	N = 29	N = 30	N = 30	N = 27	N = 24	N = 29	N = 26	N = 28
Any adverse event	10 (34.5)	10 (33.3)	11 (36.7)	10 (37.0)	19 (79.2)	19 (65.5)	21 (80.8)	16 (57.1)
Reticulocytosis	2 (6.9)	3 (10.0)	3 (10.0)	1 (3.7)	7 (29.2)	11 (37.9)	9 (34.6)	7 (25.0)
AM	4 (13.8)	0	2 (6.7)	3 (11.1)	3 (12.5)	3 (10.3)	2 (7.7)	5 (17.9)
Anemia	0	1 (3.3)	0	2 (7.4)	6 (25.0)	1 (3.4)	6 (23.1)	6 (21.4)
Vomiting	4 (13.8)	2 (6.7)	2 (6.7)	0	3 (12.5)	2 (6.9)	3 (11.5)	1 (3.6)
Abdominal pain	3 (10.3)	1 (3.3)	2 (6.7)	0	4 (16.7)	0	2 (7.7)	3 (10.7)
Nausea	4 (13.8)	4 (13.3)	3 (10.0)	2 (7.4)	1 (4.2)	0	0	1 (3.6)
Anorexia	0	2 (6.7)	2 (6.7)	1 (3.7)	3 (12.5)	2 (6.9)	4 (15.4)	0
Cough	0	1 (3.3)	1 (3.3)	2 (7.4)	4 (16.7)	3 (10.3)	2 (7.7)	1 (3.6)
Thrombocythemia	0	0	0	0	1 (4.2)	3 (10.3)	0	1 (3.6)
AST increased	1 (3.4)	1 (3.3)	0	1 (3.7)	1 (4.2)	2 (6.9)	1 (3.8)	5 (17.9)
Diarrhea	1 (3.4)	1 (3.3)	1 (3.3)	0	2 (8.3)	3 (10.3)	1 (3.8)	2 (7.1)
Thrombocytopenia	0	0	0	0	1 (4.2)	5 (17.2)	4 (15.4)	3 (10.7)
Leukocytosis	0	0	0	0	1 (4.2)	0	3 (11.5)	0

AM, acquired methemoglobinemia; AST, aspartate aminotransferase.

There were some differences in the reporting of adverse events by center (for adults). In Malawi, adverse events were recorded for 11/14 (78.6%) of adults receiving CPG−DDS alone and 11/17 (64.7%), 12/17 (70.6%) and 11/14 (78.6%) for those receiving 1, 2 or 4 mg/kg of artesunate respectively. In The Gambia, the number of adverse events reported was much lower than in Malawi: 7/15 (46.6%) patients for CPG−DDS alone and 5/13 (38.5%), 3/13 (23.1%) and 4/13 (30.7%) for those receiving 1, 2 or 4 mg/kg of artesunate respectively. However, in both centers there were no apparent differences in the nature or incidence of adverse events between the CPG−DDS alone group and the artesunate groups.

Two non-fatal treatment-emergent serious adverse events were observed in two adults (one each in the +artesunate 4 and 2 mg/kg groups) and six were observed in three children (two in the CPG−DDS group and one in the +artesunate 4 mg/kg group). In addition, one child experienced vomiting before and after dosing with study treatment and required an additional night in hospital. Six of the serious adverse events were not thought to be related to study medication and included one case of sepsis in an adult patient and in the child group two cases of sepsis and one each of malnutrition, malaria and cerebral malaria.

Two of the serious adverse events were thought to be related to study drug. One adult case of severe hemolytic anemia in the +artesunate 2 mg/kg group presented at Day 9 with a hemoglobin concentration of 6.6 g/dL (baseline 15.4 g/dL), was treated with two units of blood and the patient recovered. However, this patient subsequently went on to experience anaphylactic shock (day 14 after recruitment) which resulted in death. The investigator considered this event to be related to herbal preparations that the subject had taken and not to study medication.

The second drug-related event was a report of anemia in a 10 year old child in the +artesunate 4 mg/kg group who presented with a hemoglobin concentration of 3.8 g/dL at Day 7 (baseline 12.0 g/dL), was treated with blood transfusion and antibiotics and recovered.

No artesunate dose-related changes in laboratory blood parameters were noted (Table 6). There was a progressive decrease in hemoglobin concentration through Day 3, 7 and 14 in adults (Table 6). However, adult reticulocyte counts on Day 7 and 14 were increasing, suggesting that if patients had been followed up to Day 28, hemoglobin concentration may have corrected to baseline (Table 6). At Day 7, 45.2% (42/93) of all adults had a decrease in hemoglobin concentration from baseline of ≥2 g/dL and 17.2% (16/93) had a decrease of ≥4 g/dL (Table 7). Day 14 assessments were only measured if clinically indicated. Of the patients with assessments at Day 14, 38.1% (16/42) had ≥2 g/dL reduction in hemoglobin concentration from baseline, though the proportion with a decrease of ≥4 g/dL had fallen to 7.1% (3/42) (Table 7).

**Table 6:** Blood parameters where significant changes were observed during treatment for the intent to treat (safety) population.

Parameter	Time point	CPG–DDS		+artesunate 1 mg/kg		+artesunate 2 mg/kg		+artesunate 4 mg/kg	
		n	Mean±SD	n	Mean±SD	n	Mean±SD	n	Mean±SD
***Adults***									
Hemoglobin (g/dL)	Day 0	29	13.73±2.16	30	13.49±2.62	30	12.71±2.41	27	13.34±2.26
	Day 3	25	12.54±2.95	28	12.20±2.64	25	12.22±3.24	26	12.07±2.22
	Day 7	22	11.44±2.68	27	11.61±2.76	22	11.41±2.49	22	11.36±2.32
	Day 14^a^	11	11.32±2.39	13	10.79±1.44	10	10.57±1.80	8	9.58±1.29
Reticulocyte count (%)	Day 0	28	1.47±1.78	29	1.30±1.45	29	1.45±1.91	27	1.42±1.47
	Day 3	25	1.29±1.57	25	1.55±1.82	23	1.00±1.11	25	0.85±0.92
	Day 7	29	1.84±2.80	24	2.70±4.43	21	1.84±1.53	21	2.78±4.35
	Day 14^a^	11	4.88±3.25	12	3.12±2.21	9	2.65±0.69	7	4.11±0.58
Methemoglobin (%)	Day 0	19	0.46±0.45	20	0.48±0.35	22	0.38±0.26	21	0.44±0.37
	Day 3	16	4.75±5.02	17	2.61±1.82	20	4.04±4.49	19	4.76±3.05
	Day 7	15	1.07±1.34	20	1.23±0.82	16	0.95±1.13	19	0.74±0.37
	Day 14^a^	6	0.77±0.38	9	0.57±0.30	4	0.25±0.25	4	0.43±0.34
Platelets (×10^9^/L)	Day 0	28	106.3±52.9	30	101.8±50.5	30	94.6±47.5	27	109.8±61.5
	Day 3	25	113.2±43.4	27	163.4±67.2	25	149.1±42.7	26	141.3±63.4
	Day 7	22	176.5±79.6	27	227.3±79.5	22	232.4±66.4	22	232.5±99.9
	Day 14^a^	11	249.0±105.8	12	257.2±122.3	10	253.1±74.0	7	218.6±84.0
***Children***									
Hemoglobin (g/dL)	Day 0	24	9.03±1.76	29	9.12±1.48	25	9.13±1.81	27	8.89±1.42
	Day 3	23	7.97±2.01	27	8.54±1.50	23	8.16±1.66	23	8.23±1.68
	Day 7	23	8.48±1.51	27	8.87±1.35	23	8.65±1.36	23	8.52±1.34
	Day 14^a^	20	9.74±1.29	22	9.48±1.18	18	9.71±1.14	18	9.84±1.35
Reticulocyte count (%)	Day 0	23	3.54±2.42	28	3.32±2.15	25	3.09±2.29	26	4.17±3.33
	Day 3	23	2.85±2.25	23	3.81±3.28	21	3.44±2.23	21	2.57±2.94
	Day 7	21	6.72±4.99	24	7.12±4.79	18	6.87±4.90	21	7.55±7.58
	Day 14^a^	20	7.97±9.05	17	4.80±6.30	16	3.93±3.52	14	4.74±4.11
Methemoglobin (%)	Day 0	19	0.39±0.31	25	0.43±0.34	25	0.63±0.58	27	0.30±0.26
	Day 3	21	2.26±3.21	21	2.26±2.16	21	2.49±2.67	21	2.70±3.43
	Day 7	22	0.79±1.46	26	0.40±0.36	20	0.50±0.40	22	0.41±0.27
	Day 14^a^	13	0.38±0.28	16	0.36±0.39	7	0.53±0.46	11	0.39±0.28
Platelets (×10^9^/L)	Day 0	24	140.8±68.4	28	149.3±86.2	25	126.6±64.0	28	134.0±86.9
	Day 3	23	167.3±72.6	26	204.8±107.1	21	177.5±98.7	24	157.2±72.8
	Day 7	22	253.0±133.9	26	289.8±152.9	23	282.0±96.1	23	292.7±109.7
	Day 14^a^	20	280.6±119.9	22	354.0±153.5	17	258.0±119.8	18	256.9±108.9

a Day 14 values were obtained only if Day 7 values were abnormal.

**Table 7:** Number of patients with a decrease in hemoglobin of ≥2 and ≥4 g/dL from baseline for adults and children in the intent to treat (safety) population.

Study day	Number of patients n/N (%) with hemoglobin decrease vs. baseline of:	CPG–DDS	+artesunate 1 mg/kg	+artesunate 2 mg/kg	+artesunate 4 mg/kg
***Adults***		N = 29	N = 30	N = 30	N = 27
Day 3	≥2 g/dL	8/25 (32.0)	8/28 (28.6)	4/25 (16.0)	7/26 (26.9)
	≥4 g/dL	2/25 (8.0)	4/28 (14.3)	0/25	1/26 (3.8)
Day 7	≥2 g/dL	11/22 (50.0)	12/27 (44.4)	8/22 (36.4)	11/22 (50.0)
	≥4 g/dL	4/22 (18.2)	8/27 (29.6)	3/22 (13.6)	1/22 (4.5)
Day 14	≥2 g/dL	4/11 (36.4)	4/13 (30.8)	3/10 (30.0)	5/8 (62.5)
	≥4 g/dL	0/11	2/13 (15.4)	1/10 (10.0)	0/8
***Children***		N = 24	N = 29	N = 26	N = 28
Day 3	≥2 g/dL	5/23 (21.7)	4/27 (14.8)	2/22 (9.1)	3/23 (13.0)
	≥4 g/dL	0/23	0/27	0/22	0/23
Day 7	≥2 g/dL	2/23 (8.7)	2/27 (7.4)	2/22 (9.1)	4/23 (17.4)
	≥4 g/dL	1/23 (4.3)	0/27	0/22	1/23 (4.3)
Day 14	≥2 g/dL	0/20	0/22	1/17 (5.9)	1/18 (5.6)
	≥4 g/dL	0/20	0/22	0/17	0/18

Baseline hemoglobin levels were lower in children than in adults, and decreased further at Day 3 (Table 6). However, in contrast to adults, there was a rebound in hemoglobin levels in children at Day 7 and 14 (Table 6). When combining all child groups, 10.5% (10/95) of children had a decrease in hemoglobin concentration from baseline of ≥2 g/dL and 2.1% (2/95) had a decrease of ≥4 g/dL at Day 7, though by Day 14 only two children had a decrease in hemoglobin concentration from baseline of ≥2 g/dL and none had a decrease ≥4 g/dL (Table 7).

All subjects experienced a rise in methemoglobin levels by Day 3, though in all subjects this had decreased to approximately baseline levels by Day 7 (Table 6). There were no symptoms attributable to methemoglobinemia. The highest methemoglobin level recorded was 15.9% in an adult patient receiving CPG−DSS +artesunate 2 mg/kg and the highest recorded level in a child was 14.2% (received CPG−DDS alone).

## Discussion

### Interpretation

The addition of artesunate to CPG–DDS demonstrated advantages over CPG–DDS alone for the primary efficacy endpoint (mean time to PC90) and for the majority of secondary endpoints in the adult group.

This study achieved very accurate artesunate dosing to the target groups. However, the final fixed dose triple combination tablet needs to allow for a wider range of eventual mg/kg doses based on the weight of the individual patient. For example, for a target dose of 2 mg/kg artesunate, some patients would be expected to actually receive only 1 mg/kg. Thus, the outcome decision matrix was constructed conservatively; i.e. to confidently select the +artesunate 2 mg/kg target dose for the fixed dose combination, the +artesunate 1 mg/kg dose would have needed to be statistically different from CPG–DDS alone for all endpoints.

In children, +artesunate 1 mg/kg was not significantly different from CPG−DDS alone for mean time to PC90 (the primary endpoint) and for mean time to PC50 and PC99. In adults, although +artesunate 1 mg/kg was significantly different from GPG−DDS alone for the mean time to PC90 (the primary endpoint) in the principal (logistic model) analysis, this was not supported by the sensitivity analysis using observed data. In addition, +artesunate 1 mg/kg was not significantly different from CPG−DDS alone for mean time to PC50 in adults. Although patient numbers were small there was no statistical difference in the effect on parasite viability of +artesunate 1 mg/kg versus CPG−DDS alone in adults. There were no apparent differences in the safety profile of CPG−DDS alone or in combination with artesunate in this study and no artesunate dose-related changes in laboratory parameters were observed. Thus, 4 mg/kg artesunate was selected as the dose to be combined with CPG−DDS in the fixed dose combination tablet for further investigation in Phase III studies.

### Generalizability

We examined the differential effect of adding artesunate to CPG−DDS alone. This study was powered to determine the effect of the artesunate doses versus CPG−DDS alone and not to compare the different artesunate doses against each other. In this study a mean time to PC90 of 9 h was assumed as the difference between the artesunate groups and CPG−DDS alone. When comparing between artesunate doses, we would expect a smaller difference in mean time to PC90, and as the artesunate dose increases so the planned difference decreases. As a consequence, the required sample size increases for each comparison to the point where a study of this type would be impractical at Phase II.

The 14 day follow up used in this study was sufficient to demonstrate differences between the artesunate groups and CPG−DDS alone for the parasitological endpoints. The phase III studies with CDA will include a 28-day follow up, based on recent WHO guidelines [18], and will compare clinical endpoints in a conventional comparative trial. These trials will assume equal clinical efficacy (non-inferiority) between CDA and either CPG−DDS or artemether−lumefantrine and include over 2000 patients in total.

The patients in this study were not selected to be necessarily representative of the typical patient presenting with malaria in this setting. The inclusion and exclusion criteria were stringent and selected particularly to identify patients in whom the necessary data for parasitiological endpoints could be collected in order to achieve the study aim, i.e. to identify the effective dose of artesunate. In particular, the cut-offs for parasite density were narrower than commonly used in studies that use ACPRp as a primary outcome. In children, an initial parasite density of at least 25,000 µL^−1^ was used; lower parasite counts may have resulted in such rapid parasite clearance so as to preclude any comparison of artesunate effect. The maximum parasite load of 100,000 µL^−1^ has been used throughout the CPG−DDS and CDA development plans. This is a compromise between having a robust test of the drug while minimizing the number of patients that might have a parasite count exceeding 250,000 µL^−1^ at any point, i.e. the safety cut off whereby a patient must be withdrawn from the study.

The narrow selection criteria in this study and the requirement to stay in hospital contributed to the screening of a very large number of patients. Unfortunately, recording all the patients that were screened and the reasons for not enrolling them in the study was not practical at all the centers. We believe that screening data would have confirmed that the study patient population was not generally representative of patients presenting with malaria.

Patients in this study were required to stay in hospital for four days and three nights. This had a negative impact on recruitment of this study in Malawi, in particular for adults, necessitating the opening of a second center in The Gambia. As a result, no discrete analysis by center was planned, though center was included in the model for analysis of efficacy. Adverse events were reported less frequently for all treatment groups (for adults) at The Gambia study center compared with the Malawi center. These adverse events were recorded based on the discretion of the investigator, and the observed differences could reflect different clinical perspectives or cultural differences between the two sites. However, as there were no differences in the safety outcomes for CPG−DDS versus the artesunate groups for each center, differences between centers did not impact the overall safety outcome of the study.

Patients in this study were excluded if they had overt signs of immunosuppression, but no HIV testing was performed. In 2006, the estimated proportion of the adult population living with HIV was 14.1% in Malawi and 2.4% in The Gambia and in 2003, it was 14.2% and 2.2%, respectively [22]. A study conducted at the Malawi study site found that HIV positive adults with CD4 cell counts <200 cells/mm^3^ had a higher incidence of malaria than those with counts >500 cells/mm^3^ though the prevalence of malaria was not influenced by CD4 count [23]. Thus, it is possible that the proportion of HIV-infected individuals in the study who, by definition, had reported to hospital with malaria, may be higher than in the general population. The effect of the different HIV prevalence for the two centers is unknown. To address this, as well as other possible confounders, ‘center’ was included in the analysis of covariance in the efficacy model. However, it is important to note that, as the efficacy conclusions of the study are based only on data from children, all of whom were recruited from Malawi, any effect on efficacy between the centers would not have changed the outcome of this study.

For children, the median age in the CPG–DDS +artesunate 1 mg/kg group was 46 months, compared with 29−41 months in the other groups. This difference may have affected the results as younger children tend to get more severe malaria. However, the direction of any effect would have been to make 1 mg/kg artesunate appear better than it would have given a more equal balance of ages across the groups, and so this would not have affected the outcome of the decision matrix for dose selection.

When this trial was conceived, it was unknown whether the sparse blood sampling achievable in children would be sufficient to show any treatment differences. An adult group was, therefore, included to provide a more closely sampled data set. However, it seems that despite less frequent sampling in children, the parasitological endpoints were sensitive enough to show differences between the artesunate groups when each was compared to CPG−DDS alone.

It was hoped that parasite viability would provide a robust key secondary endpoint to inform interpretation of the mean time to PC90 data. However, a systematic error in the technique that was later corrected meant that the number of patients included in this analysis was less than we had planned; 40/85 (47.1%) of adults and 13/92 (14.1%) of children were excluded. Thus, the confidence intervals around the mean treatment difference were too large to make relevant comparisons of the differential effect of the artesunate doses versus CPG−DDS alone. However, the data are still interesting. As would be expected, parasite viability did show the potent schizonticidal activity of artesunate, demonstrating the value of adding this drug to the already clinically effective CPG−DDS combination. Unexpectedly, even for the CPG−DDS group, where no effect on parasite viability would be anticipated from this anti-folate inhibitor, parasite viability in adults was only 12.4% versus 71.2% for children. It may be that in adults there is some residual, long-lived immune-related process contributing to the impairment of ex-vivo parasite viability. This observation requires further investigation.

### Overall evidence

It was not practical/ethically justifiable to include an artesunate monotherapy arm in the current study due to the long (7-day) course of treatment required and the risk of developing resistance to artemisinins. The ACPRp at Day 28 for 4 mg/kg artesunate monotherapy has been reported as 72% (36/50) for 3-day dosing [24], and 90% (45/50) for 5-day dosing in Gabonese children (≤15 years) [25]. In the current study, the ACPRp of CPG−DDS plus 4 mg/kg artesunate was 100% at Day 14, indicating that combining artesunate with CPG−DDS allows effective short-course therapy.

A number of clinical studies have evaluated artesunate combination therapy in the treatment of uncomplicated falciparum malaria in African children. However, data comparing different doses of artesunate within combination therapy have been lacking.

A study conducted in Thailand by Angus et al. in patients of at least 15 years of age found that 2 mg/kg artesunate was the lowest dose that, on average, achieved maximum effects on parasite clearance outcomes [7]. However, these authors also pointed out that there was considerable pharmacokinetic and pharmacodynamic variance between individuals and that a dose of 4 mg/kg artesunate would be the minimum required to ensure a maximal effect in all individuals [7]. Similarly, in the current study, mean effects of +artesunate 2 mg/kg and 4 mg/kg versus CPG−DDS were similar for all efficacy outcomes in adults. However, to allow for dosing variations when using a fixed dose combination and the inter-subject pharmacokinetic variability seen with artesunate, the decision matrix selected the dose above that which showed, *on average*, acceptable efficacy. This strategy aims to reduce the potential for resistance emergence and spread by achieving maximal parasite reduction in as many treatments as possible.

There appeared to be a dose−response effect in this study for parasite clearance with artesunate up to 4 mg/kg in children, but not in adults, though this was not tested formally. Children have immature immune systems and more limited exposure to *P. falciparum* and are thus a more sensitive population in which to test the effect of different artesunate doses. Children are also a more vulnerable population clinically and it is important to minimize the possibility of breakthrough parasitemia and recrudescence in this group. Although the children in the study were all over 13 months of age, we believe the differences in outcome observed between adults and children were due primarily to differences in immunological response to *P. falciparum*. This is supported by the observation of far higher levels of parasite viability in children than in adults at 12 h after the first dose of study treatment for CPG−DDS alone and the need for higher doses of artesunate to effectively suppress parasite viability in children versus adults. A subsequent study is planned to determine the safety and efficacy of CDA in children of ≤12 months.

### Safety

Hematological effects are a known adverse event associated with the DDS component [26]. DDS reduces erythrocyte lifespan, causing methemoglobinemia and hemolysis, which may be affected by dose and duration of exposure [26]. These effects are usually mild and self-limiting, as observed for the majority of patients in this study and previously [15]. The frequency of hemolysis requiring blood transfusion in this clinical trial (0.89% [2/223]) is similar to that observed in a previous clinical trial of CPG−DDS alone in five African countries in which 10/1480 patients (0.67%) had a serious adverse event of hemolysis [15].

Erythrocyte survival is further shortened in subjects given DDS who are glucose-6-phosphate (G6PD) deficient [26]. In July 2004, a WHO technical consultation recommended that, as an interim measure, patients should be screened for G6PD deficiency before being given CPG−DDS, though G6PD-deficient patients should not necessarily be excluded from clinical trials [27]. The current study was started before this recommendation, against a background of widespread use of DDS without G6PD screening. It is, therefore, unknown whether the two cases of hemolysis reported here were in G6PD-deficient patients. To determine any significant differences in hematological effects for DDS between G6PD normal and deficient individuals would require a large study to evaluate rare events in this subpopulation; approximately 2500−10,000 patients depending on the prevalence of G6PD deficiency. To further inform the hematological safety profile of CDA, G6PD testing is being conducted in CDA Phase III studies including as comparators CPG−DDS alone and artemether−lumefantrine.

Although this study was not powered to make a formal assessment of safety outcomes between the treatment groups, these did not appear to be influenced by artesunate dose. Safety outcomes will be investigated more fully in a phase III trial of CDA versus CPG−DDS alone.

### Conclusion

Artemisinin-derived compounds, such as artesunate, are included in combination therapy to rapidly reduce parasite load, for their gametocytocidal effect−potentially reducing transmission− and to preserve antimalarial effectiveness by reducing the potential for the development of parasite drug resistance. Clinically, they reduce fever rapidly. However, where the other components of combination therapy are effective, artemisinins have little impact on the WHO definition of adequate clinical and parasitological response at Day 14 or Day 28.

This study demonstrates the use of parasitemia-related outcomes, specifically mean time to PC90, in a clinical trial to make decisions regarding the appropriate artesunate dose to use with an already clinically effective antimalarial combination therapy (CPG−DDS). Based on the findings of this study, Phase III clinical trials with CDA are progressing with a fixed dose combination of 2 mg/kg CPG, 2.5 mg/kg DDS and 4 mg/kg artesunate.

## Supporting Information

Protocol S1 Trial Protocol
(0.74 MB PDF)

Checklist S1 CONSORT Checklist
(0.05 MB DOC)
