# Supplementary material for: Open-Label Comparative Clinical Study of Chlorproguanil−Dapsone Fixed Dose Combination (Lapdap™) Alone or with Three Different Doses of Artesunate for Uncomplicated Plasmodium falciparum Malaria
Source: PLoS One. 2008 Mar 5;3(3):e1779. doi: 10.1371/journal.pone.0001779 (PMC2258152; doi:10.1371/journal.pone.0001779)
Supplement: Protocol S1 — Trial Protocol (0.74 MB PDF) [file pone.0001779.s001.pdf]

Title:

An open, randomised, multi-centre, dose-ranging phase II study to evaluate LAPDAP in combination with three different doses of artesunate.

Document Number: GM2002/00289/04
Study Identifier: SB-714703/003
GSK Compound Number: SB-714703
Issue Date: 09 JUL 2004
Protocol Amendment Number: 04

Author(s): Trudie Lang, Peter Winstanley, Steve Ward, Malcolm Molyneux, William Watkins, Nick White, Karen Ooms, Dawn Webber, Paula Kirby, Stephan Duparc, Colin Neate, Sharon Barton, Ann K Miller

Revision Chronology:

GM2002/00289/00	2002-DEC-16	Original
GM2002/00289/01	2003-APR-16	Amendment No.: 01 To incorporate safety changes to protocol
GM2002/00289/02	2003-DEC-11	Amendment No.: 02 To modify the analysis populations, add an additional endpoint.
GM2002/00289/03	2004-MAY-05	Amendment No.: 03 To amend inclusion criteria relating to parasitaemia entry level
GM2002/00289/04	2004-JUL-09	Amendment No.: 04 To add details of a second site in The Gambia

Copyright 2004 the GlaxoSmithKline group of companies. All rights reserved. Unauthorised copying or use of this information is prohibited.

CONFIDENTIAL
The GlaxoSmithKline group of companies
GM2002/00289/04
SB-714703/003

**Sponsor Signatory:**	**Signature:**	**Date:**
Dr Clarence Young<br>VP, N. America, Infectious Diseases MDC	[signature]	[illegible]

SPONSOR INFORMATION PAGE

Title:	An open, randomised, multi-centre, dose-ranging phase II study to evaluate **LAPDAP*** in combination with three different doses of Artesunate
Study Identifier:	SB-714703/003

GlaxoSmithKline
Greenford Road
Greenford, Middlesex, UB6 0HE, UK
Telephone: +44 (0)20 8 422 3434

Sponsor Contact Information: Paula Kirby

Medical Monitors: William Mwatu, Yves Aphing-Kouassi

GSK study Clinician: Stephane Duparc

INVESTIGATOR AGREEMENT PAGE

I agree:

- To assume responsibility for the proper conduct of the study at this site.
- To conduct the study in compliance with this protocol amendment and with any other study conduct procedures provided by GlaxoSmithKline (GSK).
- Not to implement this protocol amendment without agreement from the sponsor and prior submission to and written approval from (where required) the Institutional Review Board (IRB) or Independent Ethics Committee (IEC), except when necessary to eliminate an immediate hazard to the subjects, or for administrative aspects of the study (where permitted by all applicable regulatory requirements).
- Note to implement any other changes to the protocol without agreement from the sponsor and prior review and written approval from the IRB or IEC, except where necessary to eliminate an immediate hazard to the subjects, or for administrative aspects of the study (where permitted by all applicable regulatory requirements).
- That I am thoroughly familiar with the appropriate use of the investigational product(s), as described herein, and any other information provided by the sponsor including, but not limited to, the following: the current Investigator's Brochure (IB) or equivalent document, IB supplement (if applicable), and approved product label (if the product is marketed in this country and the label is not already provided as an equivalent to an IB).
- That I am aware of, and will comply with, "good clinical practices" (GCP) and all applicable regulatory requirements.
- To ensure that all persons assisting me with the study are adequately informed about the GSK investigational product(s) and of their study-related duties and functions as described herein.

Investigator Name: ______________________________

______________________________ ______________________

Investigator Signature Date

TABLE OF CONTENTS

	Page
ABBREVIATIONS	9
PROTOCOL SUMMARY	10
1. INTRODUCTION	13
1.1. Background	13
1.2. Rationale	15
2. OBJECTIVE(S)	15
2.1. Primary	15
2.2. Secondary	16
3. ENDPOINT(S)	16
3.1. Primary Endpoint	16
3.2. Secondary Endpoints	16
3.2.1. Efficacy	16
3.2.2. Safety	17
3.2.3. Pharmacokinetics	17
4. STUDY DESIGN	17
5. STUDY POPULATION	18
5.1. Number of Subjects	18
5.2. Eligibility Criteria	19
5.2.1. Inclusion Criteria	19
5.2.2. Exclusion Criteria	19
5.2.3. Other Eligibility Criteria Considerations	20
6. STUDY ASSESSMENTS AND PROCEDURES	20
6.1. Demographic and Baseline Assessments	20
6.2. Study Visits	20
6.3. Safety	22
6.3.1. Pregnancy	22
6.4. Efficacy	23
6.4.1. Parasite Count	23
6.4.2. Parasite Viability	24
6.4.3. Treatment failure	24
6.4.4. PCR analysis	25
6.4.5. Temperature	26
6.4.6. Gametocyte analysis	26

6.5. Pharmacokinetics 26

7. INVESTIGATIONAL PRODUCT(S) 27
7.1. Description of Investigational Product 27
7.2. Dosage and Administration 27
7.3. Dose Rationale 27
7.4. Blinding 28
7.5. Treatment Assignment 28
7.6. Packaging and Labelling 28
7.7. Preparation 29
7.8. Handling and Storage 29
7.9. Product Accountability 29
7.10. Assessment of Compliance 29
7.11. Treatment of Investigational Product Overdose 29
7.12. Occupational Safety 30

8. CONCOMITANT MEDICATIONS AND NON-DRUG THERAPIES 30
8.1. Permitted Medications 30
8.2. Prohibited Medications 30

9. SUBJECT COMPLETION AND WITHDRAWAL 30
9.1. Subject Completion 30
9.2. Subject Withdrawal 31
9.2.1. Subject Withdrawal from Study 31
9.2.2. Subject Withdrawal from Investigational Product 31

10. ADVERSE EVENTS (AE) AND SERIOUS ADVERSE EVENTS (SAE) . . 31
10.1. Definition of an AE 32
10.2. Definition of a SAE 33
10.2.1. Disease-Related Events or Outcomes Not Qualifying as SAEs 33
10.3. Lack of Efficacy 34
10.4. Clinical Laboratory Abnormalities and Other Abnormal Assessments as AEs and SAEs 34
10.5. Time Period, Frequency, and Method of Detecting AEs and SAEs . 34
10.6. Recording of AEs and SAEs 34
10.7. Evaluating AEs and SAEs 35
10.7.1. Assessment of Intensity 35
10.7.2. Assessment of Causality 35
10.8. Follow-Up of AEs and SAEs 35
10.9. Prompt Reporting of SAEs to GSK 36

10.9.1. Timeframes for Submitting SAE Reports to GSK 36
10.9.2. Completion and Transmission of the SAE Reports 36
10.10. Regulatory Reporting Requirements For SAEs 37
10.11. Post-study AEs and SAEs 37
10.12. SAEs Related to Study Participation 38

11. DATA ANALYSIS AND STATISTICAL CONSIDERATIONS 38
11.1. Hypotheses 38
11.2. Treatment Comparisons of Interest 38
11.2.1. Primary Comparisons of Interest 38
11.2.2. Other comparisons of interest 39
11.3. Interim Analysis 39
11.4. Sample Size Considerations 39
11.4.1. Sample Size Assumptions 39
11.4.2. Sample Size Sensitivity 39
11.4.3. Sample Size Re-estimation 40
11.5. Analysis Populations 40
11.5.1. Data Sets 40
11.6. General Considerations for Data Analysis 40
11.6.1. Withdrawal 41
11.6.2. Missing Data 41
11.6.3. Derived and Transformed Data 41
11.6.4. Assessment Windows 41
11.7. Efficacy Analyses 41
11.7.1. Primary Analysis 42
11.7.2. Secondary Analysis 42
11.8. Safety Analyses 44
11.8.1. Extent of Exposure 44
11.8.2. Adverse Events 44
11.8.3. Clinical Laboratory Evaluations 44
11.9. Clinical Pharmacology Data Analyses 44
11.9.1. Pharmacokinetic Analyses 44

12. STUDY ADMINISTRATION 45
12.1. Regulatory and Ethical Considerations 45
12.1.1. Regulatory Authority Approval 45
12.1.2. Ethical Conduct of the Study and Ethics Approval 45
12.1.3. Informed Consent 45
12.1.4. Investigator Reporting Requirements 46

12.2. Study Monitoring 46
12.3. Quality Assurance 46
12.4. Study and Site Closure 47
12.5. Records Retention 47
12.6. Provision of Study Results and Information to Investigators 48
12.7. Information Disclosure and Inventions 48
12.8. Data Management 49

13. REFERENCES 50

14. APPENDICES 52
14.1. Appendix 1: Adult Time and Events Table 52
14.2. Appendix 2: Children Time and Events Table 53
14.3. Appendix 3: Informed Consent Form; Malawi 54
14.4. Appendix 4: Informed Consent Form; The Gambia 57
14.5. Appendix 5: Dosing charts 60
14.6. Appendix 6: Protocol Amendment 1 68
14.7. Appendix 7: Protocol amendment 2. 71
14.8. Appendix 8: Protocol amendment 3. 77
14.9. Appendix 9: Protocol amendment 4 79

ABBREVIATIONS

AE	Adverse Event
ART	Artesunate
AQ	Amodiaquine
CCG	Chlorcycloguanil
CDA	Chlorproguanil-dapsone-artesunate
CIB	Clinical Investigator Brochure
CPG	Chlorproguanil
CQ	Chloroquine
CRF	Case Record Form
CT	Combination Therapy
DDS	Dapsone
DHA	Dihydroartemisinin
DHFR	Dihydrofolate reductase
GCP	Good Clinical Practice
GSK	GlaxoSmithKline
IB	Investigator Brochure
IEC	Independent Ethics Committee
IRB	Institutional Review Board
IV	Intravenous
MADDS	Mono acetyl dapsone
PC50, 90,99	Parasite count, of 50, 90 or 99% of baseline value
PCR	Polymerase Chain Reaction
PCT	Parasite Clearance Time
PDT	Product Development Team
PK	Pharmacokinetic(s)
PRBC	Parasitised Red Blood Cell
PV	Parasite Viability
QC	Quality Control
SAE	Serious Adverse Event
SP	Sulphadoxine Pyrimethamine
WHO	World Health Organisation
WHO-TDR	WHO-Tropical Disease Research

Trademark Information

Trademarks of the GlaxoSmithKline group of companies
LAPDAP

Trademarks not owned by the GlaxoSmithKline group of companies

PROTOCOL SUMMARY

Rationale

The rate at which resistance to antimalarial drugs is developing exceeds the rate of presentation of new drugs. If a disastrous rise in malaria-associated mortality and morbidity is to be avoided in Africa, rational use of the remaining drugs is essential.

Combining drugs with different modes of action has a strong theoretical basis, and is supported by limited trials in both Southeast Asia, and East Africa. GSK and its external partners are developing chlorproguanil-dapsone-artesunate (CDA) as a fixed ratio 'combination therapy'. It is anticipated that CDA will have important advantages over **LAPDAP**[*] (chlorproguanil and dapsone). However, the dose of the artesunate component requires careful study, both for empirical reasons and with an eye on minimising cost.

This study will be conducted in both adults (men and women) and children:

- CDA will probably have more public health impact in young children. This group's protective immunity to malaria is less complete than in adults. Hence the importance to verify the artesunate dose in children.
- The present study requires parasitological data of two kinds: (1) data that can be obtained easily from both children and adults, and (2) data that requires multiple blood venous sampling, which is difficult in children with malaria.
- Therefore this study will obtain 'rich' data in adults with which the dose can be selected, and ' sparse' data in children that can confirm this selection of dose.

Objective(s)

Primary:

- To determine the optimum dose of artesunate for the CDA project by assessing the reduction in parasite density, when administered with an established dose of **LAPDAP** to adults and children with uncomplicated *P. falciparum* malaria.

Secondary:

- To determine the safety and tolerability of artesunate when administered in combination with **LAPDAP** to adults and children with uncomplicated *P. falciparum* malaria.
- To determine the pharmacokinetic profiles of the component drugs when artesunate is administered with an established dose of **LAPDAP** to adults with uncomplicated *P. falciparum* malaria.

---

[*] LAPDAP is a Trade Mark of the GlaxoSmithKline group of companies.

Endpoint(s)

Primary Efficacy Endpoint:

- PC90 determined from blood slide data (PC90 is reduction of parasite count by 90% of that recorded at enrolment), determined from 'rich' adult data and confirmed with more sparse child data.

Secondary Endpoints:

- Parasite viability, determined from 'rich' adult data and confirmed with more sparse child data.
- PC50 and PC99 determined from blood slide data, determined from 'rich' adult data and confirmed with more sparse child data.
- Proportion gametocytaemic and gametocyte density, all subjects
- Early or late treatment failures (WHO Definitions), all subjects
- Chlorproguanil (CPG), chlorcycloguanil (CCG), dapsone (DDS), mono acetyl dapsone (MADDS), artesunate (ART) and dihydroartemisinin (DHA) plasma levels, in adults only.
- Reduction in Temperature, all subjects
- Adverse event details, all subject
- Haematology and biochemistry results, determined from 'rich' adult data and confirmed with more sparse child data

Study Design

This is an open-label, dose-ranging study of a fixed dose of **LAPDAP** plus 0, 1, 2 or 4mg/kg artesunate. Subjects will be randomised on entry into the trial into one of the four treatment groups, stratified by gender in the adult population, and by age in the children.

All subjects will receive study drug for 3 days (days 0, 1, 2), during which time they will be hospitalised for study-related procedures. Subjects will be discharged after completing study assessments on day 3, and will be asked to return to clinic for outpatient follow-up visits on days 7 and 14.

Study Population

120 Adults (men and women) and 120 children (between 12-120 months) with uncomplicated *P. falciparum* malaria will be recruited from two sites; one in Malawi, and a second in The Gambia, recruiting adults only. These numbers are based on having 22 evaluable adult subjects per treatment group, based on the PC90 sample size calculation. Allowing for a 25% drop-out rate, 30 subjects per group are required. An equal number of children will recruited.

Study Assessments and Procedures

The following assessments will be conducted as part of the study: thick or thin films for parasite count and calculation of PC90, PC50 and PC99; parasite viability analysis; temperature measurement; haematology and biochemistry analysis; collection of adverse event data and pharmacokinetic sampling in adults only.

Investigational Product(s)

The fixed ratio combination of chlorproguanil hydrochloride (CPG) with dapsone (DDS) is being developed by WHO-TDR in collaboration with GlaxoSmithKline as '**LAPDAP**, (SB433372) presented as combination tablets containing either 15/18.75mg CPG/DDS or 80/100mg CPG/DDS.

All subjects will receive the respective tablet(s) to achieve nominal target doses of 2.0mg/kg and 2.5 mg/kg chlorproguanil/dapsone respectively (See Appendix 5: Dosing charts).

For those subjects randomised to receive artesunate, tablets containing either 1mg, 10mg, 25mg or 50 mg artesunate will be provided. Subjects will receive respective number of tablets for their weight to achieve nominal target doses of either 1mg, 2mg or 4mg /kg (See Appendix 5: Dosing charts).

Subjects will be randomised to one of four treatment groups as indicated below. Each subject will receive **LAPDAP**, daily for 3 days orally, plus either 0, 1, 2 or 4mg/kg artesunate.

a **LAPDAP** alone (daily for 3 days)

b **LAPDAP** plus 1 mg/kg artesunate (daily for 3 days)

c **LAPDAP** plus 2 mg/kg artesunate (daily for 3 days)

d **LAPDAP** plus 4 mg/kg artesunate (daily for 3 days)

1. INTRODUCTION

1.1. Background

The main scientific objective of the **LAPDAP** development process is a safe, effective and affordable treatment, especially in Africa, for uncomplicated falciparum malaria.

African countries, especially in east and central Africa, are in the process of changing first line treatment for uncomplicated falciparum malaria from chloroquine (CQ) to either sulfadoxine-pyrimethamine (SP), or to a combination of SP with CQ or amodiaquine (AQ). Resistance to both classes of drugs is a major problem: the speed with which drug resistance is spreading in Africa threatens a public health disaster unless effective counter-measures are employed quickly [White, 1999a; White, 1999b].

Resistance of *Plasmodium falciparum* to almost all antimalarial drugs has become widespread in Southeast Asia, Africa and South America [Wernsdorfer, 1999]. The only exception to this situation is the artemisinin derivatives that have recently been introduced for widespread use in Southeast Asia [Anonymous, 1994]. The fixed combination SP is no longer effective in many areas of Southeast Asia and its efficacy is fading rapidly in East Africa [Nzila, 2000a; Nzila 2000b].

Although the precise antifolate resistance mechanism remains unclear, there is evidence that mutations in the parasite gene encoding dihydrofolate reductase (DHFR) are central to the mechanism [Nzila, 2000a; Nzila 2000b]. Parasites with mutations at codons 108, 51 and 59 in *dhfr* are now common in East Africa: this genotype accounts for the large majority of parasitaemias that remain patent at day 7 after SP treatment. One further mutation, at codon 164, will provide complete clinical resistance to SP, and borderline resistance to **LAPDAP** in a 3-day dosage regimen [Watkins, 1997; Watkins, 1999]. Since the leu-164 mutation arose soon after SP was brought into use in SE Asia, it is likely that it will soon appear in Africa, unless leu-164 carries a biological deficit more severe in African than in Asian *P. falciparum*.

Leu-164, and other mutations of equivalent antifolate resistance, may well be selected by widespread use of SP. Thus, it is hypothesised by some public health scientists that **LAPDAP** monotherapy may enjoy only a short period of utility. Where **LAPDAP** replaces CQ without an interim use of SP, this factor is less serious, because of the short elimination half-life of **LAPDAP**, and consequently lower selective pressure [Nzila , 2000b].

To ensure the optimum useful therapeutic life, many public health scientists would argue that **LAPDAP**, like most other 'traditional' antimalarial drugs, requires combination with a ‘more active’ antimalarial drug, which will reduce parasite biomass quickly [White, 1999a]: the artemisin groups of compounds are first choice.

However, the dose of artesunate that should be used is unclear. Several studies are in progress in Africa, using a combination of artesunate with either a 4-aminoquinoline or antifolate, to assess ‘proof of concept’ of combination therapy (CT). In these trials,

artesunate is being used, uniformly, at a dose of 4 mg/kg body weight. No data are yet available from these double-blind trials.

Three factors of particular importance in considering the artesunate dose in CT formulation are: 1) the contribution of artesunate to the final cost of the medicine, 2) toxicity and 3) efficacy. Despite the evidence, from treatments in thousands of patients [White, 1999b], of the general safety of the water-soluble artemisinin derivatives, including artesunate, concerns are still expressed over possible chronic toxicity following multiple treatments. In addition, the observation of embryotoxicity and morphological abnormalities in the fetuses of rats and rabbits raises concerns about the reproductive safety of this drug class. Well-conducted studies of pregnant women exposed to the artemisinins are reassuring, and the WHO guidelines strongly favour continued deployment of this drug group [WHO, 2002]. These concerns may be impossible to address by standard toxicity testing, but they suggest that the unnecessary exposure of patients to excess of artesunate should be avoided. Cost is also an important consideration for a drug that may be prescribed to huge numbers of people. On the other hand, the dose of artesunate needs to be adequate to achieve desired efficacy.

Professor SA Ward has analysed 20 malaria treatment trials, which used artesunate: 16 from SE Asia, one from S America and three from Africa. [Paper tabled by SAW at the Product Development Team (PDT) meeting, 20th June, 2000]. The analysis demonstrated that parasite clearance times (PCT) are always shorter with artesunate-containing regimens, and that there is no clear relationship between PCT and total dose of artesunate, duration of treatment or second drug.

There is, therefore, a need to determine the appropriate artesunate dose for treatment in combination with **LAPDAP**. In the present study we are interested to define the optimum dose for artesunate in combination with **LAPDAP**. Based on previous published data, we think that an artesunate dose of 1 mg/kg daily for three days will probably be effective, but it is possible that 2 mg/kg/day will prove to have an advantage; we doubt any additional advantage for 4 mg/kg over 2 mg/kg. Knowledge gained from this study will allow the PDT to select an artesunate dose that is the most appropriate: in doing this the PDT will exercise due caution in interpreting the results of the present trial. In a future phase-III trial, chlorproguanil-dapsone-artesunate will be compared with **LAPDAP** monotherapy using clinical endpoints.

It is important to decide the dose from data collected across the potential target population - men, women and children. Children are particularly important, firstly because they are the most at risk group, and secondly they are at an earlier stage in the acquisition of immunity than adults. Therefore their response to the dose is important and data from children are crucial to contributing any decision on what is the most appropriate dose to use. Women are included in this study as they are also within the target treatment population. The ratio of men:women to be recruited is 3:2, and all women will be asked to have a pregnancy test at the beginning and end of the study.

Studying men, women and children in the same protocol is considered the best approach as it limits any potential variation between the malaria itself and any differences between laboratory techniques, both of which could cause differing results. It is important to

appreciate that the use of an in-dwelling IV cannula for multiple venous sampling is considered unethical in this group of children. Hence the importance of data from adults, where multiple venous samples may be drawn.

1.2. Rationale

The rate at which resistance to antimalarial drugs is developing exceeds the rate of presentation of new drugs. If a disastrous rise in malaria-associated mortality and morbidity is to be avoided in Africa, rational use of the remaining drugs is essential.

During treatment with two (or more) drugs, the chance of a mutant resistant to both drugs emerging can be calculated from the product of the individual per-parasite mutation rates (assuming that the resistance mutations are not 'linked'). The artemisinin-derivatives reduce the parasite biomass by around 4-logs for each asexual cycle: this makes them the most rapidly-efficacious antimalarial drugs in use. This rapid reduction of the parasite biomass has a major theoretical role when artemisinin-derivatives are combined with another antimalarial drug: the parasite population available to develop mutations to the second drug is reduced by several log-orders. Thus, when mefloquine was used in combination with ARTs in Thailand, that rate of development of mefloquine resistance was reduced. It now seems likely that WHO will recommend artemisinin CT as part of the 'ideal' strategy for malaria control in Africa. Artemisinin drugs are best used in combination, rather than on their own: (a) they are so rapidly eliminated that monotherapy with artemisinins must continue for a minimum of 7 days, and (b) they are relatively expensive.

It is anticipated that CDA will have important advantages over **LAPDAP**. However, the dose of the artesunate component requires careful study, both for empirical reasons and with an eye on minimising cost.

This study will be conducted in both adults and children as it is important to verify the dose in children. With adults, in whom it is possible to obtain firm consent, multiple blood sampling presents fewer ethical issues than in children, and it is not possible to obtain the same frequency of samples in children. Therefore this protocol will obtain 'rich' data in adults with which the dose can be selected, and ' sparse' data in children that can confirm this selection of dose.

2. OBJECTIVE(S)

2.1. Primary

- To determine the optimum dose of artesunate for the CDA project by assessing the reduction in parasite density, when administered with an established dose of **LAPDAP** to adults and children with uncomplicated *P. falciparum* malaria

2.2. Secondary

- To determine the safety and tolerability of artesunate when administered in combination with **LAPDAP** to adults and children with uncomplicated *P. falciparum* malaria.
- To determine the pharmacokinetic profiles of the component drugs when artesunate is administered with an established dose of **LAPDAP** to adults with uncomplicated *P. falciparum* malaria.

3. ENDPOINT(S)

3.1. Primary Endpoint

The primary endpoint is the determination of PC90 from blood slide data. PC90 is the time to achieve a reduction of the parasitaemia to 90% of baseline. As efficacy with **LAPDAP** alone is 96%, we would not be able to detect a difference in clinical efficacy between the control (**LAPDAP** alone), and the addition of artesunate. Parasite clearance is a good indicator of the activity of artesunate, as artesunate is known to rapidly reduce the parasite load [Angus, 2002]. Differences between the addition of 1, 2, or 4 mg/kg of artesunate to the fixed dose of **LAPDAP** will be investigated.

To detect the PC90 data (and secondary variables PC50, PC99), frequent blood sampling is required over the first 48 hours post-dose (details given in section 6.4.1). As it is not ethical to take such frequent blood samples from children, the procedures will be conducted fully in adults and more sparsely in children. Therefore it will be a matter of confirming the similarity (or otherwise) of the results seen with the adults with the information we have from children.

3.2. Secondary Endpoints

3.2.1. Efficacy

Differences between the addition of 1, 2, or 4 mg/kg of artesunate to the fixed dose of **LAPDAP** will be investigated for the following:

- Parasite viability, determined from ‘rich’ adult data and confirmed with more sparse child data.
- PC50 and PC99 determined from blood slide data, determined from ‘rich’ adult data and confirmed with more sparse child data.
- Proportion gametocytaemic and gametocyte density, all subjects
- Early or late treatment failures (WHO Definitions), all subjects.
- Reduction in temperature, all subjects

Parasite viability will be considered the key secondary variable.

3.2.2. Safety

- Adverse Event details, all subjects. Frequency of adverse events, thought to be drug-related, and severe enough to warrant no further drug exposure.
- Biochemistry and Haematology results, including reticulocyte analysis. Mean haemoglobin concentration will be reported on each follow-up day.
- Development of clinically severe malaria, including a parasite count of >250,000ul^{-1}.

3.2.3. Pharmacokinetics

- Chlorproguanil (CPG), chlorcycloguanil (CCG), dapsone (DDS), mono acetyl dapsone (MADDS), artesunate (ART) and dihydroartemisinin (DHA) plasma levels, in adults only. These data will be used to determine T_{max}, C_{max}, $t_{1/2}$, and AUC for each compound, and to assess the effect of artesunate on the pharmacokinetics of the components of Lapdap, if data permit.

4. STUDY DESIGN

This is an open-label, dose-ranging study of a fixed dose of **LAPDAP** plus 0, 1, 2 or 4mg/kg artesunate. Subjects will be randomised on entry into the trial into one of the four treatment groups, stratified by gender in the adult population, and by age in the children. The stratification is to prevent bias towards men in the adults, as was previously experienced in an earlier **LAPDAP** study and to ensure that the younger children are represented to provide data in this group.

All subjects will receive study drug for 3 days (days 0, 1, 2), during which time they will be hospitalised for study-related procedures. Subjects will be discharged from hospital once they have completed all study-related procedures on day 3. They will be asked to return to clinic for outpatient follow-up visits on days 7 and 14.

Table 1 Time and Events.

	Day 0	Day 1	Day 2	Day 3	Day 7	Day 14
**Diagnosis**	√					
**Pregnancy test, adult women only**	√					√
**Informed consent**	√					
**Medical History**	√					
**Prior medication**	√					
**Concomitant medication**	√	√	√	√	√	√
**Clinical Examination**	√			√	√	√
**Hospitalised**	√	√	√	√		
**Symptom enquiry**	√	√	√	√	√	√
**Temperature***	√	√	√	√	√	√
**Pharmacokinetic sampling, adults only**	√	√	√	√		
**Parasite viability sampling [1]**	√	√				
**Parasitology[1]**	√	√	√	√	√	√
**PCR blood sample collection**	√				√ [3]	√ [3]
**Haematology and Biochemistry**	√			√	√	√[2]
**Supervised Dosing**	√	√	√			
**Adverse events**	√	√	√	√	√	√

1. See section 6.4.1for timings of bloodslides, 6.4.2. for parasite viability, 6.4.4 for temperature.
2. Further testing only required if samples were abnormal at Day 7.
3. Further PCR samples only required on day 7, day 14 if parasites are detected

5. STUDY POPULATION

5.1. Number of Subjects

This trial will be conducted in the Queen Elizabeth Central Hospital, Blantyre, Malawi and at the Farafenni Field Station, MRC laboratories, The Gambia. The trial will be conducted in 120 African adults (men and women) aged 18 – 45, and 120 African children aged 12 to 120 months with uncomplicated falciparum malaria. The majority of malarial disease occurs in the under fives, however it is not ethical to take multiple blood samples from young children, we therefore propose to study both adults and children concurrently. We will obtain rich data from adults and compare sparse data obtained from children. Older children may bias the data as they will begin to acquire partial immunity. Therefore the upper age limit for children has been set at 10 years.

5.2. Eligibility Criteria

5.2.1. Inclusion Criteria

A subject will be eligible for inclusion in this study only if all of the following criteria apply:

1. Presentation to a healthcare facility with probable uncomplicated clinical malaria.
2. Adults aged between 18 and 60 years , or children aged between 12 and 120 months
3. Weigh between 5 and 85kg.
4. Pure [on microscopic grounds] screening *P. falciparum* parasitaemia in children from 25,000 to 100,000 ul^{-1}, or in adults from 10,000 to 100,000 ul^{-1}.
5. Written or oral witnessed consent has been obtained from subject, parent or guardian.
6. Is willing to comply with the requirements of the protocol and particularly to remain in hospital for four days and 3 nights, and to have blood taken at intervals, by finger-prick (children) or via a cannula (adults).
7. For women of child-bearing age, have had a negative pregnancy test on enrolment.

5.2.2. Exclusion Criteria

A subject will not be eligible for inclusion in this study if any of the following criteria apply:

1. Features of severe/complicated falciparum malaria.
2. Known allergy to sulphonamides.
3. Evidence of any concomitant infection at the time of presentation (including P. ovale and P. malariae).
4. Any other underlying disease that may compromise the diagnosis and the evaluation of the response to the study medication (including clinical symptoms of immunosuppression, tuberculosis and bacterial infection).
5. Treatment within the past twenty-eight days with sulfadoxine/pyrimethamine (Fansidar, Celoxine), sulfalene/pyrimethamine (Metakelfin), mefloquine-sulfadoxine-pyrimethamine (Fansimef), chloroquine* (Nirupquine); 21-days with mefloquine, or 7-days with amodiaquine, halofantrine, quinine (full course), atovaquone - proguanil, artemisinins, co-artemether, tetracycline or clindamycin, or 5 half-lives for any drugs with a potential anti-malarial activity (e.g. co-trimoxazole in the previous 60 hours).
6. Use of an investigational drug within 30 days or 5 half-lives whichever is the longer.
7. Previous participation in this study.
8. For women of child-bearing age, subjects who have had a positive pregnancy test at enrolment, or do not give their consent to take a pregnancy test.
9. Female subjects who will be breast-feeding an infant for the duration of the study.

* Prior chloroquine use will be determined by questioning, with supporting evidence of CQ administration. If there is uncertainty about the identity of an anti-malarial taken in the previous 28-days, the subject will be asked to take a qualitative urinary test to determine if they have received chloroquine or not.

5.2.3. Other Eligibility Criteria Considerations

To assess any potential impact on subject eligibility with regard to safety, the investigator must refer to the following document(s) for detailed information regarding warnings, precautions, contraindications, adverse events, and other significant data pertaining to the investigational product(s) being used in this study: Investigator's Brochure.

6. STUDY ASSESSMENTS AND PROCEDURES

Subjects will be screened on presentation at an outpatient facility. If suitable they will be transported to the hospital for enrolment into the study. If assessment is permitted, and the subject fulfils the entry criteria, including a negative pregnancy test for adult women, then the study will be explained in the subject's preferred language to them or, for children, explained to their parent or guardian (hereafter referred to as 'guardian'). Written informed consent of the subject or their guardian, or oral witnessed consent (if the subject/guardian is illiterate) shall be obtained on approved forms (see Appendix 3 and section 12.1.3) prior to any study specific tests being performed. A screening record will be maintained to log all subjects that were considered as potential subjects.

6.1. Demographic and Baseline Assessments

The subject's date of birth will be noted in the CRF. In addition, a full medical history will be recorded to include details of significant past medical/surgical history, demography and concurrent illness. Any prior (previous seven days) and concomitant medication will be recorded. The subjects will undergo a physical examination, evaluation of vital signs and baseline adverse experiences will be recorded.

6.2. Study Visits

Day 0:
After enrolment the subject will be admitted to the ward designated for the study. An intravenous cannula will be inserted (adults only), which will be kept patent with heparinised saline for the inpatient stay. The study procedures and timings are summarised in Appendix 1 and Appendix 2, for adults and children respectively.

Prior to dosing a sample of <4.5ml will be taken from adults, and <2.5 ml from children, for the following time 0 samples:

- 2ml PK sample from adults only
- 2ml biochemistry and haematology, all subjects (see Table 2, section 6.3)
- 10ul for parasitaemia blood slide, all subjects

- 50ul for PCR filter paper preparation (see section 6.4.4)
- 100ul for parasite viability, all subjects

The subject will then be dosed (and the time noted). After dosing the PK sampling and parasite viability will commence as shown in Table 6, section 6.5, and Table 4 section 6.4.2 respectively. A bloodslide will be prepared at the times shown in Table 3, section 6.4.1. Any changes in symptoms will be recorded if they meet the criteria for an adverse event.

Day 1.
Prior to receiving the second dose, the subject (or their guardian) will be questioned about symptoms and the subject will be examined (see CRF). Any change in concomitant medication will be recorded, and the 24 hour samples will be taken. The subject can then be given the second dose of treatment medication. Sampling will continue for PK, parasitology and parasite viability according to the schedule.

Day 2.
Prior to receiving the final dose the subject (or their guardian) will be questioned about symptoms and the subject examined (see CRF). Any change in concomitant medication will be recorded, and the 48 hour samples will be taken. The subject can then be given the third dose of treatment medication. Sampling will continue for PK, parasitology and parasite viability according to the schedule.

Day 3.
The subject (or their guardian) will be questioned about symptoms and the subject examined (see CRF). Any change in concomitant medication will be recorded. Sampling will continue for PK and parasitology, and a sample will be taken for biochemistry and haematology. After the PK sampling has been completed the subject will receive a clinical examination and, if judged to be well enough, will then be discharged from the ward and asked to return in four days' time.

Day 7.
The subject should return to the hospital. If they do not attend, they should be followed up by a visit to their home to ask them to attend the clinic.

The subject (or their guardian) should be asked about their malaria symptoms, and if they have felt different in any way (any adverse events). Any change in concomitant medication should be recorded. A 2ml venous sample will be taken for parasitology, biochemistry and haematology. If parasites are detected, a filter paper should be prepared for analysis. The subject should have a clinical examination and their temperature will also be recorded. The subject will then be asked to return in one week.

Day 14.
The subject should return to the hospital. If they do not attend, they should be followed up by a visit to their home to ask them to attend the clinic.

The subject (or their guardian) should be asked about their malaria symptoms, and if they have felt different in any way (any adverse events). Any change in concomitant medication should be recorded. A thumbprick blood sample will be taken for parasitology. If parasites

are detected, a filter paper should be prepared for PCR analysis. The subject should have a clinical examination and their temperature will also be recorded. Adult women will be asked to have a pregnancy test. If the biochemistry or haematology results were abnormal at day 7 then these need to be repeated by venous sample (2ml).

6.3. Safety

Safety will be evaluated by collecting Adverse Event information from the time of first dose to end of follow-up (day 14, unless subject has been withdrawn).

Two millilitre blood samples will be taken for haematology and biochemistry analysis at the times indicated in Table 2 below.

Table 2 Laboratory Analysis time and events

	Day 0	Day 3	Day 7	Day 14
Haemoglobin	√	√	√	Only if abnormal at Day 7
Reticulocyte count	√	√	√	Only if abnormal at Day 7
Red blood cell count	√	√	√	Only if abnormal at Day 7
White cell count	√	√	√	Only if abnormal at Day 7
Platelets	√	√	√	Only if abnormal at Day 7
Methaemoglobin	√	√	√	Only if abnormal at Day 7
Serum creatinine	√	√	√	Only if abnormal at Day 7
Plasma Bilirubin	√	√	√	Only if abnormal at Day 7
AST	√	√	√	Only if abnormal at Day 7
ALT	√	√	√	Only if abnormal at Day 7

6.3.1. Pregnancy

6.3.1.1. Pregnancy testing

All women of child-bearing potential will be asked to take a serum pregnancy test at screening for the study. If consent is not given to take a pregnancy test, they will not be eligible to participate in the study. A second urinary pregnancy test will be performed on day 14.

6.3.1.2. Time period for collecting pregnancy information

Pregnancies will be reported from the time of first dose to end of follow-up (day 14, unless the subject is withdrawn earlier).

6.3.1.3. Action to be taken if pregnancy occurs

The investigator, or his/her designee, will collect pregnancy information on any female subject who becomes pregnant while participating in this study. The investigator, or his/her designee, will record pregnancy information on the appropriate form and submit it

to GSK within 2 weeks of learning of a subject's pregnancy. The subject will also be followed to determine the outcome of the pregnancy. Information on the status of the mother and child will be forwarded to GSK. Generally, follow-up will be no longer than 6 to 8 weeks following the estimated delivery date. Any premature termination of the pregnancy will be reported.

While pregnancy itself is not considered to be an AE or SAE, any pregnancy complication or elective termination of a pregnancy for medical reasons will be recorded as an AE or a SAE, as described in Section 10.6., "Recording of AEs and SAEs" and will be followed as described in Section 10.8., "Follow-up of AEs and SAEs."

A spontaneous abortion is always considered to be a SAE and will be reported as described in Section 10, "Adverse Events (AE) and Serious Adverse Events (SAE)." Furthermore, any SAE occurring as a result of a post-study pregnancy **and** is considered reasonably related to the investigational product by the investigator, will be reported to GSK as described in Section 10.11., "Post-study AEs and SAEs." While the investigator is not obligated to actively seek this information in former study participants, he/she may learn of an SAE through spontaneous reporting.

6.4. Efficacy

6.4.1. Parasite Count

Parasite counts will be done at the times indicated in Table 3 below, for adults and children. 10ul venous blood, or a thumbprick sample will be taken. At each occasion when parasites are to be looked for and counted, two thick and two thin films will be made.

1. One thick film will be stained with Field's stain, and examined with the oil immersion lens. If there are less than 10 asexual parasites in the average field of view, this slide will be used to count parasites. Both parasites and white blood cells will be counted in successive fields until >200 wbcs have been counted. Using the total blood wbc/ul obtained from the most recent Coulter Counter reading, the parasites/ul will be calculated.
2. If the thick film contains >10 asexual parasites/ul, a thin film will be stained by the reverse Field's stain, and parasites counted in a minimum of 500 red blood cells (rbc). The parasites/ul will then be calculated from the whole blood rbc/ul count obtained in the most recent Coulter counter reading.
3. The unused films will be fixed and stored for future use if required.

All slides will be read in the Wellcome Trust Laboratory, where a QC procedure is in place, involving checking every tenth slide and eliciting an additional expert reading in the event of significant discrepancies.

Screening parasitaemia will be calculated using the nominal WBC count value of 8,000ul^{-1}, according to WHO protocol. The *actual* parasitaemia will be corrected for the WBC count for each subject once the haematology results are available. The corrected parasitaemia

value will be used as the baseline level for PC90, PC50, PC99 calculations and assessment of treatment failure.

Should an adult's *actual* parasitaemia at time 0 be below 5,000ul^{-1}, they will be excluded from the per-protocol population. Similarly, should a child's *actual* parasitaemia at time 0 be below 12,500ul^{-1}, they will be excluded from the per-protocol population.

Table 3 parasite count times

Parasite Count	Day 0									Day 1				Day 2	Day 3	Day 7	Day 14
Time after dosing (hours)	0	1	2	3	4	6	8	12	18	24	30	36	42	48	72		
Adults	√	√	√	√	√	√	√	√	√	√	√	√	√	√	√	√	√
Children	√					√		√	√	√		√	√	√	√	√	√

6.4.2. Parasite Viability

Parasite viability measurement [Watkins, 1993; Murphy 1995] may be the most sensitive tool available to detect differences between the different artesunate doses. The initial parasite kill rate is sensitively dependent on the ART concentration in the parasitised RBC. Although it is possible to measure the rate of parasite disappearance from the peripheral circulation, and we are planning to do this by frequent microscopy after the start of therapy, measuring the proportion of the parasitaemia which is alive [or dead] may be better in terms of deciding equivalence of ART dose.

Method

Small venous blood samples (100ul) will be taken at the times indicated Table 4 below, and put into *in vitro* culture. Asexual reproduction is arrested at metaphase by including aphidicolin in the culture medium - all viable parasites thus end up as fat trophozoites: any dead parasites remain as rings. Because it is easy to microscopically define rings and trophozoites, it is possible to determine the proportion of parasites that were viable at the time the sample was taken.

Table 4 Parasite Viability sample times

Parasite Viability	Time after dosing (hours)						
	0	1	2	4	8	12	24
ADULTS	√	√	√	√	√	√	√
CHILDREN	√					√	

6.4.3. Treatment failure

A subject will be considered a treatment failure if they experience either early or late treatment failure based on the following definitions:

Early treatment failure

- The development of severe malaria syndrome or parasite density > 250,000/ul at any time on days 0, 1 , 2 or 3
- Evidence between 48 and 72 hours of the trial (day 2) that the subject is symptomatic **and** has a parasitaemia greater than the value recorded at entry to the trial.
- Parasitaemia between 72 and 96 hours of the trial (day 3) greater than 25% of the value recorded on entry to the trial.

Late treatment failure

- The need for admission to hospital for the treatment of parasitaemic illness between days 4 and 14 inclusive when: (a) the subject has not previously met the criteria for early treatment failure, and (b) PCR analysis of markers including MSP1 suggest that the parasites are unlikely to be a new infection.
- Evidence that the subject is symptomatic and any level of *P. falciparum* parasitaemia on any day between 4 and 6.
- Any level of *P.falciparum* parasitaemia on any day between day 7 and 14 inclusive, where subsequent PCR analysis of markers including MSP1 suggest that the parasites are unlikely to be a new infection.

The WHO definition of late treatment failure has been amended to include planned PCR work on markers including MSP1. The following addition has been made to the definition for late treatment failure:

- Development of severe anaemia (haemoglobin lower than 5 g%) in the presence or absence of parasitaemia on days 4 to 14, inclusive.

6.4.4. PCR analysis

PCR filter papers will be prepared on day 0, then day 7 or 14 if parasites are detected, or on any unscheduled visit where the subject is thought to fulfil the criteria for late treatment failure described in section 6.4.3. Two drops (approx 50ul) of blood will be collected on pre-printed filter paper and allowed to dry. Once dry samples will be placed in small plastic bags and stored at $4^{o}C$ until analysis.

6.4.5. Temperature

Temperature will be recorded in all subjects at the times given in the Table 5 :

Table 5 Temperature sample times

Temperature	Day 0							Day 1				Day 2	Day 3	Day 7	Day 14
Time after dosing (hours)	0	2	4	6	8	12	18	24	30	36	42	48	72		
Adults	√	√	√	√	√	√	√	√	√	√	√	√	√	√	√
Children	√			√		√	√	√	√	√	√	√	√	√	√

6.4.6. Gametocyte analysis

Slides prepared for parasitology will be read for the presence or absence of gametocytes per 200 white blood cells, on days 0 (pre-dose), 3, 7 and 14. If gametocytes are present, their density will be calculated and recorded as the number of gametocytes present per ul.

6.5. Pharmacokinetics

Pharmacokinetic sampling will only be conducted in adults, as sampling from children would require the drawing of too much venous blood. PK data is a key secondary objective in this study and so important to conduct in adults. Times of blood sampling for PK analysis in adults is given below.

Table 6 PK sampling times

ADULTS	Day 0																Day 1	Day 2	Day 3
Time after Dosing (hr)	0	15 min	30 min	1	1.5	2	2.5	3	3.5	4	5	6	8	10	12	18	24	48	72
PK sample	√	√	√	√	√	√	√	√	√	√	√	√	√	√	√	√	√	√	√

Method for handling PK blood samples

Blood, approx 2ml, will be collected into polypropylene tubes containing lithium heparin, mixed gently and placed on crushed wet-ice for no longer than 30mins, until centrifugation at 1800g for 10 minutes using a refrigerated centrifuge. The resultant plasma will be separated, and transferred in equal volumes to two uniquely labelled clear polypropylene tubes and frozen immediately over solid carbon dioxide or in a freezer at nominal –80°C. Samples will be transported frozen to WW Bioanalysis, DMPK, Ware,

to be stored at approximately -80°C until analysed. Plasma concentrations of CPG, CCP, DDS, MADDS, ART and DHA will be determined using currently approved methods by the WW Bioanalysis group, Ware and be used to evaluate T_{max}, C_{max}, $t_{1/2}$, AUC_{24} and AUC_{∞} if data permit. Additionally, the effect of artesunate on the pharmacokinetics of the components of Lapdap will be assessed, if data permit.

7. INVESTIGATIONAL PRODUCT(S)

7.1. Description of Investigational Product

LAPDAP 15/18.75mg tablets are white to off-white capsule shaped tablets with a break line on one side and marked CD15 on the other. **LAPDAP** 80/100 tablets are dark pink capsule shaped tablets, with a break line on one side, and the other is marked CD80.

Artesunate will be provided as off white tablets containing either 1mg, 10mg, 25mg or 50 mg artesunate.

All products will be manufactured in accordance with Good Manufacturing Practice.

7.2. Dosage and Administration

Subjects will receive the nominal target doses of 2.0mg/kg chlorproguanil and 2.5mg/kg dapsone (See Appendix 5: Dosing charts). Subjects will receive half, one, one and a half or 2 tablets (15/18.75mg or 80/100mg tablets) daily for three days (See Appendix 5: Dosing charts).

Subjects will be randomised to the nominal target dose of artesunate of 0, 1, 2 or 4mg/kg. Subjects will receive a combination of 1mg, 10mg, 25mg or 50mg tablets daily for 3 days (See Appendix 5: Dosing charts) . Subjects randomised to **LAPDAP** alone will receive no artesunate.

Subjects will take the study drugs as tablets, which may be swallowed whole or chewed.

All doses of study drug will be taken under supervision for 1/2 hour. Vomiting within 1/2 hour of the dose will lead to re-dosing; vomiting within 1/2 hour of the re-dose will lead to withdrawal. Wherever possible dosing will be at the same time of day.

7.3. Dose Rationale

The dose of **LAPDAP** for the treatment of acute uncomplicated falciparum malaria has been estabished as 2mg/kg CPG, 2.5mg/kg DDS through the **LAPDAP** development programme. The most widely used dose of artesunate is 4mg/kg, in combination with other anti-malarials, although there is limited data to support this choice of dose. The purpose of this study is to determine which dose of artesunate to combine with the established dose of **LAPDAP** in the final combination tablet. The maximum dose to be tested is 4mg/kg, the minimum 1mg/kg where a ‘no-effect’ level may be observed. The

decision matrix for establishing the final dose of artesunate to be combined with the established dose of **LAPDAP** is given in section 11.7.2

7.4. Blinding

This will be an open-label study with all subjects receiving active **LAPDAP** and, in addition, randomised to 0, 1 , 2 or 4mg/kg artesunate. Once the informed consent has been obtained, each subject will be allocated a unique study CRF number and randomisation number, stratified by gender (adults) or age (children). Due to the complexity of the dosing schedule to achieve acurate mg/kg artesunate doses, this will be an open-label study. However the technicians performing the slide reading and viability analysis will be blinded to the treatment group each subject is assigned to.

7.5. Treatment Assignment

Subjects will be assigned to study treatment in accordance with the randomisation schedule, which will be provided by GSK. Adult subjects will be stratified by gender, in a ratio of 3:2, men:women in each treatment arm. Children will be randomised according to their age; split into 5 strata; 1-<2 years, 2-<3years, 3-<4years, 4-<5years and 5years to 10 years. The distribution of patients is shown in tables 7 and 8 below:

Table 7 Stratification of children by age, by dose of ART

**Age** (years)	Dose (mg/kg ART) plus fixed dose **LAPDAP**			
	**0**	**1**	**2**	**4**
1-2	6	6	6	6
2-3	6	6	6	6
3-4	6	6	6	6
4-5	6	6	6	6
5-10	6	6	6	6
**TOTAL**	**30**	**30**	**30**	**30**

Table 8 Stratification of adults by gender, by dose of ART

**Gender**	Dose (mg/kg ART) plus fixed dose **LAPDAP**			
	**0**	**1**	**2**	**4**
Male	18	18	18	18
Female	12	12	12	12
**TOTAL**	**30**	**30**	**30**	**30**

7.6. Packaging and Labelling

All study medication will be supplied as labelled containers of bulk drug product to be dispensed in accordance with the stratified randomisation and subject's weight, as detailed in Appendix 5: Dosing charts.

The contents of the label will be in accordance with all applicable regulatory requirements, but will contain at least the protocol number, dosing instructions, storage instructions, expiry date and sponsor.

7.7. Preparation

No preparation is required. For small children it may be necessary to crush the tablets on a spoon with a little water.

7.8. Handling and Storage

Investigational product must be dispensed or administered according to procedures described herein. Only subjects enrolled in the study may receive investigational product, in accordance with all applicable regulatory requirements. Only authorised site staff may supply or administer investigational product. All investigational products must be stored in a secure area with access limited to the investigator and authorised site staff and under physical conditions that are consistent with investigational product-specific requirements.

All medication must be stored in a locked cabinet, at or below 30°C.

7.9. Product Accountability

The investigator is responsible for investigational product accountability, reconciliation, and record maintenance. In accordance with all applicable regulatory requirements, the investigator or designated site staff must maintain investigational product accountability records throughout the course of the study. This person(s) will document the amount of investigational product received from GSK, the amount supplied and/or administered to and returned by subjects, if applicable.

7.10. Assessment of Compliance

Treatment will be administered in the presence of the Investigator or study nurse, and ingestion confirmed. This will be recorded in the CRF together with the time and date of dosing.

7.11. Treatment of Investigational Product Overdose

There is no experience of acute overdosage with **LAPDAP.** This precludes characterisation of sequelae and assessment of antidotal efficacy at this time. Since chlorproguanil/dapsone has been shown to induce methaemoglobinaemia in clinical trials, clinically significant levels may be encountered in overdose. Methaemoglobinaemia greater than 15-20% is associated with dyspnoea, and cyanosis. Haemolysis and anaemia may also be observed in susceptible individuals. Cyanosis due to methaemoglobin formation may not immediately be apparent.

In case of accidental over-dosage, immediate induction of emesis and/or gastric lavage is recommended, in conjunction with appropriate supportive measures particularly to reduce methaemoglobinaemia, and to correct anaemia.

In case of artesunate overdosage, symtoms should be treated as required.

7.12. Occupational Safety

Investigational product is not expected to pose significant occupational safety risk to site staff under normal conditions of use and administration. A Material Safety Data Sheet (MSDS) describing occupational hazards and recommended handling precautions either will be provided to the investigator, where this is required by local laws, or is available upon request from GSK.

8. CONCOMITANT MEDICATIONS AND NON-DRUG THERAPIES

8.1. Permitted Medications

All concomitant medications taken during the study will be recorded in the CRF with indication, dose information, and dates of administration.

All subjects can be given paracetamol or codeine as an analgesic during the study, at the discretion of the treating physician. Allowable antibiotics are penicillin, cephalosporins and aminoglycocides.

8.2. Prohibited Medications

Any antimalarial, or antibiotic with antimalarial activity (erythromycin and other macrolides, co-trimoxazole and other sulphonamides, any tetracycline (including doxycycline, and quinolones.

Chloroquine: Prior treatment with chloroquine has been added to exclusion criteria 5, based on its use as first-line therapy in The Gambia, in protocol amendment no.4. This will apply to both the Malawi and Gambia Sites. Intercurrent use of chloroquine during the study period will be a protocol violation and cause exclusion from the analysis of the per-protocol group.

9. SUBJECT COMPLETION AND WITHDRAWAL

9.1. Subject Completion

Subjects will be followed for 14 days after enrolment. Active follow-up will be on days 0, 1, 2, 3, 7 and 14; all subjects will be seen on any day between 0 and 14 upon request.

Subjects will be described as completing the study if they satisfied all study entry criteria, have received the treatment and have attended all visits prescribed by the protocol and provided blood samples as defined for these visits.

9.2. Subject Withdrawal

A withdrawal is any subject who enters the study (i.e., gives informed consent), but does not complete the study (whether or not the subject received study medication).

9.2.1. Subject Withdrawal from Study

When a subject is withdrawn, the investigator should carry out all the assessments that would have been carried out at the next scheduled visit (unless the subject is lost to follow-up). The Study Conclusion Page of the CRF must be completed and the study medication records should be brought up to date as far as possible.

Subjects withdrawing from the study should attend for a follow-up visit three weeks after withdrawal for the monitoring of adverse events and changes in concomitant medication. Every effort will be made to follow-up subjects who withdrew due to drug related adverse events in order to determine the final outcome. This must then be recorded in the CRF and reported to GSK.

Subjects may be withdrawn from the study for any one of the following reasons:

- Withdrawal of consent at any stage
- Achievement of an end-point (early or late treatment failure, adverse event)
- Protocol violation

Subject Replacement

Subjects who are withdrawn (for whatever reason) during the study will be not be replaced.

9.2.2. Subject Withdrawal from Investigational Product

Subjects who withdraw from taking the investigational product should be followed-up to monitor safety up until day 14 if possible, i.e. not lost to follow-up.

10. ADVERSE EVENTS (AE) AND SERIOUS ADVERSE EVENTS (SAE)

The investigator is responsible for the detection and documentation of events meeting the criteria and definition of an AE or SAE as provided in this protocol. During the study, when there is a safety evaluation, the investigator or site staff will be responsible for detecting AEs and SAEs, as detailed in this section of the protocol.

10.1. Definition of an AE

Any untoward medical occurrence in a subject or clinical investigation subject, temporally associated with the use of a medicinal product, whether or not considered related to the medicinal product.

An AE can therefore be any unfavorable and unintended sign (including an abnormal laboratory finding), symptom, or disease (new or exacerbated) temporally associated with the use of a medicinal product.

Examples of an AE **includes**:

- Significant or unexpected worsening or exacerbation of the condition/indication under study. See Section 10.3., "Lack of Efficacy", for additional information.
- Exacerbation of a chronic or intermittent pre-existing condition including either an increase in frequency and/or intensity of the condition.
- New conditions detected or diagnosed after investigational product administration even though it may have been present prior to the start of the study.
- Signs, symptoms, or the clinical sequelae of a suspected interaction.
- Signs, symptoms, or the clinical sequelae of a suspected overdose of either investigational product or a concurrent medication (overdose per se should not be reported as an AE/SAE).
- Significant failure of expected pharmacological or biological action. See Section 10.3., "Lack of Efficacy" for additional information.

Examples of an AE **does not include** a/an:

- Medical or surgical procedure (e.g., endoscopy, appendectomy); the condition that leads to the procedure is an AE.
- Situations where an untoward medical occurrence did not occur (social and/or convenience admission to a hospital).
- Anticipated day-to-day fluctuations of pre-existing disease(s) or condition(s) present or detected at the start of the study that do not worsen.
- The disease/disorder being studied, or expected progression, signs, or symptoms of the disease/disorder being studied, unless more severe than expected for the subject's condition.

For GSK clinical studies, AEs may include pre- or post-treatment events that occur as a result of protocol-mandated procedures (i.e., invasive procedures, modification of subject's previous therapeutic regimen).

10.2. Definition of a SAE

A serious adverse event is any untoward medical occurrence that, at any dose:

a results in death.

b is life-threatening.

NOTE: The term 'life-threatening' in the definition of 'serious' refers to an event in which the subject was at risk of death at the time of the event. It does not refer to an event, which hypothetically might have caused death, if it were more severe.

c requires hospitalisation or prolongation of existing hospitalisation.

NOTE: In general, hospitalisation signifies that the subject has been detained (usually involving at least an overnight stay) at the hospital or emergency ward for observation and/or treatment that would not have been appropriate in the physician's office or out-patient setting. Complications that occur during hospitalisation are AEs. If a complication prolongs hospitalisation or fulfills any other serious criteria, the event is serious. When in doubt as to whether "hospitalisation" occurred or was necessary, the AE should be considered serious.

Hospitalisation for elective treatment of a pre-existing condition that did not worsen from baseline is not considered an AE.

d results in disability/incapacity, or

NOTE: The term disability means a substantial disruption of a person's ability to conduct normal life functions. This definition is not intended to include experiences of relatively minor medical significance such as uncomplicated headache, nausea, vomiting, diarrhoea, influenza, and accidental trauma (e.g. sprained ankle) which may interfere or prevent everyday life functions but do not constitute a substantial disruption.

e is a congenital anomaly/birth defect.

f Medical or scientific judgement should be exercised in deciding whether reporting is appropriate in other situations, such as important medical events that may not be immediately life-threatening or result in death or hospitalisation but may jeopardise the subject or may require medical or surgical intervention to prevent one of the other outcomes listed in the above definition. These should also be considered serious. Examples of such events are invasive or malignant cancers, intensive treatment in an emergency room or at home for allergic bronchospasm, blood dyscrasias or convulsions that do not result in hospitalisation, or development of drug dependency or drug abuse.

10.2.1. Disease-Related Events or Outcomes Not Qualifying as SAEs

None.

10.3. Lack of Efficacy

"Lack of efficacy" per se will not be reported as an AE. The signs and symptoms or clinical sequelae resulting from lack of efficacy will be reported if they fulfil the AE or SAE definition (including clarifications).

10.4. Clinical Laboratory Abnormalities and Other Abnormal Assessments as AEs and SAEs

Abnormal laboratory findings (e.g., clinical chemistry, haematology, urinalysis) or other abnormal assessments that are judged by the investigator as **clinically significant** will be recorded as AEs or SAEs if they meet the definition of an AE, as defined in Section 10.1. ("Definition of an AE"), or SAE, as defined in Section 10.2. ("Definition of a SAE"). Clinically significant abnormal laboratory findings or other abnormal assessments that are detected during the study or are present at baseline and significantly worsen following the start of the study will be reported as AEs or SAEs. However, clinically significant abnormal laboratory findings or other abnormal assessments that are associated with the disease being studied, unless judged by the investigator as more severe than expected for the subject's condition, or that are present or detected at the start of the study and do not worsen, will **not** be reported as AEs or SAEs.

The investigator will exercise his or her medical and scientific judgement in deciding whether an abnormal laboratory finding or other abnormal assessment is clinically significant.

10.5. Time Period, Frequency, and Method of Detecting AEs and SAEs

Adverse Events and SAEs will be recorded from the time of informed consent to end of follow-up.

10.6. Recording of AEs and SAEs

When an AE/SAE occurs, it is the responsibility of the investigator to review all documentation (e.g., hospital progress notes, laboratory, and diagnostics reports) relative to the event. The investigator will then record all relevant information regarding an AE/SAE on the CRF. It is not acceptable for the investigator to send photocopies of the subject's medical records to GSK in lieu of completion of the appropriate AE/SAE CRF pages. However, there may be instances when copies of medical records for certain cases are requested by GSK. In this instance, all subject identifiers will be blinded on the copies of the medical records prior to submission to GSK.

The investigator will attempt to establish a diagnosis of the event based on signs, symptoms, and/or other clinical information. In such cases, the diagnosis should be documented as the AE/SAE and not the individual signs/symptoms.

10.7. Evaluating AEs and SAEs

10.7.1. Assessment of Intensity

The investigator will make an assessment of intensity for each AE and SAE reported during the study. The assessment will be based on the investigator's clinical judgement. The intensity of each AE and SAE recorded in the CRF should be assigned to one of the following categories:

Mild: An event that is easily tolerated by the subject, causing minimal discomfort and not interfering with everyday activities.

Moderate: An event that is sufficiently discomforting to interfere with normal everyday activities.

Severe: An event that prevents normal everyday activities.

An AE that is assessed as severe should not be confused with a SAE. Severity is a category utilised for rating the intensity of an event; and both AEs and SAEs can be assessed as severe. An event is defined as 'serious' when it meets one of the pre-defined outcomes as described in Section 10.2., "Definition of a SAE".

10.7.2. Assessment of Causality

The investigator is obligated to assess the relationship between investigational product and the occurrence of each AE/SAE. The investigator will use clinical judgement to determine the relationship. Alternative causes, such as natural history of the underlying diseases, concomitant therapy, other risk factors, and the temporal relationship of the event to the investigational product will be considered and investigated. The investigator will also consult the CIB/IB and/or Product Information, for marketed products, in the determination of his/her assessment.

There may be situations when an SAE has occurred and the investigator has minimal information to include in the initial report to GSK. However, it is very important that the investigator always make an assessment of causality for every event prior to transmission of the SAE CRF to GSK. The investigator may change his/her opinion of causality in light of follow-up information, amending the SAE CRF accordingly. The causality assessment is one of the criteria used when determining regulatory reporting requirements.

The investigator will provide the assessment of causality as per instructions on the SAE form in the CRF.

10.8. Follow-Up of AEs and SAEs

After the initial AE/SAE report, the investigator is required to proactively follow each subject and provide further information to GSK on the subject's condition.

All AEs and SAEs documented at a previous visit/contact and are designated as ongoing, will be reviewed at subsequent visits/contacts.

All AEs and SAEs will be followed until resolution, until the condition stabilises, until the event is otherwise explained, or until the subject is lost to follow-up. Once resolved, the appropriate AE/SAE CRF page(s) will be updated. The investigator will ensure that follow-up includes any supplemental investigations as may be indicated to elucidate the nature and/or causality of the AE or SAE. This may include additional laboratory tests or investigations, histopathological examinations, or consultation with other health care professionals.

GSK may request that the investigator perform or arrange for the conduct of supplemental measurements and/or evaluations to elucidate as fully as possible the nature and/or causality of the AE or SAE. The investigator is obligated to assist. If a subject dies during participation in the study or during a recognised follow-up period, GSK will be provided with a copy of any post-mortem findings, including histopathology.

New or updated information will be recorded on the originally completed “SAE” CRF, with all changes signed and dated by the investigator. The updated SAE CRF should be resent to GSK within the time frames outlined in Section 10.9.

10.9. Prompt Reporting of SAEs to GSK

SAEs will be reported promptly to GSK as described in the following table once the investigator determines that the event meets the protocol definition of an SAE.

10.9.1. Timeframes for Submitting SAE Reports to GSK

	Initial SAE Reports		Follow-up Information on a Previously Reported SAE	
Type of SAE	Time Frame	Documents	Time Frame	Documents
All SAEs	24 hrs	"SAE" CRF pages	24 hrs	Updated "SAE" CRF pages

10.9.2. Completion and Transmission of the SAE Reports

Once an investigator becomes aware that an SAE has occurred in a study subject, she/he will report the information to GSK within 24 hours as outlined in Section 10.9., “Prompt Reporting of SAEs to GSK”. The SAE CRF will always be completed as thoroughly as possible with all available details of the event, signed by the investigator (or designee), and forwarded to GSK within the designated time frames. If the investigator does not have all information regarding an SAE, he/she will not wait to receive additional information before notifying GSK of the event and completing the form. The form will be updated when additional information is received.

The investigator will always provide an assessment of causality at the time of the initial report as described in Section 10.7.2., “Assessment of Causality”.

Facsimile transmission of the “SAE” CRF is the preferred method to transmit this information to the project contact for SAE receipt. In rare circumstances and in the absence of facsimile equipment, notification by telephone is acceptable, with a copy of the "SAE" CRF sent by overnight mail. Initial notification via the telephone does not replace the need for the investigator to complete and sign the SAE CRF within the time frames outlined in Section 10.9., “Prompt Reporting of SAEs to GSK”.

GSK will provide a list of project contacts for SAE receipt, fax numbers, telephone numbers, and mailing addresses.

The following pages of the CRF must accompany the SAE forms that are forwarded to GSK: “Demography”, “Medical History”, “Concomitant Medications”, “Study Medication Records”, and “Form D” (if applicable).

10.10. Regulatory Reporting Requirements For SAEs

The investigator will promptly report all SAEs to GSK in accordance with the procedures detailed in Section 10.9., "Prompt Reporting of SAEs to GSK." GSK has a legal responsibility to notify, as appropriate, both the local regulatory authority and other regulatory agencies about the safety of a product under clinical investigation. Prompt notification of SAEs by the investigator to the appropriate project contact for SAE receipt is essential so that legal obligations and ethical responsibilities towards the safety of other subjects are met.

The investigator, or responsible person according to local requirements, will comply with the applicable local regulatory requirements related to the reporting of SAEs to regulatory authorities and the Institutional Review Board (IRB)/Independent Ethics Committee (IEC).

Expedited Investigator Safety Reports (EISR) are prepared according to GSK policy and are forwarded to investigators as necessary. An EISR is prepared for a SAE that is both attributable to investigational product and unexpected. The purpose of the EISR is to fulfil specific regulatory and Good Clinical Practice (GCP) requirements, regarding the product under investigation.

An investigator who receives an EISR describing a SAE or other specific safety information from GSK will file it with the Investigator Brochure and will notify the IRB or IEC, if appropriate according to local requirements.

10.11. Post-study AEs and SAEs

A post-study AE/SAE is defined as any event that occurs outside of the AE/SAE detection period defined in Section 10.5., “Time Period, Frequency, and Method of Detecting AEs and SAEs”, of the protocol.

Investigators are not obligated to actively seek AEs or SAEs in former study participants. However, if the investigator learns of any SAE, including a death, at any time after a

subject has been discharged from the study, and he/she considers the event reasonably related to the investigational product, the investigator will promptly notify GSK.

10.12. SAEs Related to Study Participation

An SAE considered related to study participation (e.g., procedures, invasive tests, a change in existing therapy), even if it occurs during the pre- or post-treatment period, will be reported promptly to GSK (see Section 10.9., "Prompt Reporting of SAEs to GSK").

11. DATA ANALYSIS AND STATISTICAL CONSIDERATIONS

11.1. Hypotheses

The **null hypothesis** for this study is:

There is no difference between **LAPDAP** administered alone and **LAPDAP** administered in conjunction with artesunate in PC90 in the per-protocol population.

The **alternative hypothesis** for this study is:

There is a difference between **LAPDAP** administered alone and **LAPDAP** administered in conjunction with artesunate in PC90 in the per-protocol population.

If there is sufficient evidence against, the null hypothesis will be rejected in favour of the alternative. It will be tested using a closed testing procedure (to establish the dosing level of artesunate) using a two-sided significance test and a 5% significance level.

11.2. Treatment Comparisons of Interest

11.2.1. Primary Comparisons of Interest

The primary comparison is the PC90 for the PP population, in adults only. PC90 is the time to achieve a reduction of the parasitaemia to 90% of baseline. As efficacy with **LAPDAP** alone is 96%, we would not be able to detect a difference in clinical efficacy between the control (**LAPDAP** alone), and the addition of artesunate. Parasite clearance is a good indicator of the activity of artesunate, as artesunate is known to rapidly reduce the parasite load[11]. Differences between the addition of 1, 2, or 4 mg/kg of artesunate to the fixed dose of **LAPDAP** will be investigated.

Each of the three subject groups treated with **LAPDAP** and artesunate (Groups B-D) will be compared with Group A (**LAPDAP** alone).

A step-down procedure will be used to compare each artesunate Group (B-D) with Group A. Only if the preceding comparison is statistically significant at the 5% level will the next one be tested. This preserves the overall type I level at 0.05.

a Group D (**LAPDAP** + 4 mg/kg artesunate) vs. Group A (**LAPDAP** alone)

b Group C (**LAPDAP** + 2 mg/kg artesunate) vs. Group A (**LAPDAP** alone)

c Group B (**LAPDAP** + 1 mg/kg artesunate) vs. Group A (**LAPDAP** alone)

11.2.2. Other comparisons of interest

In addition to the above comparisons, a linear trend of artesunate dose will be investigated.

The comparisons detailed in 11.2.1 will be made, using the closed testing procedure for the following secondary efficacy endpoints: PC50, PC90, parasite viability and early/late treatment failure. Details of these analyses are given in section 11.7.2. These will be presented for the ITT and PP populations.

The presence or absence of gametocytes (% of gametocytaemic subjects) will be summarised by artesunate dose. Additionally, summary statistics will be presented for gametocyte density by artesunate dose.

Reduction in temperature will be summarised by artesunate dose.

11.3. Interim Analysis

No interim analyses are planned.

11.4. Sample Size Considerations

The sample size is calculated on the number of adults required for the PC90 primary endpoint. An equal number of children will also be recruited.

One hundred and twenty adult men and women between 18 and 45 years and 120 children between 12 months and 120 months, who present at a health care facility with symptoms of uncomplicated malaria which is then diagnosed by parasitology, will be recruited.

11.4.1. Sample Size Assumptions

A total of 88 evaluable subjects (22 subjects in each group) will be required in order to provide 90% power at the two-sided 5% level to detect a difference in mean PC90 of 9. This is based on the findings of Angus [Angus, 2002] in their study of artesunate in combination with mefloquine. In order to achieve 88 evaluable subjects, it will be necessary to randomise 120 subjects (30 subjects per treatment arm); assuming that approximately 25% of subjects will drop out during the course of the study.

11.4.2. Sample Size Sensitivity

The sample size calculation assumes a common standard deviation of 9 and the statistical test performed will be a two group t-test. If the common standard deviation was 10.5,

then the proposed sample size of 22 evaluable subjects per treatment arm would provide 80% power to detect a difference in mean PC90 of 9.

11.4.3. Sample Size Re-estimation

There is no sample re-estimation planned for this study.

11.5. Analysis Populations

The following three populations will be evaluated:

Intent-to-treat population

All subjects who were randomised and have received any dose of the study medication, irrespective of whether they vomited, will be included in the intent-to-treat population. The intent-to-treat analysis of efficacy will be used to confirm findings from the primary efficacy analysis and will be the population used for safety analysis.

Per-protocol population

The per-protocol population will be used as the primary population for efficacy analyses. Subjects will be eligible for the per-protocol efficacy analysis providing the criteria in the intent-to-treat population has been satisfied and the following apply:

- The subject has completed all visits as specified by the protocol
- No major protocol violation exists with regard to Inclusion/Exclusion criteria
- No prohibited concomitant medications were taken during the treatment period
- That an adult's actual parasitaemia level at time 0 was not below 5,000ul^{-1}, or
- That a child's actual parasitaemia level at time 0 was not below 12,500ul^{-1}

11.5.1. Data Sets

PC90 (and PC50/99) will be estimated from subject’s parasite count data. Only data collected within 15 minutes of timepoints up to and including 12hours, and within 1 hour of all remaining timepoints will be included in the statistical model.

11.6. General Considerations for Data Analysis

Gender will be included in the statistical model for the primary endpoint for adults and age will be included in the statistical model for children.

11.6.1. Withdrawal

Data from subjects who withdraw from the study will be used if available.

11.6.2. Missing Data

All analyses and summaries of endpoints will be performed with no replacement of missing efficacy and safety data.

11.6.3. Derived and Transformed Data

Each subject's value of PC50, PC90 and PC99 will be estimated from fitting a logistic curve to the change in parasite count over time. The parasite count at Day 0 will be scaled to the value of 100. PC90 will be the estimated time that the scaled variable of parasite count is equal to 10 (i.e. PC90 is defined as the decrease from 100 to 10 which equals 90). PC50 and PC99 will be similarly estimated.

It is expected that PC90 is log-normally distributed, therefore it is likely that these data will be log transformed. Similarly for PC50 and PC99.

The proportion of viable parasites will be calculated using the method described in section 6.4.2. The parasite viability at Day 0 will be scaled to the value of 100. The parasite viability at 12 hours post-first dose will calculated as a proportion of the day 0 value.

Early and late treatment failures will be defined in terms of the definition provided in section 6.4.3.

Reduction in temperature will be derived as the change in post-dose temperature from baseline (i.e. change = baseline-post dose).

11.6.4. Assessment Windows

These will be stated in the Reporting and Analysis Plan (RAP).

11.7. Efficacy Analyses

Summaries of the primary and secondary endpoints will be presented for both the intent-to-treat and per-protocol populations. The per-protocol population will be considered as the primary population for efficacy evaluations.

All summary statistics will be presented by the dose of artesunate received (i.e. Group A-D). Continuous data will be summarised by the mean, standard deviation, median, minimum and maximum. Categorical data will be summarised by the number and percentage of subjects.

Analyses will be performed separately for adults and children.

11.7.1. Primary Analysis

The primary analysis will be performed in adults. Analyses of covariance will be used to analyse the primary variable (PC90) adjusting for the stratifying variable (gender), centre and treatment. Log transformed data will be used if necessary.

Each comparison a) to c) in section 11.2.1 will be compared. Only if the preceding comparison is statistically significant will the next one will be tested. Estimates of the treatment difference, 95% confidence interval and p-value will be presented where appropriate.

The interaction between gender and dose, and between centre and dose will be investigated in secondary analyses, but will not be included in the closed testing procedure to determine the dose.

An appropriate non-parametric method (for example the Wilcoxon rank sum test) will be used if the assumptions for the parametric analysis are not met.

11.7.2. Secondary Analysis

A mixed effects model will be used to analyse the parasite count recorded post-dosing. Log transformed data will be used if necessary. Subject will be considered as a random variable (the interaction between subject and time will also be investigated). Terms for baseline parasite count, centre, gender in adults and age in children will be included in the model if significant at the 5% level. Treatment comparisons will be made in the same way as the primary efficacy endpoint. The results will also be shown graphically.

In addition the linear trend of artesunate dose will be investigated by including a term for artesunate dose as a continuous variable in the analyses of variance.

The presence or absence of gametocytes (% of gametocytaemic subjects) will be summarised by artesunate dose. Additionally, summary statistics will be presented for gametocyte density by artesunate dose. However no formal statistical comparisons will be made.

The proportion of treatment failures will be analysed using the chi-squared test. An estimate of the treatment difference will be presented along with an asymptotic 95% confidence interval.

Reduction in temperature will be summarised by artesunate dose.

All other efficacy parameters (including the key secondary variable, parasite viability) will be analysed in a similar way to PC90.

For each secondary endpoint, each comparison a) to c) in section 11.2.1 will be compared. Only if the preceding comparison is significant will the next one be tested. Estimates of the treatment difference, 95% confidence interval and p-value will be presented where appropriate.

Evaluation of children's data

The above describes the full evaluation being conducted in adults. In children more limited data will be available as it is not ethical to collect as many blood samples. It is therefore expected that the estimate of PC90 and other endpoints based on blood sampling will be less precise for children than adults. Therefore a separate analysis will be conducted for children (adjusting for the stratifying variable of age) and the confidence intervals will be compared visually between adults and children.

Decision matrix for selection of dose

The selection of the dose will be based upon the full analysis conducted from the rich adult data (as described above) and then the children's data will verify this selected dose for its appropriateness for children. The following decision matrix will be used to guide this process.

If 1, 2 and 4mg/kg of artesunate are better than 0, but results are similar, then 2mg/kg will be the selected dose.

If 1 and 2 mg/kg are better than 0, results are similar and 4mg/kg is better, then 4mg/kg will be selected.

If 1mg/kg is better than 0 but less effective than 2 & 4mg/kg (which show similar results) then 4mg/kg will be selected.

If 4mg/kg shows advantages over 0, 1 and 2mg/kg then 4mg/kg will be selected.

This process will be based upon the PC90 and parasite viability (PV) data. The PC90 results will be the main determinant, unless the PV data shows an important biological difference.

For example, based upon the work of Murphy [Murphy, 1995] we anticipate that Group A (**LAPDAP** alone) will have a PV of 93+7%, 12 hours after start of treatment. We anticipate groups C (2mg/kg ART) and D (4 mg/kg ART) would have a PV value of 15 +15%, 12 hours after the start of treatment. An important biological difference in viability would be an increase of the PV in any group to double the value anticipated in the 2 and 4mg/kg groups. In this case the higher dose would be selected.

It is intended that the analysis will proceed as described in the decision matrix, but a full clinical review of the safety data and gametocyte data will be conducted to ensure that no overriding signals are overlooked when deciding the dose. For example, if there are safety signals in the 4mg/kg artesunate group, but not in the 2mg/kg artesunate group, then the lower dose will be selected. Or, if gametocyte eradication is better in the 4mg/kg artesunate group than the 2mg/kg artesunate group, then the higher dose may be selected. Any deviation from the decision-matrix will be justified and documented in the Clinical Study Report.

When considering the children's data, in the case of a similar pattern of results then the dose selected from the adult data will remain. If the children's data differ from the adults data then the higher dose will be selected for both populations.

11.8. Safety Analyses

Safety analyses and summaries will be carried out on the ITT population. The incidence of drug-related adverse experiences that led to withdrawal and the development of clinically severe malaria (including a parasite count of > 250,000 ul^{-1}) will be presented in the same way as overall treatment failures.

Mean haemoglobin concentration on each follow-up day will be summarised by artesunate dose.

11.8.1. Extent of Exposure

The total number of tablets taken and the total exposure to study medication (mg) will be summarised by artesunate dose.

11.8.2. Adverse Events

All adverse events reported following enrolment of a subject into the study will be documented. Signs and symptoms (other than those of the disease under investigation) that occur prior to the first dose of study medication will be recorded and coded in MedDRA.

The incidence of each adverse event that is treatment emergent will be described. Treatment emergent adverse events are defined as adverse events that had an onset day on or after the day of the first dose of study medication. Adverse events which have missing onset dates will be considered to be treatment emergent. Information regarding concomitant medication will be presented as listings and tables.

11.8.3. Clinical Laboratory Evaluations

For the laboratory data, analyses will be conducted for data for Day 0, 3 (adults only), 7, and 14 as well as for any other visits, as appropriate.

11.9. Clinical Pharmacology Data Analyses

11.9.1. Pharmacokinetic Analyses

PK will only be analysed in adults. A noncompartmental extravascular model will be used to calculate the pharmacokinetic parameters for chlorproguanil, chlorcycloguanil, dapsone, mono acetyl dapsone, artesunate and dihydroartemisinin plasma levels. Plasma will be used to evaluate pharmacokinetic parameters for CPG, CCG, DDS, MADDS, ART and DHA, including: T_{max}, C_{max}, $t_{1/2}$, AUC_{24} and AUC_{∞} if data permit. Additional analyses

of the effect of artesunate on the pharmacokinetics of the components of Lapdap may be conducted, if data permit.

12. STUDY ADMINISTRATION

12.1. Regulatory and Ethical Considerations

12.1.1. Regulatory Authority Approval

GSK will obtain approval to conduct the study from the appropriate regulatory agency in accordance with any applicable country-specific regulatory requirements prior to a site initiating the study in that country.

12.1.2. Ethical Conduct of the Study and Ethics Approval

This study will be conducted in accordance with "good clinical practice" (GCP) and all applicable regulatory requirements, including, where applicable, 1996 version of the Declaration of Helsinki.

The investigator (or sponsor, where applicable) is responsible for ensuring that this protocol, the site's informed consent form, and any other information that will be presented to potential subjects (e.g., advertisements or information that supports or supplements the informed consent) are reviewed and approved by the appropriate IEC/IRB. The investigator agrees to allow the IEC/IRB direct access to all relevant documents. The IEC/IRB must be constituted in accordance with all applicable regulatory requirements. GSK will provide the investigator with relevant document(s)/data that are needed for IEC/IRB review and approval of the study. Before investigational product(s) and CRFs can be shipped to the site, GSK must receive copies of the IEC/IRB approval, the approved informed consent form, and any other information that the IEC/IRB has approved for presentation to potential subjects.

If the protocol, the informed consent form, or any other information that the IEC/IRB has approved for presentation to potential subjects is amended during the study, the investigator is responsible for ensuring the IEC/IRB reviews and approves, where applicable, these amended documents. The investigator must follow all applicable regulatory requirements pertaining to the use of an amended informed consent form including obtaining IEC/IRB approval of the amended form before new subjects consent to take part in the study using this version of the form. Copies of the IEC/IRB approval of the amended informed consent form/other information and the approved amended informed consent form/other information must be forwarded to GSK promptly.

12.1.3. Informed Consent

Informed consent will be obtained before the subject can participate in the study. The contents and process of obtaining informed consent will be in accordance with all applicable regulatory requirements.

12.1.4. Investigator Reporting Requirements

As indicated in Section 10.10, the investigator (or sponsor, where applicable) is responsible for reporting SAEs to the IEC/IRB, in accordance with all applicable regulations. Furthermore, the investigator may be required to provide periodic safety updates on the conduct of the study at his or her site and notification of study closure to the IEC/IRB. Such periodic safety updates and notifications are the responsibility of the investigator and not of GSK.

12.2. Study Monitoring

In accordance with applicable regulations, GCP, and GSK procedures, GSK monitors will contact the site prior to the subject enrolment to review the protocol and data collection procedures with site staff. In addition, the monitor will periodically contact the site, including conducting on-site visits. The extent, nature and frequency of on-site visits will be based on such considerations as the study objective and/or endpoints, the purpose of the study, study design complexity, and enrolment rate.

During these contacts, the monitor will:

- Check the progress of the study.
- Review study data collected.
- Conduct source document verification.
- Identify any issues and address their resolution.

This will be done in order to verify that the:

- Data are authentic, accurate, and complete.
- Safety and rights of subjects are being protected.
- Study is conducted in accordance with the currently approved protocol (and any amendments), GCP, and all applicable regulatory requirements.

The investigator agrees to allow the monitor direct access to all relevant documents and to allocate his/her time and the time of his/her staff to the monitor to discuss findings and any relevant issues.

At study closure, monitors will also conduct all activities described in Section 12.4, "Study and Site Closure."

12.3. Quality Assurance

To ensure compliance with GCP and all applicable regulatory requirements, GSK may conduct a quality assurance audit. Regulatory agencies may also conduct a regulatory inspection of this study. Such audits/inspections can occur at any time during or after completion of the study. If an audit or inspection occurs, the investigator and institution agree to allow the auditor/inspector direct access to all relevant documents and to allocate

his/her time and the time of his/her staff to the auditor/inspector to discuss findings and any relevant issues.

12.4. Study and Site Closure

Upon completion of the study, the monitor will conduct the following activities in conjunction with the investigator or site staff, as appropriate:

- Return of all study data to GSK.
- Data queries.
- Accountability, reconciliation, and arrangements for unused investigational product(s).
- Review of site study records for completeness.
- Shipment of PK samples to assay laboratory, as detailed in section 6.5

In addition, GSK reserves the right to temporarily suspend or prematurely discontinue this study either at a single site or at all sites at any time for reasons including, but are not limited to, safety or ethical issues or severe non-compliance. If GSK determines such action is needed, GSK will discuss this with the Investigator (including the reasons for taking such action) at that time. When feasible, GSK will provide advance notification to the investigator of the impending action prior to it taking effect.

GSK will promptly inform all other investigators and/or institutions conducting the study if the study is suspended or terminated for safety reasons, and will also inform the regulatory authorities of the suspension or termination of the study and the reason(s) for the action. If required by applicable regulations, the investigator must inform the IEC/IRB promptly and provide the reason for the suspension or termination.

If the study is prematurely discontinued, all study data must be returned to GSK. In addition, arrangements will be made for all unused investigational product(s) in accordance with the applicable GSK procedures for the study.

Financial compensation to investigators and/or institutions will be in accordance with the agreement established between the investigator and GSK.

12.5. Records Retention

Following closure of the study, the investigator must maintain all site study records in a safe and secure location. The records must be maintained to allow easy and timely retrieval, when needed (e.g., audit or inspection), and, whenever feasible, to allow any subsequent review of data in conjunction with assessment of the facility, supporting systems, and staff. Where permitted by local laws/regulations or institutional policy, some or all of these records can be maintained in a format other than hard copy (e.g., microfiche, scanned, electronic); however, caution needs to be exercised before such action is taken. The investigator must assure that all reproductions are legible and are a true and accurate copy of the original, and meet accessibility and retrieval standards,

including re-generating a hard copy, if required. Furthermore, the investigator must ensure there is an acceptable back-up of these reproductions and that an acceptable quality control process exists for making these reproductions.

GSK will inform the investigator of the time period for retaining these records to comply with all applicable regulatory requirements. The minimum retention time will meet the strictest standard applicable to that site for the study, as dictated by any institutional requirements or local laws or regulations, or GSK standards/procedures; otherwise, the retention period will default to 15 years.

The investigator must notify GSK of any changes in the archival arrangements, including, but not limited to, the following: archival at an off-site facility, transfer of ownership of the records in the event the investigator leaves the site.

12.6. Provision of Study Results and Information to Investigators

When a clinical study report is completed, GSK will provide the major findings of the study to the investigator.

12.7. Information Disclosure and Inventions

Ownership:

All information provided by GSK and all data and information generated by the site as part of the study (other than a subject's medical records) are the sole property of GSK.

All rights, title, and interests in any inventions, know-how or other intellectual or industrial property rights which are conceived or reduced to practice by site staff during the course of or as a result of the study are the sole property of GSK, and are hereby assigned to GSK.

If a written contract for the conduct of the study which includes ownership provisions inconsistent with this statement is executed between GSK and the study site, that contract's ownership provisions shall apply rather than this statement.

Confidentiality:

All information provided by GSK and all data and information generated by the site as part of the study (other than a subject's medical records) will be kept confidential by the investigator and other site staff. This information and data will not be used by the investigator or other site personnel for any purpose other than conducting the study. These restrictions do not apply to: (1) information which becomes publicly available through no fault of the investigator or site staff; (2) information which it is necessary to disclose in confidence to an IEC or IRB solely for the evaluation of the study; (3) information which it is necessary to disclose in order to provide appropriate medical care to a study subject; or (4) study results which may be published as described in the next paragraph. If a written contract for the conduct of the study which includes

confidentiality provisions inconsistent with this statement is executed, that contract's confidentiality provisions shall apply rather than this statement.

Publication:

For multi-centre studies, the first publication or disclosure of study results shall be a complete, joint multi-centre- publication or disclosure co-ordinated by GSK. Thereafter, any secondary publications will reference the original publication(s).

Prior to submitting for publication, presentation, use for instructional purposes, or otherwise disclosing the study results generated by the site (collectively, a "Publication"), the investigator shall provide GSK with a copy of the proposed Publication and allow GSK a period of at least thirty (30) days [or, for abstracts, at least five (5) working days] to review the proposed Publication. Proposed Publications shall not include either GSK confidential information other than the study results or personal data on any subject, such as name or initials.

At GSK's request, the submission or other disclosure of a proposed Publication will be delayed a sufficient time to allow GSK to seek patent or similar protection of any inventions, know-how or other intellectual or industrial property rights disclosed in the proposed Publication.

If a written contract for the conduct of the study, which includes publication provisions inconsistent with this statement is executed, that contract's publication provisions shall apply rather than this statement.

12.8. Data Management

Subject data are collected by the investigator or designee using the Case Report Form (CRF) defined by GSK. Subject data necessary for analysis and reporting will be entered/transmitted into a validated database or data system. Clinical data management will be performed in accordance with applicable GSK standards and data cleaning procedures. Database freeze will occur when data management quality control procedures are completed. Original CRFs will be retained by GSK, while the investigator will retain a copy.

13. REFERENCES

Angus BJ, Thaiaporn I, Chanthapadith K, Suputtamongkol Y, White NJ. Oral artesunate dose-response relationship in acute falciparum malaria. Antimicrob Agents Chemother. 2002 Mar;46(3):778-82.

Anonymous (1994). Artemisinin. Trans. R. Soc. Trop. Med. Hyg. 88 (Suppl. 2) 1 - 65.

Murphy SA, Watkins WM, Bray P, Lowe B, Winstanley PA and Marsh K (1995) Parasite viability during treatment of severe falciparum malaria : differential effects of artemether and quinine. Am. J. Trop. Med. Hyg. 53; 303-305.

Nzila MA., Mberu EK, Sulo J, Dayo H, Winstanley PA, Sibley CH, Watkins WM (2000a). Towards an understanding of the mechanism of pyrimethamine/sulfadoxine resistance in *Plasmodium falciparum*: the genotyping of dihydrofolate reductase and dihydropteroate synthase of Kenyan parasites. Antimicrob. Agent Chemother. 44(4): 991-996.

Nzila AM, Nduati E, Mberu EK, Hopkins Sibley C, Monks SA, Winstanley PA, Watkins WM. (2000b) Molecular evidence of greater selective pressure for drug resistance exerted by the long-acting antifolate Pyrimethamine/Sulfadoxine compared with the shorter-acting Chlorproguanil/Dapsone on Kenyan Plasmodium falciparum. J Infect Dis. 2000 Jun;181(6):2023-2028.

Watkins WM, Woodrow C and Marsh K (1993) Falciparum malaria : differential effects of antimalarial drugs on ex vivo parasite viability during the critical early phase of therapy. Amer. J. Hyg. Trop. Med. 49; 106-112.

Watkins WM, Mberu EK, Winstanley PA and PloweC. (1997) The efficacy of antifolate antimalarial combinations in Africa : a predictive model based on pharmacodynamic and pharmacokinetic analyses. Parasitology Today 13; 459-464.

Watkins WM, Mberu EK, Winstanley PA, Plowe CV (1999). More on 'The efficacy of antifolate antimalarial combinations in Africa' Parasitology Today 15; 131-132

Wernsdorfer, W.H. and Payne, D. (1991). The dynamics of drug resistance in Plasmodium falciparum. Pharm. Ther, 50, 905 - 121.

White NJ, Nosten F, Looaresuwan S, Watkins WM, Marsh K, Snow RW, Kokwaro G, Ouma J, Hien TT, Molyneux M, Taylor T, Newbold CI, Ruebush TK, Danis M, Greenwood B, Anderson RM, Olliaro P. 'Averting a malaria disaster' Lancet (1999a), 353: 1965-1967.

White N, Nosten F, Hien TT, Watkins W, Olliaro P. 'Averting a malaria disaster' Lancet (1999b), 354: 1389- 1390.

World Health Organisation (2000). Severe falciparum malaria. *Transactions of the Royal Society of Tropical Medicine and Hygeine*, **94**, supplement 1.

World Health Organisation (2001). Antimalarial Drug Combination Therapy. *Report of a WHO Technical Consultation.*

WHO. Report of an RBM/TDR informal consultation of clinical investigators on the safety and efficacy with artemisinin compunds in pregnancy. (2002). July 22-23, Geneva

14. APPENDICES

14.1. Appendix 1: Adult Time and Events Table

Adults	Day 0																Day 1				Day 2	Day 3		
**Time, hours**	**0**	**15** min	**30** min	**1**	**1.5**	**2**	**2.5**	**3**	**3.5**	**4**	**5**	**6**	**8**	**10**	**12**	**18**	**24**	**30**	**36**	**42**	**48**	**72**	**Day 7**	**Day 14**
**Parasite count**, 10ul Thumbprick or venous sample	X			X		X		X		X		X	X		X	X	X	X	X	X	X	X	X	X
**Pregnancy test**, women only	X																							X
**Temperature**	X					X				X		X	X		X	X	X	X	X	X	X	X	X	X
**PK sampling**, 2ml venous sample	X	X	X	X	X	X	X	X	X	X	X	X	X	X	X	X	X				X	X		
**Parasite viability**, 100ul	X			X		X				X			X		X		X							
**PCR sample**, 50ul	X																						X[1]	X[1]
**Safety blood sample,** 2ml venous sample	X																					X	X	[2]X

1. Only if parasites are present
2. Only if abnormal on day 7

14.2. Appendix 2: Children Time and Events Table

Children	Day 0																Day 1				Day 2	Day 3	day 7	day 14
**Time, hours**	**0**	**15** min	**30** min	**1**	**1.5**	**2**	**2.5**	**3**	**3.5**	**4**	**5**	**6**	**8**	**10**	**12**	**18**	**24**	**30**	**36**	**42**	**48**	**72**		
**Parasite count,** 10ul Thumbprick or venous sample	X											X			X	X	X		X	X	X	X	X	X
Temperature	X											X			X	X	X	X	X	X	X	X	X	X
**Parasite viability,** volume 100ul	X														X									
**PCR sample,** 50ul	X																						X[2]	X[2]
**Safety blood sample,** 2ml venous sample	X																					X	X	[1]X

1. Only if abnormal on day 7
2. Only if parasites are present

14.3. Appendix 3: Informed Consent Form; Malawi

SAMPLE INFORMED CONSENT STATEMENT

Study Title: An open, randomised, multi-centre, dose-ranging phase II study to evaluate the safety, efficacy and pharmacokinetics of LAPDAP in combination with three different doses of artesunate.

Principal Investigator: Prof. M.E. Molyneux

The consent form will be amended to local guidelines and will be approved by local Ethics Review Committees. The following list of statements need to be present:

- You, or your child, are being asked to take part in a research project. If you agree you, or your child, will be examined to see whether you, or your child, are in good health. All results will be kept confidential.
- In this project you, or your child, will be given either one or two treatments for malaria, both are known to work well and are well tolerated (**LAPDAP** +/- artesunate).
- There may be advantages in giving both of these drugs together, but we need to find out how much to give of the second drug. You, or your child, may get the first drug alone (**LAPDAP**), or **LAPDAP** plus the second drug (artesunate) in one of three doses. You, or your child, will be randomly assigned to one of these treatments.
- All drugs have side effects. The unwanted effects reported with these drugs have been mild and not serious, for example **LAPDAP** has been associated with cough, vomiting, abdominal pain, anorexia, pruritus and diarrhoea. Other events associated with the use of **LAPDAP** include reductions in haemoglobin and/or haematocrit, haemolytic anaemia, methaemoglobinaemia (elevations sufficient to produce clinical symptoms have not been observed), reduction in platelet counts, and increases in liver transaminases. Artesunate is generally well tolerated; it may cause abdominal cramps and diarrhoea and, rarely slowing of the pulse rate. Increases in blood tests of liver function and lowered red blood cells may occasionally occur. We anticipate that both drugs will work, and they may work better when given together.
- You, or your child, do not have to take part in this study, and if you say "no", you, or your child, will still receive normal standard of care.
- If you agree to taking part in this study by saying ‘yes’ you or your child, may change your mind and withdraw from the study at any time, and you, or your child, will receive our normal standard of care.
- A total of 240 subjects will be asked to take part in this project.
- If you agree to take part in this project, you or your child, will be needed to stay in the hospital for four days. All adult subjects will have a needle placed in their hand from which blood samples can be taken. About 19 small samples will be needed

during your stay. Children who participate in the study will have 2 small samples taken using a needle (on day 0 and 7), and 10 smaller samples from a finger prick.

- We will then need to see you or your child again after 4 days, and again in one week to see if you or your child are well. Small blood samples may also be taken at these visits.

If the subject is unable to read then a witness (someone unconnected with the investigators team) should sign to confirm that the subject has had the information read to them and that they understand.

All children being invited to participate in this study should be accompanied by a parent or guardian, who is able to give consent on behalf of the child. They will be asked to give consent for the child in their care to participate in the study.

Complete part 1 or 2.

1. Written Consent

Statement of consent: I have read and understand all the information describing this study and all my questions have been answered to my satisfaction.

I voluntarily consent to participate in the study / I voluntarily consent for the child in my care, named below, to participate in the study.

Subject name ________________________________

Subject signature____________________________**Date** ______________DD/MMM/YY
Subject / other (specify); or thumbprint

Investigator name ________________________________
(Person obtaining consent)
Investigator signature________________________**Date**______________DD/MMM/YY

OR

2. Oral witnessed consent

I confirm that I have explained the information describing the study to the subject, or their guardian, and answered all their questions.

They have given their verbal consent to take part in this study / they have given their verbal consent for the child in their care, named below, to participate in this study.

Investigator name ______________________________
(Person obtaining consent)
Investigator signature____________________**Date**______________DD/MMM/YY

Subject name ________________________________

I confirm that the investigator has completely described the study to the subject (and their guardian if applicable).

Name of witness________________________________

Witness signature_________________________**Date** ____________DD/MMM/YY
(or thumbprint)

A copy of this should be given to the subject and the original kept in the investigators study file

14.4. Appendix 4: Informed Consent Form; The Gambia

SAMPLE INFORMED CONSENT STATEMENT

Study Title: An open, randomised, multi-centre, dose-ranging phase II study to evaluate the safety, efficacy and pharmacokinetics of LAPDAP in combination with three different doses of artesunate.

Principal Investigator: Dr Sam Dunyo

The consent form will be amended to local guidelines and will be approved by local Ethics Review Committees. The following list of statements need to be present:

- You are being asked to take part in a research project. If you agree you will be examined to see whether you are in good health. All results will be kept confidential.
- In this project you will be given either one or two treatments for malaria, both are known to work well and are well tolerated (**LAPDAP** +/- artesunate).
- There may be advantages in giving both of these drugs together, but we need to find out how much to give of the second drug. You may get the first drug alone (**LAPDAP**), or **LAPDAP** plus the second drug (artesunate) in one of three doses. You will be randomly assigned to one of these treatments.
- All drugs have side effects. The unwanted effects reported with these drugs have been mild and not serious, for example **LAPDAP** has been associated with cough, vomiting, abdominal pain, anorexia, pruritus and diarrhoea. Other events associated with the use of **LAPDAP** include reductions in haemoglobin and/or haematocrit, haemolytic anaemia, methaemoglobinaemia (elevations sufficient to produce clinical symptoms have not been observed), reduction in platelet counts, and increases in liver transaminases. Artesunate is generally well tolerated; it may cause abdominal cramps and diarrhoea and, rarely slowing of the pulse rate. Increases in blood tests of liver function and lowered red blood cells may occasionally occur. We anticipate that both drugs will work, and they may work better when given together.
- You do not have to take part in this study, and if you say "no", you will still receive normal standard of care.
- If you agree to taking part in this study by saying ‘yes’ you may change your mind and withdraw from the study at any time, and you will receive our normal standard of care.
- A total of 240 subjects will be asked to take part in this project.
- If you agree to take part in this project you will be needed to stay in the hospital for four days. All adult subjects will have a needle placed in their hand from which blood samples can be taken. About 19 small samples will be needed during your stay.

- We will then need to see you again after 4 days, and again in one week to see if you are well. Small blood samples may also be taken at these visits.

If the subject is unable to read then a witness (someone unconnected with the investigators team) should sign to confirm that the subject has had the information read to them and that they understand.

Complete part 1 or 2.

3. Written Consent

Statement of consent: I have read and understand all the information describing this study and all my questions have been answered to my satisfaction.

I voluntarily consent to participate in the study.

Subject name ________________________________

Subject signature___________________________Date _____________DD/MMM/YY
(or thumbprint)

Investigator name ________________________________
(Person obtaining consent)
Investigator signature_______________________Date______________DD/MMM/YY

OR

4. Oral witnessed consent

I confirm that I have explained the information describing the study to the subject and answered all their questions.

They have given their verbal consent to take part in this study.

Investigator name ______________________________
(Person obtaining consent)
Investigator signature___________________Date______________DD/MMM/YY

Subject name ________________________________

I confirm that the investigator has completely described the study to the

Name of witness________________________________

Witness signature________________________Date ____________DD/MMM/YY
(or thumbprint)

A copy of this should be given to the subject and the original kept in the investigators study file

14.5. Appendix 5: Dosing charts

Group A = 2mg/kg CPG, 2.5 mg/kg DDS and 0 Artesunate.

Weight Kg	LAPDAP 80/100 tablets	ART 50mg	ART 25mg	ART 10mg	ART 1mg	actual ART dose	actual ART mg/kg
85	2	0	0	0	0	0	0
84	2	0	0	0	0	0	0
83	2	0	0	0	0	0	0
82	2	0	0	0	0	0	0
81	2	0	0	0	0	0	0
80	2	0	0	0	0	0	0
79	2	0	0	0	0	0	0
78	2	0	0	0	0	0	0
77	2	0	0	0	0	0	0
76	2	0	0	0	0	0	0
75	2	0	0	0	0	0	0
74	2	0	0	0	0	0	0
73	2	0	0	0	0	0	0
72	2	0	0	0	0	0	0
71	2	0	0	0	0	0	0
70	2	0	0	0	0	0	0
69	2	0	0	0	0	0	0
68	2	0	0	0	0	0	0
67	2	0	0	0	0	0	0
66	2	0	0	0	0	0	0
65	2	0	0	0	0	0	0
64	2	0	0	0	0	0	0
63	2	0	0	0	0	0	0
62	2	0	0	0	0	0	0
61	2	0	0	0	0	0	0
60	2	0	0	0	0	0	0
59	1.5	0	0	0	0	0	0
58	1.5	0	0	0	0	0	0
57	1.5	0	0	0	0	0	0
56	1.5	0	0	0	0	0	0
55	1.5	0	0	0	0	0	0
54	1.5	0	0	0	0	0	0
53	1.5	0	0	0	0	0	0
52	1.5	0	0	0	0	0	0
51	1.5	0	0	0	0	0	0
50	1.5	0	0	0	0	0	0
49	1.5	0	0	0	0	0	0
48	1.5	0	0	0	0	0	0
47	1.5	0	0	0	0	0	0
46	1.5	0	0	0	0	0	0
45	1.5	0	0	0	0	0	0
44	1	0	0	0	0	0	0

Group A -continued.

Weight Kg	LAPDAP 80/100 tablets	ART 50mg	ART 25mg	ART 10mg	ART 1mg	actual ART dose	actual ART mg/kg
43	1	0	0	0	0	0	0
42	1	0	0	0	0	0	0
41	1	0	0	0	0	0	0
40	1	0	0	0	0	0	0
39	1	0	0	0	0	0	0
38	1	0	0	0	0	0	0
37	1	0	0	0	0	0	0
36	1	0	0	0	0	0	0
35	1	0	0	0	0	0	0
34	1	0	0	0	0	0	0
33	1	0	0	0	0	0	0
32	1	0	0	0	0	0	0
31	1	0	0	0	0	0	0
30	1	0	0	0	0	0	0
29	1	0	0	0	0	0	0
28	1	0	0	0	0	0	0
27	1	0	0	0	0	0	0
26	1	0	0	0	0	0	0
25	1	0	0	0	0	0	0
24	1/2	0	0	0	0	0	0
23	1/2	0	0	0	0	0	0
22	1/2	0	0	0	0	0	0
21	1/2	0	0	0	0	0	0
20	1/2	0	0	0	0	0	0
19	1/2	0	0	0	0	0	0
18	1/2	0	0	0	0	0	0
17	1/2	0	0	0	0	0	0
16	1/2	0	0	0	0	0	0

Weight Kg	LAPDAP 15/18.75tablets	ART 50mg	ART 25mg	ART 10mg	ART 1mg	actual ART dose	actual ART mg/kg
15	2	0	0	0	0	0	0
14	2	0	0	0	0	0	0
13	2	0	0	0	0	0	0
12	2	0	0	0	0	0	0
11	1.5	0	0	0	0	0	0
10	1.5	0	0	0	0	0	0
9	1.5	0	0	0	0	0	0
8	1.5	0	0	0	0	0	0
7	1	0	0	0	0	0	0
6	1	0	0	0	0	0	0
5	1	0	0	0	0	0	0

Group B = 2mg/kg CPG, 2.5 mg/kg DDS and 1 mg/kg artesunate.

Weight Kg	LAPDAP 80/100	ART 50mg	ART 25mg	ART 10mg	ART 1mg	actual ART dose	actual ART mg/kg
85	2	1	0	4	0	90	1.06
84	2	1	0	3	0	80	0.95
83	2	1	0	3	0	80	0.96
82	2	1	0	3	0	80	0.98
81	2	1	0	3	0	80	0.99
80	2	1	0	3	0	80	1.00
79	2	1	0	3	0	80	1.01
78	2	1	0	3	0	80	1.03
77	2	1	0	3	0	80	1.04
76	2	1	0	3	0	80	1.05
75	2	1	0	3	0	80	1.07
74	2	1	0	3	0	80	1.08
73	2	1	0	2	0	70	0.96
72	2	1	0	2	0	70	0.97
71	2	1	0	2	0	70	0.99
70	2	1	0	2	0	70	1.00
69	2	1	0	2	0	70	1.01
68	2	1	0	2	0	70	1.03
67	2	1	0	2	0	70	1.04
66	2	1	0	2	0	70	1.06
65	2	1	0	2	0	70	1.08
64	2	1	0	2	0	70	1.09
63	2	1	0	1	0	60	0.95
62	2	1	0	1	0	60	0.97
61	2	1	0	1	0	60	0.98
60	2	1	0	1	0	60	1.00
59	1.5	1	0	1	0	60	1.02
58	1.5	1	0	1	0	60	1.03
57	1.5	1	0	1	0	60	1.05
56	1.5	1	0	1	0	60	1.07
55	1.5	1	0	1	0	60	1.09
54	1.5	1	0	1	0	60	1.11
53	1.5	1	0	0	0	50	0.94
52	1.5	1	0	0	0	50	0.96
51	1.5	1	0	0	0	50	0.98
50	1.5	1	0	0	0	50	1.00
49	1.5	1	0	0	0	50	1.02
48	1.5	1	0	0	0	50	1.04
47	1.5	0	1	2	0	45	0.96
46	1.5	0	1	2	0	45	0.98
45	1.5	0	1	2	0	45	1.00
44	1	0	1	2	0	45	1.02

Group B Continued.

Weight Kg	LAPDAP 80/100	ART 50mg	ART 25mg	ART 10mg	ART 1mg	actual ART dose	actual ART mg/kg
43	1	0	1	2	0	45	1.05
42	1	0	0	4	0	40	0.95
41	1	0	0	4	0	40	0.98
40	1	0	0	4	0	40	1.00
39	1	0	0	4	0	40	1.03
38	1	0	0	4	0	40	1.05
37	1	0	0	4	0	40	1.08
36	1	0	1	1	0	35	0.97
35	1	0	1	1	0	35	1.00
34	1	0	1	1	0	35	1.03
33	1	0	0	3	0	30	0.91
32	1	0	0	3	0	30	0.94
31	1	0	0	3	0	30	0.97
30	1	0	0	3	0	30	1.00
29	1	0	0	3	0	30	1.03
28	1	0	0	3	0	30	1.07
27	1	0	1	0	0	25	0.93
26	1	0	1	0	0	25	0.96
25	1	0	1	0	0	25	1.00
24	1/2	0	1	0	0	25	1.04
23	1/2	0	1	0	0	25	1.09
22	1/2	0	0	2	0	20	0.91
21	1/2	0	0	2	0	20	0.95
20	1/2	0	0	2	0	20	1.00
19	1/2	0	0	2	0	20	1.05
18	1/2	0	0	2	0	20	1.11
17	1/2	0	0	2	0	20	1.18
16	1/2	0	0	2	0	20	1.25

Weight Kg	LAPDAP 15/18.75	ART 50mg	ART 25mg	ART 10mg	ART 1mg	actual ART dose	actual ART mg/kg
15	2	0	0	1	5	15	1.00
14	2	0	0	1	4	14	1.00
13	2	0	0	1	3	13	1.00
12	2	0	0	1	2	12	1.00
11	1.5	0	0	1	1	11	1.00
10	1.5	0	0	1	0	10	1.00
9	1.5	0	0	1	0	10	1.11
8	1.5	0	0	1	0	10	1.25
7	1	0	0	0	7	7	1.00
6	1	0	0	0	6	6	1.00
5	1	0	0	0	5	5	1.00

Group C = 2mg/kg CPG, 2.5 mg/kg DDS and 2mg/kg artesunate.

Weight Kg	LAPDAP 80/100	ART 50mg	ART 25mg	ART 10mg	ART 1mg	actual ART dose	actual ART mg/kg
85	2	3	0	2	0	170	2.00
84	2	3	0	2	0	170	2.02
83	2	3	0	2	0	170	2.05
82	2	3	0	1	0	160	1.95
81	2	3	0	1	0	160	1.98
80	2	3	0	1	0	160	2.00
79	2	3	0	1	0	160	2.03
78	2	3	0	1	0	160	2.05
77	2	3	0	0	0	150	1.95
76	2	3	0	0	0	150	1.97
75	2	3	0	0	0	150	2.00
74	2	3	0	0	0	150	2.03
73	2	3	0	0	0	150	2.05
72	2	3	0	0	0	150	2.08
71	2	2	0	4	0	140	1.97
70	2	2	0	4	0	140	2.00
69	2	2	0	4	0	140	2.03
68	2	2	1	1	0	135	1.99
67	2	2	1	1	0	135	2.01
66	2	2	0	3	0	130	1.97
65	2	2	0	3	0	130	2.00
64	2	2	0	3	0	130	2.03
63	2	2	1	0	0	125	1.98
62	2	2	1	0	0	125	2.02
61	2	2	1	0	0	125	2.05
60	2	2	0	2	0	120	2.00
59	1.5	2	0	2	0	120	2.03
58	1.5	2	0	2	0	120	2.07
57	1.5	2	0	1	0	110	1.93
56	1.5	2	0	1	0	110	1.96
55	1.5	2	0	1	0	110	2.00
54	1.5	2	0	1	0	110	2.04
53	1.5	2	0	1	0	110	2.08
52	1.5	2	0	0	0	100	1.92
51	1.5	2	0	0	0	100	1.96
50	1.5	2	0	0	0	100	2.00
49	1.5	2	0	0	0	100	2.04
48	1.5	1	1	2	0	95	1.98
47	1.5	1	1	2	0	95	2.02
46	1.5	1	1	2	0	95	2.07
45	1.5	1	0	4	0	90	2.00
44	1	1	0	4	0	90	2.05

Group C Continued.

Weight Kg	LAPDAP 80/100	ART 50mg	ART 25mg	ART 10mg	ART 1mg	actual ART dose	actual ART mg/kg
43	1	1	1	1	0	85	1.98
42	1	1	1	1	0	85	2.02
41	1	1	1	1	0	85	2.07
40	1	1	0	3	0	80	2.00
39	1	1	0	3	0	80	2.05
38	1	1	1	0	0	75	1.97
37	1	1	1	0	0	75	2.03
36	1	1	0	2	0	70	1.94
35	1	1	0	2	0	70	2.00
34	1	1	0	2	0	70	2.06
33	1	1	0	2	0	70	2.12
32	1	1	0	1	4	64	2.00
31	1	1	0	1	2	62	2.00
30	1	1	0	1	0	60	2.00
29	1	1	0	1	0	60	2.07
28	1	1	0	1	0	60	2.14
27	1	1	0	0	4	54	2.00
26	1	1	0	0	2	52	2.00
25	1	1	0	0	0	50	2.00
24	1/2	1	0	0	0	50	2.08
23	1/2	0	1	2	1	46	2.00
22	1/2	0	1	2	0	45	2.05
21	1/2	0	0	4	2	42	2.00
20	1/2	0	0	4	0	40	2.00
19	1/2	0	0	4	0	40	2.11
18	1/2	0	1	1	0	35	1.94
17	1/2	0	1	1	0	35	2.06
16	1/2	0	0	3	2	32	2.00

Weight Kg	LAPDAP 15/18.75	ART 50mg	ART 25mg	ART 10mg	ART 1mg	actual ART dose	actual ART mg/kg
15	2	0	0	3	0	30	2.00
14	2	0	1	0	3	28	2.00
13	2	0	1	0	1	26	2.00
12	2	0	1	0	0	25	2.08
11	1.5	0	0	2	2	22	2.00
10	1.5	0	0	2	0	20	2.00
9	1.5	0	0	2	0	20	2.22
8	1.5	0	0	1	6	16	2.00
7	1	0	0	1	4	14	2.00
6	1	0	0	1	2	12	2.00
5	1	0	0	1	0	10	2.00

Group D = 2mg/kg CPG, 2.5 mg/kg DDS and 4mg/kg artesunate.

Weight Kg	LAPDAP 80/100	ART 50mg	ART 25mg	ART 10mg	ART 1mg	actual ART dose	actual ART mg/kg
85	2	6	1	1	0	335	3.94
84	2	6	1	1	0	335	3.99
83	2	6	1	1	0	335	4.04
82	2	6	1	0	0	325	3.96
81	2	6	1	0	0	325	4.01
80	2	6	0	2	0	320	4.00
79	2	6	0	2	0	320	4.05
78	2	6	0	1	0	310	3.97
77	2	6	0	1	0	310	4.03
76	2	6	0	1	0	310	4.08
75	2	6	0	0	0	300	4.00
74	2	6	0	0	0	300	4.05
73	2	5	1	2	0	295	4.04
72	2	5	1	1	0	285	3.96
71	2	5	1	1	0	285	4.01
70	2	5	1	0	0	275	3.93
69	2	5	1	0	0	275	3.99
68	2	5	0	2	0	270	3.97
67	2	5	0	2	0	270	4.03
66	2	5	0	1	0	260	3.94
65	2	5	0	1	0	260	4.00
64	2	5	0	1	0	260	4.06
63	2	5	0	0	0	250	3.97
62	2	5	0	0	0	250	4.03
61	2	4	2	0	0	250	4.10
60	2	4	1	1	0	235	3.92
59	1.5	4	0	3	0	230	3.90
58	1.5	4	1	1	0	235	4.05
57	1.5	4	1	0	0	225	3.95
56	1.5	4	0	2	0	220	3.93
55	1.5	4	0	2	0	220	4.00
54	1.5	4	0	2	0	220	4.07
53	1.5	4	0	1	0	210	3.96
52	1.5	4	0	1	0	210	4.04
51	1.5	4	0	0	0	200	3.92
50	1.5	4	0	0	0	200	4.00
49	1.5	3	0	4	0	190	3.88
48	1.5	3	0	4	0	190	3.96
47	1.5	3	0	3	0	180	3.83
46	1.5	3	0	3	0	180	3.91
45	1.5	3	0	3	0	180	4.00
44	1	3	0	3	0	180	4.09

Group D - continued.

Weight Kg	LAPDAP 80/100	ART 50mg	ART 25mg	ART 10mg	ART 1mg	actual ART dose	actual ART mg/kg
43	1	3	0	2	0	170	3.95
42	1	3	0	2	0	170	4.05
41	1	3	0	1	0	160	3.90
40	1	3	0	1	0	160	4.00
39	1	3	0	1	0	160	4.10
38	1	3	0	0	0	150	3.95
37	1	3	0	0	0	150	4.05
36	1	2	1	2	0	145	4.03
35	1	2	0	4	0	140	4.00
34	1	2	1	1	0	135	3.97
33	1	2	0	3	0	130	3.94
32	1	2	0	3	0	130	4.06
31	1	2	1	0	0	125	4.03
30	1	2	0	2	0	120	4.00
29	1	2	0	2	0	120	4.14
28	1	2	0	1	0	110	3.93
27	1	2	0	1	0	110	4.07
26	1	2	0	1	0	110	4.23
25	1	2	0	0	0	100	4.00
24	1/2	1	1	2	0	95	3.96
23	1/2	1	1	2	0	95	4.13
22	1/2	1	0	4	0	90	4.09
21	1/2	1	1	1	0	85	4.05
20	1/2	1	0	3	0	80	4.00
19	1/2	1	1	0	0	75	3.95
18	1/2	1	0	2	2	72	4.00
17	1/2	1	0	2	0	70	4.12
16	1/2	0	1	4	0	65	4.06

Weight Kg	LAPDAP 15/18.75	ART 50mg	ART 25mg	ART 10mg	ART 1mg	actual ART dose	Actual ART mg/kg
15	2	1	0	1	0	60	4.00
14	2	0	1	3	0	55	3.93
13	2	1	0	0	2	52	4.00
12	2	1	0	0	0	50	4.17
11	1.5	0	1	2	0	45	4.09
10	1.5	0	0	4	0	40	4.00
9	1.5	0	1	1	0	35	3.89
8	1.5	0	0	3	2	32	4.00
7	1	0	1	0	3	28	4.00
6	1	0	1	0	0	25	4.17
5	1	0	0	2	0	20	4.00

14.6. Appendix 6: Protocol Amendment 1

The changes made in this amendment are summarised below. Changes to the protocol are identified by the following conventions: additional text is in *italics*, removed text is identified by using ~~strikethrough~~.

Pregnancy Tests

At the request of GSK's Global Safety Board, pregnancy testing on day 0, at screening has been changed from a urinary test to a serum test. This will require that consent is obtained for a blood sample to be taken on day 0, prior to informed consent for participation the study. Serum pregnancy tests have greater sensitivity (10IU/ml βhCG) than urine tests (20 or 25IU/ml βhCG).

Section 5.1.1, Pregnancy testing

> All women of child-bearing potential will be asked to take a *serum* ~~urinary~~ pregnancy test at screening for the study. If consent is not given to take a pregnancy test, they will not be eligible to participate in the study. A second *urinary* pregnancy test will be performed on day 14.

Change in PK analytical techniques

The responsibility for PK sample analysis has moved in-house and a new analytical technique been developed for CPG, DDS, ART and their major metabolites CCG, MADDS and DHA respectively. This has allowed a reduction in the total volume of blood being drawn at each sampling point (from 5ml to 2ml) and changes in handling procedures:

Section 6.2 Study Visits

> Day 0:
> ~~Prior to dosing a sample of <7.5ml will be taken from adults, and <2.5 ml from children, for the following time 0 samples:~~
>
> ~~• 5ml PK sample from adults only~~
>
> *Prior to dosing a sample of <4.5ml will be taken from adults, and <2.5 ml from children, for the following time 0 samples:*
>
> - 2ml PK sample from adults only

Section 6.5, Pharmacokinetics, Method for handling PK blood samples

> Blood, approx 2ml, will be collected into polypropylene tubes containing lithium heparin, mixed gently and placed on crushed wet-ice for no longer than 30mins, until centrifugation at 1800g for 10 minutes using a refrigerated centrifuge. The resultant

plasma will be separated, and transferred in equal volumes to two uniquely labelled clear polypropylene tubes and frozen immediately over solid carbon dioxide or in a freezer at nominal –80°C. Samples will be transported frozen to WW Bioanalysis, DMPK, Ware, to be stored at approximately -80°C until analysed. Plasma concentrations of CPG, CCP, DDS, MADDS, ART and DHA will be determined using currently approved methods by the WW Bioanalysis group, Ware and be used to evaluate T_{max}, C_{max}, $t_{1/2}$, AUC_{24} and AUC_{∞} if data permit.

Assessment of reticulocytes

A limited amount of data from the phase I, FTIM study indicated that those patients who received Artesunate or Lapdap plus Artesunate in their third dosing phase had a reduction in their reticulocyte levels at 96-hours post-dose. This amendment is necessary to add reticulocyte assessments on day 0, 3 and 7. Day 14 assessments will be done if day 7 results are abnormal. Red blood cell counts will be required to calculate the actual reticulocyte levels.

Section 6.3, Safety

Table 2 – added a row for reticulocyte counts and red blood cell counts. Amended full biochemistry and haematology assessments for both adults and children on day 3.

Removal of paracetamol from prohibited medications.

It was recognised that withholding paracetamol, or any other analgesic from patients with malaria would increase the risk of fits, convulsions and progression of the disease into severe malaria. Paracetamol is the drug of choice locally and has therefore been added to the list of permitted medications.

Section 8.1, Permitted Medications

> All subjects can be given *paracetamol or* codeine as an analgesic during the *study, at the discretion of the treating physician.* Allowable antibiotics are penicillin, cephalosporins and aminoglycocides.

Section 8.2, Prohibited medications

> Any antimalarial, or antibiotic with antimalarial activity (erythromycin and other macrolides, co-trimoxazole and other sulphonamides, any tetracycline (including doxycycline, and quinolones. ~~Antipyretic and non-steroidal anti-inflammatory drugs (including paracetamol, salicylates and ibuprofen).~~

PCR analysis

It was noted that the section describing Early and Late treatment failure referenced PCR analysis of parasite strains to determine if infections were recrudescences or re-infections, but the collection of filter papers for this analysis was omitted. This has been added to the protocol in the following locations:

Section 4, Study design.

A row has been added to Table 1. Time and Events, to indicate PCR sampling on day 0, and then days 7 and 14 if parasites are detected on these visits.

Section 6.2, Study Visits, Day 0

- 2ml PK sample from adults only
- 2ml biochemistry and haematology, all subjects (see Table 2, section 6.3)
- 10ul for parasitaemia blood slide, all subjects
- *50ul for PCR filter paper preparation (see section 6.4.4)*
- 100ul for parasite viability, all subjects

Section 6.2, days 7 and 14. The following sentence has been inserted:

If parasites are detected, a filter paper should be prepared for PCR analysis.

Section 6.4.4, PCR analysis; section inserted:

PCR filter papers will be prepared on day 0, then day 7 or 14 if parasites are detected, or on any unscheduled visit where the subject is thought to fulfil the criteria for late treatment failure described in section 6.4.3. Two drops (approx 50ul) of blood will be collected on pre-printed filter paper and allowed to dry. Once dry samples will be placed in small plastic bags and stored at 4°C until analysis.

Definition of Early Treatment Failure.

It was recognised that the current protocol definition of early treatment failure would be impractical to implement in an in-patient setting. The current definition is based on out-patient assessments as defined by the WHO. The following adaptation has been made

Section 6.4.3, Treatment failure

Early treatment failure

- The development of severe malaria syndrome or parasite density > 250,000/ul at any time on days 0, 1 , 2 or 3. ~~The need for admission to hospital for the treatment of parasitaemic illness on days 0, 1, 2 or 3.~~
- Evidence between 48 and 72 hours of the trial (day 2) that the subject is symptomatic **and** has a parasitaemia greater than the value recorded at entry to the trial. ~~Evidence that the subject is symptomatic **and** parasitaemia greater than the value recorded at entry to the trial.~~
- Parasitaemia between 72 and 96 hours of the trial (day 3) greater than 25% of the value recorded on entry to the trial. ~~Parasitaemia on day 3 greater than 25% of the value recorded on entry to the trial.~~

14.7. Appendix 7: Protocol amendment 2.

The changes made in this amendment are summarised below, the majority of which were identified when writing / reviewing the Reporting and Analysis Plan. Changes made to the protocol are made by the following conventions: additional text is in *italics*, removed text is identified using ~~strikethrough~~.

1. Change of analysis populations.
The definition of the intent-to-treat (ITT) population has been modified to match the safety population - therefore only two populations will be included in the final analysis: ITT and per-protocol (PP). Additionally, the PP population will now be considered as the primary population for efficacy evaluations.

Rationale:
The protocol originally stated that the ITT population would be 'all subjects randomised to receive study medication'. However this was not felt to be consistent with the ITT definition that is likely to be used in future Phase III development for this compound and so it has been amended. As this is a dose selection study, a decision has been made to use the PP population as the primary population for efficacy, rather than the ITT population, as this is felt to allow a purer assessment of efficacy for dose selection. The ITT population will now be used to confirm efficacy findings and assess safety.

Section 11.1 Hypotheses
The **null hypothesis** for this study is:
There is no difference between **LAPDAP** administered alone and **LAPDAP** administered in conjunction with artesunate in PC90 in the ~~intention-to-treat~~ *per-protocol* population.

The **alternative hypothesis** for this study is:
There is a difference between **LAPDAP** administered alone and **LAPDAP** administered in conjunction with artesunate in PC90 in the ~~intention-to-treat~~ *per-protocol* population.

Section 11.2.1 Primary Comparisons of Interest
The primary comparison is the PC90 for the ~~ITT~~ *PP* population, in adults only. PC90 is the time to achieve a reduction of the parasitaemia to 90% of baseline.

Section 11.5 Analysis Populations
Intent-to-treat population
~~All subjects randomised to study medication will qualify for the 'intention'to'treat' analysis. The time between randomisation and administration of treatment will be minimised.~~ *All subjects who were randomised and have received any dose of the study medication, irrespective of whether they vomited, will be included in the intent-to-treat population. The intent-to-treat analysis of efficacy will be used to confirm findings from the primary efficacy analysis and will be the population used for safety analyses.*

Per-protocol population
~~The per-protocol analysis of efficacy will be used to confirm the findings from the intent-to-treat population.~~ *The per-protocol population will be used as the primary population*

for efficacy analyses. Subjects will be eligible for the per-protocol efficacy analysis providing the criteria in the intent to treat population has been satisfied and the following apply:

- The subject has completed all visits as specified by the protocol
- No major protocol violation exists with regard to Inclusion/Exclusion criteria
- No prohibited concomitant medications were taken during the treatment period

~~Safety population~~
~~All subjects who have received any dose of the study medication will be included in the safety population.~~

Furthermore, the ITT population has replaced all references to the safety population in the protocol.

Section 11.7 Efficacy Analyses
Summaries of the primary and secondary endpoints will be presented for both the intent~~ion~~-to-treat and per-protocol populations. The ~~intent-to-treat~~ *per-protocol* population will be considered as the primary population for efficacy evaluations.

2. Addition of a secondary end-point of gametocyte analysis
The addition of this endpoint will not require any additional study procedures, as the data will be obtained from the microscope slides prepared for parasitology assessment. Any subjects already completing the study at the time of implementation of this protocol amendment will have their slides read for gametocytes retrospectively.

Rationale:
The artemisinins have been demonstrated to reduce the number of circulating gametocytes in combination with other anti-malarial drugs. This is advantageous as a reduction in gametocytes levels could potentially lower the rate of transmission within a population, although may not be of direct benefit to the individual. Measuring the presence and level of gametocytes following administration of the different doses of artesunate in this study is therefore an important criterion in dose selection as recommended by the WHO [World Health Organisation , 2001]. We also anticipate that we will be able to demonstrate a clear benefit of adding artesunate to Lapdap by evaluating gametocyte clearance.

Protocol Summary
Secondary Endpoints:

- PC50 and PC99 determined from blood slide data, determined from 'rich' adult data and confirmed with more sparse child data.
- *Proportion gametocytaemic and gametocyte density, all subjects.*
- Early or late treatment failures (WHO Definitions), all subjects

Section 3.2.1 Efficacy

- PC50 and PC99 determined from blood slide data, determined from 'rich' adult data and confirmed with more sparse child data.
- *Proportion gametocytaemic and gametocyte density, all subjects.*
- Early or late treatment failures (WHO Definitions), all subjects.

Section 6.4.6 Gametocyte analysis
Slides prepared for parasitology will be read for the presence or absence of gametocytes per 200 white blood cells, on days 0 (pre-dose), 3, 7 and 14. If gametocytes are present, their density will be calculated and recorded as the number of gametocytes present per ul.

Section 11.2.2 Other Comparisons of Interest
The comparisons detailed in 11.2.1 will be made, using the closed testing procedure for the following secondary efficacy endpoints: PC50, PC90, parasite viability and early/late treatment failure. Details of these analyses are given in section 11.7.2. These will be presented for the ITT and PP populations.

The presence or absence of gametocytes (% of gametocytaemic subjects) will be summarised by artesunate dose. Additionally, summary statistics will be presented for the gametocyte density by artesunate dose.

Section 11.7.2 Secondary Analysis

In addition the linear trend of artesunate dose will be investigated by including a term for artesunate dose as a continuous variable in the analyses of variance.

The presence or absence of gametocytes (% of gametocytaemic subjects) will be summarised by artesunate dose. Additionally, summary statistics will be presented for gametocyte density by artesunate dose. However no formal statistical comparisons will be made.

Decision matrix for selection of dose

The following paragraph will be inserted after the paragraph on parasite viability & before that describing how the children's data will be handled.

It is intended that the analysis will proceed as described in the decision matrix, but a full clinical review of the safety data and gametocyte data will also be conducted to ensure that no overriding signals are overlooked when deciding the dose. For example, if there are safety signals in the 4mg/kg artesunate group, but not in the 2 mg/kg artesunate group, and both groups show similar findings for PC90 and PV, then the lower dose will be selected. Or, if gametocyte eradication is better in the 4 mg/kg artesunate group than the 2mg/kg artesunate group then the higher dose may be selected. Any deviation from the decision matrix will be justified and documented in the Clinical Study Report.

3. Modification of the safety endpoint definition for internal consistency.
In protocol amendment #1 the early treatment failure definition was modified to a count of >250,000ul^{-1}, however the related safety endpoint definition was not changed.

We are therefore clarifying this in the following places:
Section 3.2.2 Safety

- ~~Development of clinically severe malaria, including a parasite count of >100,000ul^{-1}~~
- *Development of clinically severe malaria, including a parasite count of >250,000ul^{-1}*

Section 11.8 Safety analyses

4. Modification of the Late Treatment Failure definition.

The late treatment failure definition is being modified a) to include a third definition of treatment failure, b) to remove the bullet point relating to adverse events and c) to correct the threshold of severe anaemia to reflect the practice at site.

Rationale:

b) The analysis of an adverse event severe enough to warrant no further drug exposure is inappropriate to be included as a late treatment failure, as the therapy is only administered during days 0, 1 and 2, which is prior to the time-period associated with late treatment failure.

c) The threshold for severe anaemia is being aligned with the threshold used by the site, 5g%, as opposed to 7g% as previously written. This is also consistent with the WHO definition of severe falciparum malaria [World Health Organisation , 2000].

Section 6.4.3 Treatment failure

Late treatment failure

- The need for admission to hospital for the treatment of parasitaemic illness between days 4 and 14 inclusive when: (a) the subject has not previously met the criteria for early treatment failure, and (b) PCR analysis of markers including MSP1 suggest that the parasites are unlikely to be a new infection.
- Evidence that the subject is symptomatic and any level of *P. falciparum* parasitaemia on any day between 4 and 6.
- *Any level of P. falciparum parasitaemia on any day between 7 and 14 inclusive, where subsequent PCR analysis of markers including MSP1 suggest that the parasites are unlikely to be a new infection.*

The following addition~~s have~~ *has* been made to the definition for late treatment failure:

~~An adverse event, which is thought to be drug-related, and severe enough to warrant no further drug exposure (adverse events are dealt with in section 11.8.2)~~

Development of severe anaemia (haemoglobin lower than ~~7~~*5*g%) in the presence or absence of parasitaemia *on days 4-14, inclusive.*

5. Extension of Inclusion Criteria ranges.
Feedback from the site indicates that the upper weight and age limits are restricting recruitment. There are no medical or safety concerns about increasing these limits and the following modifications have been agreed, which are within the limits of the ranges tested in prior Lapdap studies:

Section 5.2.1 Inclusion criteria
- Adults aged between 18 and ~~45~~*60* years, or children aged between 12 and 120 months
- Weight between 5 and ~~70~~*85*kg

The dosing tables have also been modified to include the extra weight bands and are not duplicated here to keep the document length to a minimum.

6. Pharmacokinetic Analysis
Additional pharmacokinetic analyses may be conducted, to examine the effect of artesunate on the pharmacokinetics of the components of Lapdap, if data permit.

Rationale:
We were unable to analyse the pharmacokinetic samples taken from an earlier phase I study. This study therefore presents us with an opportunity to explore if there are any pharmacokinetic effects of co-administering artesunate with Lapdap, compared to Lapdap alone, using the samples that are already being taken as part of the protocol.

Section 3.2.3 Pharmacokinetics

- Chlorproguanil (CPG), chlorcycloguanil (CCG), dapsone (DDS), mono acetyl dapsone (MADDS), artesunate (ART) and dihydroartemisinin (DHA) plasma levels, in adults only. These data will be used to determine T_{max}, C_{max}, $t_{1/2}$, and AUC for each compound, *and to assess the effect of artesunate on the pharmacokinetics of the components of Lapdap, if data permit.*

Section 6.5 Pharmacokinetics.

Additionally, the effect of artesunate on the pharmacokinetics of the components of Lapdap will be assessed, if data permit.

Section 11.9.1 Pharmacokinetic Analyses

Plasma will be used to evaluate pharmacokinetic parameters for CPG, CCG, DDS, MADDS, ART and DHA, including: T_{max}, C_{max}, $t_{1/2}$, AUC_{24} and AUC_{∞} if data permit. *Additional analyses of the effect of artesunate on the pharmacokinetics of the components of Lapdap may be conducted, if data permit.*

Protocol Amendment 2 References:

World Health Organisation (2000). Severe falciparum malaria. *Transactions of the Royal Society of Tropical Medicine and Hygeine*, **94**, supplement 1.

World Health Organisation (2001). Antimalarial Drug Combination Therapy. *Report of a WHO Technical Consultation.*

14.8. Appendix 8: Protocol amendment 3.

The changes made in this amendment are summarised below, which were identified as a result of slow recruitment into the study, especially the adults. Changes made to the protocol are made by the following conventions: additional text is in *italics*, removed text is identified using ~~strikethrough~~.

Rationale:

The inclusion criteria relating to parasite load has been reviewed, in light of slower recruitment into the study in the adults than expected. The lower parasitaemia limit for entry was initially set higher than usual in order to be able to determine PC90 (PC50 and PC99), as this measures the disappearance of parasites over time. On further review of available data, in consultation with experts in malaria research, it was agreed that a lower inclusion level would be appropriate for adults. Despite adult populations acquiring semi-immunity to malaria infections, it is recognised that the clinical symptoms of malaria are actually experienced at lower-level infections than in children. We therefore propose to reduce the entry level parasitaemia *in adults only* to 10,000ul^{-1}.

In order to compensate for the possible rapid clearance of parasites in the treatment arms receiving artesunate, we have added additional parasitology slide reading time-points in the adult group at 1 and 3-hours post-dose initial dose. This is to try to ensure that sufficient data is gathered for the estimation of parasite clearance time. Only one additional thumbprick will be required, as a 1-hour post dose blood sample is required already for parasite viability assessment. An extra 10ul of blood will be required for each additional blood slide.

It is also worth noting that at screening the parasitaemia is determined using a nominal WBC count of 8,000ul^{-1}, according to WHO protocol. However, for determining PC90, it is essential that the *actual* parasitaemia load is calculated using the individual's WBC count. The WBC count is only determined once the subject has consented to take part in the study, from the haematology laboratory results. It is recognised that if the WBC count for an individual is less than 8,000ul^{-1}, then the *actual* parasitaemia, when corrected, may be below the entry criteria level, for both adults and children.

For example, if an adult's actual WBC is half the assumed WBC (i.e. 4,000 instead of 8,000), then a screening parasitaemia level of 10,000ul^{-1} (uncorrected) would correspond to an *actual* parasitaemia level of 5,000ul^{-1}. Similarly for children, if a child's actual WBC is half the assumed WBC (i.e. 4,000 instead of 8,000), a screening parasitaemia level of 25,000ul^{-1} (uncorrected) would correspond to an *actual* parasitaemia level of 12,500ul^{-1}.

Therefore, any adult subject whose parasitaemia is below 5,000ul^{-1} once corrected at time 0 will be excluded from the per-protocol population, as it is anticipated that their parasite load may be too low to determine PC90. Adult subjects with screening parasitaemias that met the inclusion criteria of 10,000ul^{-1} and that have corrected parasitaemias at or above 5,000ul^{-1} will be eligible for the per-protocol population.

For children, a lower threshold of 12,500ul^{-1} for *actual* parasitaemias at time 0 will be applied, such that children with screening parasitaemias ≥25,000ul^{-1} and actual parasitaemias ≥12,500ul^{-1} will be eligible for the per-protocol population. Children with screening parasitaemias ≥ 25,000ul^{-1}, but *actual* parasitaemias <12,500ul^{-1} will not be eligible for the per-protocol population.

We anticipate that only 2 or 3 subjects will be excluded from the per-protocol population using these limits for *actual* parasitaemia levels.

Changes made:

Section 5.2.1 Inclusion criteria

Pure [on microscopic grounds] *screening* P. falciparum parasitaemia *in children from* 25,000 to 100,000 ul^{-1}*, or in adults from 10,000 to 100,000 ul*$^{-1}$*.*

Section 6.4.1. Parasite Count

Screening parasitaemia will be calculated using the nominal WBC count value of 8,000ul^{-1}*, according to WHO protocol. The actual parasitaemia will be corrected for the WBC count for each subject once the haematology results are available. The corrected parasitaemia value will be used as the baseline level for PC90, PC50, PC99 calculations and assessment of treatment failure.*

Should an adult's actual parasitaemia at time 0 be below 5,000ul^{-1}*, they will be excluded from the per-protocol population. Similarly, should a child's actual parasitaemia at time 0 be below 12,500ul*$^{-1}$*, they will be excluded from the per-protocol population.*

Table 3 has been amended to include additional samples at 1 and 3-hours post-dose, in adults only.

Section 11.5 Analysis Populations

Per-protocol population:

The per-protocol population will be used as the primary population for efficacy analyses. Subjects will be eligible for the per-protocol efficacy analysis providing the criteria in the intent-to-treat population has been satisfied and the following apply:

- The subject has completed all visits as specified by the protocol
- No major protocol violation exists with regard to Inclusion/Exclusion criteria
- No prohibited concomitant medications were taken during the treatment period
- *That an adult's actual parasitaemia level at time 0 was not below 5,000ul*$^{-1}$*, or*
- *That a child's actual parasitaemia level at time 0 was not below 12,500ul*$^{-1}$

14.9. Appendix 9: Protocol amendment 4

The changes made in this amendment are summarised below. Changes made to the protocol are made by the following conventions: additional text is in *italics*, removed text is identified using ~~strikethrough~~

Rationale:

A second site is being initiated in The Gambia, to help complete recruitment of adults. The Gambia's malaria season is complementary to Malawi's, running from September - December each year. Changes made to the protocol are mainly administrative in nature, adding the details of the second site, adding the commonly used anti-malarials in The Gambia to the exclusion criteria, adding centre as a variable to the analysis plans and providing an adult-specific informed consent form for The Gambia.

Prior use of chloroquine (CQ) within 28-days is being added to exclusion criteria 5, as CQ is first-line treatment in The Gambia. This will also apply to the Malawi site, although chloroquine is actively discouraged in Malawi. The addition of chloroquine to this exclusion criteria should not result in a different population of subjects being recruited at the two sites.

Co-trimoxazole is also being added to the exclusion criteria as it may interfere with the parasite viability assay. It was inadvertently overlooked in the original protocol as an exlcusion criteria, although it is listed as a prohibited medication. Communication from the active site in Malawi confirms that no one to date has received cotrimoxazole in the study, therefore the populations will be the same from both sites in this regard.

Changes made:

In all appropriate places the study title has been changed from 'An open, randomised, single centre dose-ranging phase II study to evaluate the safety, efficacy and pharmacokinetics of LAPDAP in combination with three different doses of artesunate' to *'An open, randomised, multi-centre, dose-ranging phase II study to evaluate the safety, efficacy and pharmacokinetics of LAPDAP in combination with three different doses of artesunate'*

Sponsor Information page:

Medical Monitors: William Mwatu, Yves Aphing-Kouassi

Protocol summary; study population:

120 Adults (men and women) and 120 children (between 12-120 months) with uncomplicated P. falciparum malaria will be recruited from *two sites* ~~a single centre~~*; one* in Malawi*, and a second in The Gambia, recruiting adults only.*

Section 5.1 Number of subjects:

This trial will be conducted in the Queen Elizabeth Central Hospital, Blantyre, Malawi *and at the Farafenni Field Station, MRC laboratories, The Gambia. The trial will be conducted* in 120 African adults (men and women) aged 18 – 45, and 120 African children aged 12 to 120 months with uncomplicated falciparum malaria.

Section 5.2.2. Exclusion criteria:

5. Treatment within the past twenty-eight days with sulphadoxine/pyrimethamine (Fansidar), sulfalene/pyrimethamine (Metakelfin), mefloquine-sulfadoxine-pyrimethamine (Fansimef), *chloroquine* (Nirupquine)*; 21-days with mefloquine, or 7-days with amodiaquine, halofantrine, quinine (full course), atovaquone-proguanil, artemisinins, co-artemether, tetracycline or clindamycin*, or 5 half-lives for any drugs with a potential anti-malarial activity (e.g. co-trimoxazole in the previous 60 hours).*

* Prior chloroquine use will be determined by questioning, with supporting evidence of CQ administration. If there is uncertainty about the identity of an anti-malarial taken in the previous 28-days, the subject will be asked to take a qualitative urinary test to determine if they have received chloroquine or not.

Section 8.2. Prohibited Medications

Chloroquine: *Prior treatment with chloroquine has been added to exclusion criteria 5, based on its use as first-line therapy in The Gambia in protocol amendment no. 4. This will apply to both the Malawi and Gambia sites.*~~Prior treatment with chloroquine will not be an exclusion criterion; however, I~~ Intercurrent use of chloroquine during the study period will be a protocol violation and cause exclusion from the analysis of the per-protocol group.

Section 11.7.1 Primary analysis:

The primary analysis will be performed in adults. Analyses of covariance will be used to analyse the primary variable (PC90) adjusting for the stratifying variable (gender), *centre* and treatment. Log transformed data will be used if necessary.

The interaction between gender and dose *and between centre and dose* will be investigated in secondary analyses, but will not be included in the closed testing procedure to determine the dose.

Section 11.7.2. Secondary analysis

A mixed effects model will be used to analyse the parasite count recorded post-dosing. Log transformed data will be used if necessary. Subject will be considered as a random variable (the interaction between subject and time will also be investigated). Terms for baseline parasite count, *centre*, gender in adults and age in children ~~(and other covariates specified in the RAP)~~ will be included in the model if significant at the 5% level. Treatment comparisons will be made in the same way as the primary efficacy endpoint. The results will also be shown graphically.

14.3 Appendix 3: Informed consent form; *Malawi*

14.4 Appendix 4: Informed consent form; The Gambia

Please refer to Appendix 4 for details of added text.
